# Supplementary material for: Transcription factor-mediated direct cellular reprogramming yields cell-type specific DNA methylation signature
Source: Sci Rep. 2023 Dec 15;13:22317. doi: 10.1038/s41598-023-49546-8 (PMC10724236; doi:10.1038/s41598-023-49546-8)
Supplement: Supplementary file 1 — Supplementary Information. [file 41598_2023_49546_MOESM1_ESM.pdf]

SUPPLEMENTARY INFORMATION

Transcription factor-mediated direct cellular reprogramming yields cell-type specific DNA methylation signature

Horisawa et al.

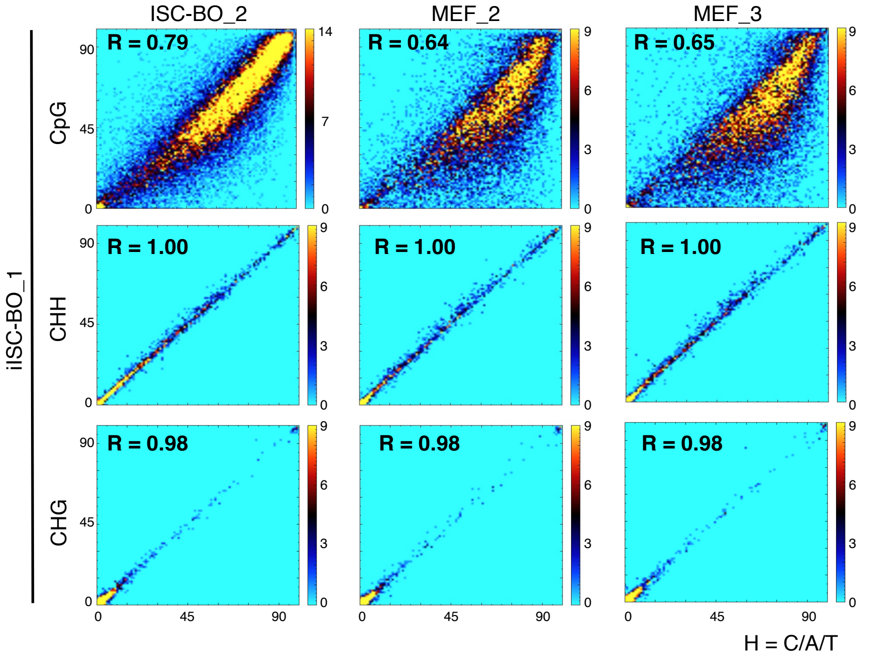

Supplementary Figure 1: Correlation of genome-wide CpG, CHH, and CHG methylation between samples

Density plots comparing genome-wide CpG, CHH, and CHG methylation status between samples. The window and step sizes were set to be 1 kbp and 500 bp, respectively. R scores indicate Pearson's correlation coefficient.

Supplementary Figure 2

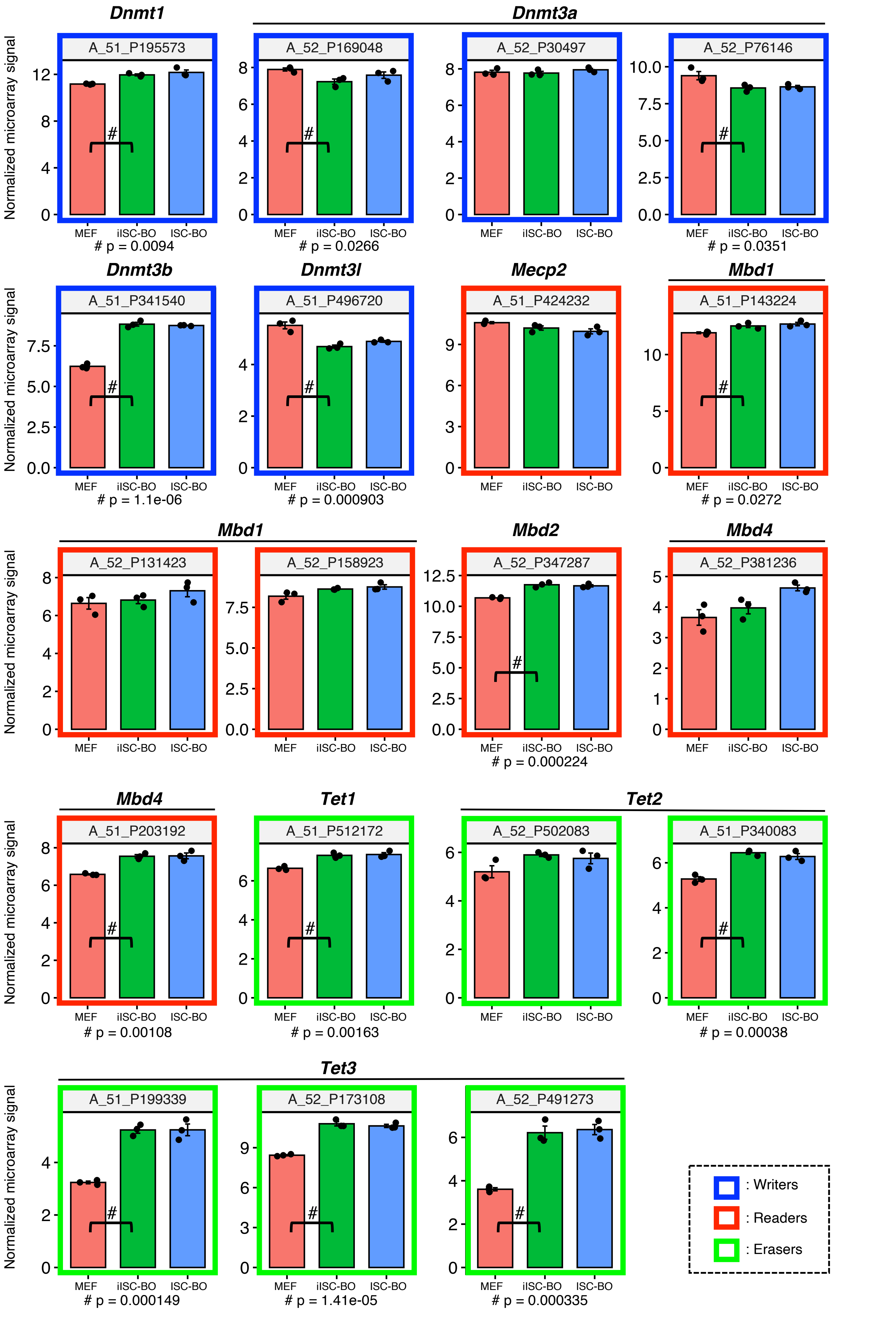

Supplementary Figure 2: Gene expression analysis for DNA methylation-related genes

Gene expression levels of DNA methylation-related genes. Vertical axes of all plots indicate normalized and log-transformed microarray signals. Probe IDs of the microarray corresponding to the transcripts are indicated at the top of each plot. The genes are categorized into 3 groups, *i.e.*, writers, readers, and erasers, with colors. Only for transcripts showing significant differences ($p < 0.05$) in expression between MEFs and iISC-BOs, the p-values are shown under each plot. Dunnett's test was used for statistical analysis.

Supplementary Table 1: Genes associated with iISC-high DMRs

Probe_ID	Gene_symbol	MEF_1	MEF_2	MEF_3	ISC_1	ISC_2	ISC_3	iISC_3	iISC_1	iISC_2
A_52_P168176	Scrt1	-0.9985949	0.47113354	0.76876	0.19360367	0.82445987	0.37471108	-1.7085827	-1.0605144	1.13502382
A_52_P268623	Samd4	-0.6385455	0.00447424	0.79279122	-0.6901589	2.23859683	-0.8460284	-0.201015	-0.8310762	0.17096174
A_52_P13365	Stk10	-0.1615611	-0.8595374	-0.310528	-0.1495168	1.89942559	-0.7069737	-0.7792766	-0.422868	1.49083594
A_51_P259118	Klhl1	-0.4138446	-0.5436637	-0.2073024	-0.2901731	2.63191285	-0.435493	-0.3727197	-0.3874252	0.01870892
A_52_P384209	Syne1	-0.2812798	-0.4625674	2.66249354	-0.3160203	-0.280378	-0.3627784	-0.3420797	-0.3218992	-0.2954909
A_52_P472353	Tenm2	-0.344544	-0.347728	2.66597268	-0.3469158	-0.3037924	-0.3469262	-0.3469696	-0.343677	-0.2854197
A_52_P94201	Syt1	0.57793831	-0.5498832	-0.5142514	-0.3822265	-0.1943726	-0.2042971	-0.4732599	-0.7321904	2.4725429
A_52_P432881	Slc19a3	0.29466142	-0.7967017	-0.355135	0.16118356	1.53938851	0.83708777	-1.282927	-1.2783573	0.88079972
A_52_P454336	Shroom3	-0.2755066	-1.893659	-0.7198664	1.49143731	1.00659308	0.19914333	0.00246769	-0.4175207	0.60691133
A_52_P283724	Wfikkn2	-0.4216514	-0.618857	-0.5349136	0.22327418	2.42459166	-0.3212601	-0.4697189	-0.7969117	0.5154468
A_52_P338651	Pitpnm2	-1.0191745	0.54234211	0.48024264	1.1340092	-0.162269	-0.7669527	-1.6473341	0.09238211	1.34675418
A_51_P309370	Fblim1	-1.9904639	-0.147421	0.03784088	1.57463645	-0.3147811	0.61767836	-0.3045945	-0.3779682	0.905073
A_51_P251468	Dnah10	-0.7718291	-0.7805687	0.07998076	2.34860614	-0.1890278	-0.5562582	-0.6526674	0.68831414	-0.1665498
A_51_P437152	Lrp2bp	-0.6066634	-0.5785269	2.33756924	0.9110197	-0.489772	-0.0741175	-0.5589175	-0.4317978	-0.5087939
A_52_P147803	Syne4	-0.6072908	-0.6920058	-0.6691793	-0.6426134	-0.6502209	-0.5893985	1.89372337	0.87771783	1.0792675
A_52_P583291	Plcb2	0.09966908	-0.743866	-0.7362589	-0.7046234	-0.7214445	0.66948175	2.11146228	-0.6839419	0.70952151
A_51_P495700	Ccdc28b	-0.7978107	-1.306922	-1.1503761	-0.267435	0.73902807	0.45626162	1.04251484	-0.250411	1.53515027
A_52_P57622	Acss3	0.24811887	-0.4423047	-0.5702892	-0.546286	-0.5333823	-0.5448479	2.51309888	-0.3894849	0.26537723
A_52_P112676	Ccdc28b	0.10838836	-0.8175284	-0.8670871	-0.9854171	-0.1289696	0.10283898	1.94705221	-0.5666359	1.20735859
A_52_P160453	Stpg2	-0.0258637	-0.453016	-0.4512427	-0.4370821	-0.3065419	-0.4150808	2.64163836	-0.243744	-0.3090671
A_52_P548399	Pitpnm2	-0.2446792	-0.4027097	-0.4076188	-0.3855699	-0.2845115	-0.3736466	2.66148228	-0.2619904	-0.3007561
A_52_P312204	Nxph1	0.07612692	-0.6250114	-0.5917256	-0.5128661	2.52953157	-0.5483371	0.28579278	-0.2035188	-0.4099923
A_52_P632142	Dlgap2	2.4470652	-0.5589146	-0.5531957	-0.7858062	0.56499918	0.00794023	-0.2198552	-0.4252898	-0.4769431
A_52_P334301	Nim1k	1.7810332	-0.9109212	-0.5619781	-0.8471218	0.7018804	0.85314807	0.62663345	-0.8556326	-0.7870413
A_52_P533129	Mixl1	-0.0102673	-0.4224482	-0.4226503	2.64557103	-0.3780134	-0.3943978	-0.3413137	-0.3389971	-0.3374832
A_52_P67775	Cdh8	-0.3433509	-0.3562839	-0.3558065	-0.2083055	-0.348104	-0.3535584	-0.3538212	-0.3444457	2.66367609
A_51_P153029	Ankrd53	-0.3375938	-0.3501558	-0.3386343	2.66644964	-0.3303874	-0.3389397	-0.3395378	-0.3040815	-0.3271193
A_52_P517247	Iqsec3	-0.2156048	-0.5243183	-0.5029862	2.62344453	-0.3063012	-0.4305564	-0.4373435	0.05713434	-0.2634685
A_52_P605455	Eef2	1.79236724	-0.9880091	-0.9149261	1.35322274	-0.0772514	-0.1632724	-0.9190561	-0.4036257	0.32055071
A_51_P417758	Fut9	2.30111935	-0.6997872	-0.9937995	-0.6741352	0.38479869	-0.5798133	-0.3213455	0.3798353	0.20312733
A_52_P220743	Dhdh	2.28614334	-0.6900758	-0.9710667	-0.7164557	0.40041047	-0.590051	-0.3443904	0.4085425	0.21694321
A_52_P166382	Tunar	2.43790262	-1.0006724	-0.6134725	-0.7724884	0.19524996	-0.1022523	-0.181325	0.01326794	0.02379
A_52_P80555	Nrg3	0.12712915	-0.6881872	-0.6496051	-0.4174739	2.47970701	-0.5260652	-0.5333292	0.40577171	-0.1979473
A_51_P237878	Sntg1	0.55398179	-0.5767756	-0.5454248	-0.4449645	2.49788219	-0.4878276	-0.4782413	-0.1489568	-0.3696735
A_52_P381468	Nxph1	-0.2933691	-0.363578	-0.352355	-0.3298632	-0.323071	-0.3405152	-0.3446687	2.66610231	-0.318682
A_52_P523959	Sntg1	0.07205516	-0.892347	-0.8642712	-0.2008717	-0.1364316	-0.4577405	-0.526214	2.33618412	0.66963675
A_52_P131855	Vcan	1.34876604	-0.9874132	-0.846716	-0.336303	-0.2711175	-0.5953386	-0.5836854	1.85236775	0.41943998
A_52_P271671	Cntn4	1.23261931	-1.0034325	-0.8527343	-0.3142112	-0.2798424	-0.5884657	-0.5802034	1.91407411	0.47219608
A_52_P453864	Syne1	0.19569831	-1.0585854	-0.9316354	-0.2051645	0.23496383	-0.47422	-0.5279027	2.26222046	0.50462542
A_51_P154658	Agbl4	0.03090267	-0.9549477	-0.7957253	-0.37489	0.23281672	-0.5002632	-0.4780859	2.35471344	0.48547932
A_52_P336171	Gabra2	0.59949869	-0.7812586	-0.5783621	-0.4530764	-0.1042853	-0.6902581	-0.7227413	2.30145721	0.42902603
A_51_P468912	Pcdhb1	0.14217271	-0.4738243	-0.456385	-0.3984301	-0.383466	-0.4199532	-0.4229504	-0.2033798	2.61621605
A_52_P404302	Olfml2a	-0.3223185	-0.3365282	-0.3364235	-0.3353172	-0.3335426	-0.3351675	-0.3338621	-0.3334823	2.66664188
A_52_P375435	Maats1	2.53250528	-0.787215	-0.6599963	-0.1249448	-0.3649178	-0.4676179	-0.2927961	0.26184092	-0.0968583
A_52_P334542	Nim1k	2.3692583	-0.9645555	-0.7873084	-0.1460462	-0.3403766	-0.5084699	-0.339977	0.5427162	0.174759
A_52_P604243	Ppp1r14c	1.16416747	-1.8949737	-1.0277733	0.46682565	-0.0834901	-0.3790723	-0.0491841	0.91048009	0.89302028
A_52_P456958	Sybu	2.27260324	-1.0984724	-0.908919	0.13848454	-0.4058752	-0.5691838	-0.1236373	0.4850093	0.20999066
A_52_P22474	Fut9	-0.2912056	-0.3662715	-0.3574737	-0.3228585	-0.3325453	-0.3428756	-0.3310585	-0.3217473	2.66603606
A_52_P412167	Atp6v0a4	-0.2961445	-0.5355042	-0.5168951	2.45570617	-0.4820952	-0.4947398	0.70693511	-0.4042154	-0.433047
A_52_P57776	Nrbp2	2.37181965	-0.9855104	-1.043945	-0.039196	-0.0192558	0.11686733	-0.2897449	0.26676981	-0.3778048
A_52_P275769	Ccdc158	-0.1179262	-0.5729167	-0.5702398	-0.4207192	-0.2479819	2.60659788	-0.470518	0.06592	-0.272216
A_52_P298465	Racgap1	0.83589803	-1.5168486	-1.4386962	0.18042544	0.69205838	1.53097182	-0.273134	-0.0221618	0.0114869
A_52_P499773	Ppp1r14c	0.06378736	-0.4849249	-0.4539236	-0.334686	-0.3952	2.63259876	-0.377108	-0.2925268	-0.3580169
A_52_P1124437	Dock9	-0.3723281	-0.5646641	-0.5533088	1.82980891	-0.5324126	1.69016672	-0.5181669	-0.478647	-0.5004481
A_52_P599789	Qrich2	0.22994487	-0.5370272	-0.5173326	-1.0592954	-1.3249285	1.62025332	-0.0866911	1.32635051	0.34872614
A_51_P221512	Ppp1r2	-1.222413	-0.5197266	-0.9675598	1.25187214	1.71082229	-0.6483441	0.31406648	0.41682662	-0.335544
A_51_P312615	Hist1h1t	-0.4807337	-0.2512189	0.37500865	0.8537885	0.83662138	-0.9626509	0.40879627	1.14944005	-1.9290513
A_52_P290374	Tunar	-0.3732945	-0.3055356	-0.3604733	-0.3485887	-0.3451822	-0.3600812	-0.2334264	2.66429984	-0.3377181
A_51_P365351	Dhdh	-0.9743613	0.12707509	-0.9629832	-0.6620565	-0.0050704	-0.558065	1.27587554	1.92416466	-0.1645788
A_52_P208250	Uggt2	-0.5287321	2.62303374	-0.4787128	-0.4715654	-0.1826305	-0.3276107	-0.4364929	0.03906194	-0.2363513
A_52_P325330	Khdrbs2	-1.9925385	-0.3959266	-0.3543217	-0.0742733	0.9220745	0.28327334	-0.4701411	1.50104496	0.58080836
A_52_P995564	A930018M24Rik	-1.9090548	-0.2822522	0.04478694	-0.1463965	0.31171047	-0.1207233	-0.3998383	1.90333175	0.59843595
A_52_P655409	Alk	-0.7873153	-1.1655867	-0.3230714	0.20251434	0.22983715	-0.4187459	-0.5057597	2.26750134	0.50062619
A_51_P282616	Maats1	-1.3891549	-0.183167	-0.0685993	-0.8194383	1.83755187	0.35712179	-0.8692472	1.04535425	0.08957885
A_51_P456826	Slc19a3	-1.8221341	0.00194751	0.59654348	-0.6343502	1.29929282	0.22765062	-1.0842918	0.66136786	0.7539738
A_51_P240077	Apbb1ip	-2.1264002	0.15933008	0.06616507	0.15549381	1.38381938	0.1947559	-0.8641878	0.82637224	0.20465159
A_51_P177071	Nrg3	-2.3641075	-3.65E-05	-0.2628544	0.0671953	0.88000915	0.55887368	-0.2224552	1.05822158	0.28515401
A_52_P679799	Syt1	-0.6110457	-0.3014739	-0.3383259	-0.3292537	-0.2297583	2.648088	-0.3576538	-0.1938551	-0.2867217
A_52_P359431	Sybu	-1.5415303	-0.311629	-0.2039532	0.15245561	-0.7127631	0.81131292	-0.7430064	1.77176079	0.77735274
A_51_P324886	Scrt1	-0.6618777	-0.5936017	-0.5897502	-0.5936017	-0.5647341	-0.5824725	0.58038918	0.79664649	2.20900219
A_52_P371541	Hif3a	-0.7403384	-0.1475193	-0.6763552	-1.6791209	1.20199545	0.08258835	-0.0675068	0.44558804	1.58066882
A_52_P77867	Stx16	-1.7638639	0.11195579	0.199884	-1.3917964	-0.2954151	0.54683144	1.07356315	0.43776278	1.08107829
A_51_P117627	Ache	-0.8539874	-0.863017	-0.9819351	0.6419708	1.805919	1.16038683	-0.6847963	-0.2665232	0.04198239
A_51_P122170	Rdh7	-1.003232	-1.0031303	-1.0032493	0.83647107	1.26396999	1.56319494	-0.3852806	-0.3006712	0.03192731
A_52_P48976	Il17rc	-1.1056214	-1.0463096	-1.1064084	0.40454345	1.45313342	1.15872847	-0.4038393	0.80724009	-0.1614668
A_51_P267080	Tymp	-0.8351537	-0.6979016	-0.7965072	0.45497697	1.3475057	1.88180754	-0.4018381	-0.4483422	-0.5045475
A_52_P428735	Lrp2bp	-0.9216603	-0.8967255	-0.7712465	0.12794542	2.13284384	0.95755394	0.08090845	-0.2807285	-0.4288908
A_51_P290290	Prkcg	-1.5516662	-0.514331	-0.794034	0.10428185	2.00512115	0.80032945	-0.0330589	0.10159177	-0.1182341
A_51_P411917	Gata6	-1.1917294	-1.1078919	-1.1112533	1.00193632	1.33778519	1.10550091	0.07681519	0.15621489	-0.2673778
A_51_P235726	Icam4	-0.5703629	-0.7484584	-0.9804953	-0.0324177	1.36272662	1.93850451	-0.052997	-0.2190262	-0.6974738
A_51_P480073	Chad	-1.0861917	-1.4407758	-1.1259331	1.06436194	1.06436194	1.06814145	0.16688672	0.15709528	0.1320534
A_52_P644452	Dock9	-0.8622309	-1.0064036	-1.213225	1.75345402	1.25384936	0.30922105	0.00763724	-0.1176593	-0.1246429
A_52_P458279	Prlr	-0.9188524	-1.1851989	-1.2183376	0.45622734	1.28467928	1.16496297	0.32318613	-0.6396932	0.73302638
A_51_P291637	Hspb9	-0.9963057	-1.2397133	-1.4187488	0.20103416	1.27048511	1.04511639	0.31305349	0.04345762	0.78162098
A_51_P350294	Slc9a3	-0.9875316	-0.988235	-0.988188	0.02782983	1.90986547	1.11771266	0.22525753	-0.3495638	0.03285299
A_51_P264673	Prlr	-0.9355287	-1.1357303	-1.1360573	0.6694276	1.21433896	1.34925321	0.571736	-0.6832264	0.08578701
A_52_P602987	Prlr	-0.8874899	-1.1550244	-1.1631943	0.42228607	1.34273964	1.24548468	0.77271251	-0.6270662	0.04955188
A_52_P471583	Foxk1	-1.4441829	-1.3150344	-1.0271058	0.26295682	0.8031427	0.8158197	0.15764955	0.55722898	1.18952527
A_52_P561224	4932438H23Rik	-1.2450413	-1.336791	-1.3493913	0.41384525	0.75551868	0.87212646	0.34103247	0.66530013	0.88340064
A_51_P356413	Foxk1	-1.2643355	-1.328197	-1.2432999	0.2270529	0.83659558	0.53233519	0.27412378	0.82546739	1.14025762
A_51_P182604	Foxk1	-1.3055815	-1.2621255	-1.1488376	0.51156855	1.11018458	1.22817879	0.09205667	0.19808231	0.57647371
A_51_P205334	Tnfrsf8	-1.2896309	-1.1446994	-1.0636223	0.80350196	1.00959343	1.17014519	-0.3081041	-0.0510027	0.87381887
A_51_P101506	Hif3a	-1.198032	-1.3554172	-1.1071125	-0.1319509	1.05648752	1.22684931	0.62116461	0.24028745	0.64772362
A_51_P350048	Gstt2	-0.8873527	-0.8864687	-0.8821927	1.78988161	0.78096945	0.94598239	-0.8594451	0.31664192	-0.3180161
A_52_P342443	Fblim1	-1.8271402	-0.7318714	-0.5525672	1.521953	0.22439496	1.10752411	-0.1408993	0.45227825	-0.0536723
A_52_P192084	Agbl4	-0.8341049	-0.4639358	-0.5013937	2.21983426	-0.3065486	1.13037726	-0.5182069	-0.3075312	-0.4184905
A_52_P617076	Galntl6	-1.1026415	-0.8651644	-0.8264323	0.27281953	0.0852479	0.65354958	-0.0419156	2.17275104	-0.3482143
A_52_P299535	Pkdcc	-1.1599533	-1.2062798	-1.2140572	1.61332725	0.52622288	0.27516017	0.12105491	0.84617873	0.19834637
A_52_P488908	Hes7	-1.1073655	-1.1201673	-1.3087517	1.33263188	1.27559377	0.27066653	-0.1270144	0.33720939	0.44719732
A_52_P570861	Klhl5	0.04987968	-0.3224969	-1.6687487	1.51430367	0.61700061	0.6984044	-1.3537352	0.28889833	0.1764941
A_51_P243750	Racgap1	-0.9274419	-1.4209057	-1.4007307	0.93465535	0.94175649	0.52394514	0.9920437	0.37336125	-0.0166837
A_51_P474701	Fbp1	-1.2357628	-1.235785	-1.2357857	0.41305678	0.61130113	0.39971284	1.52707039	0.597899	0.1582934
A_51_P274488	Pcdh8	-0.3926871	-0.5041995	-0.5038081	-0.3598048	-0.0828683	-0.1813817	2.63734224	-0.1937749	-0.4188177
A_52_P288284	Hif3a	-0.6313972	-0.7865505	-0.70576	-0.3571177	2.11076597	0.6914203	0.8254267	-0.3675291	-0.7792584
A_51_P404193	Sp5	-0.860006	-0.8672573	-0.8672128	0.39226355	0.16079545	0.91464503	2.03739817	-0.160115	-0.7505112
A_51_P231184	Anpep	-1.0878116	-1.1694949	-1.1752755	1.22132515	0.43586067	0.8066542	1.23503441	-0.4286193	0.16232682
A_52_P189410	Atp8a2	-1.2227349	-1.2101641	-1.2034253	0.83415014	0.58532831	0.62725865	1.44575812	0.244774	-0.100945
A_51_P213045	Smoc2	-0.4914628	-1.1508604	-1.0969393	1.51395955	-0.1024674	0.92293316	1.2527446	-0.1523956	-0.6955118
A_52_P154564	Scrt1	-1.062205	-1.427476	-1.3489483	0.85710613	1.03954741	0.32174353	0.88287986	0.21994542	0.5174069
A_51_P212741	Scn2b	-1.0195327	-1.0887784	-1.098543	0.31691412	0.6567893	0.51131874	1.85216692	-0.475075	0.34474005
A_52_P244702	Tcf7	-1.297838	-1.11253	-1.1686427	0.71212908	0.93775343	0.45846638	1.44270355	-0.1157948	0.14375313
A_52_P244711	Tcf7	-1.2396382	-1.0872441	-1.145801	0.92074416	1.43149538	0.19491856	1.00166324	-0.1546188	0.07848075
A_51_P259876	Dock9	-1.4553484	-0.8022228	-0.8045907	1.83362489	0.7085443	0.45492801	0.49873757	-0.514396	0.08072317
A_51_P175424	Car14	-1.0809272	-1.2345441	-0.6015629	-0.4775935	1.23299929	1.71100018	-0.0029912	0.51561824	-0.0619988
A_51_P246317	Mt2	-0.8900606	-1.33887	-1.3810917	-0.3688185	0.92575764	1.01996823	0.85231353	0.74982284	0.43097867
A_52_P681592	Cybrd1	-0.7276905	-0.9361021	-0.9320305	-0.6790602	1.56655211	1.44162423	-0.5888153	0.52611542	0.32940689
A_51_P509530	Cybrd1	-0.6529069	-0.8220609	-0.8229873	-0.5369096	1.72726736	1.59125182	-0.5363656	0.279077	-0.2263659
A_52_P124734	Cybrd1	-0.5163888	-0.7921478	-0.7908245	-0.5544199	1.70611315	1.6857137	-0.409389	0.1511077	-0.4797646
A_51_P225781	Insc	-1.0378771	-1.0260903	-1.0355035	-0.2902081	0.54173641	0.65206496	-0.2260472	1.92189037	0.50003452
A_52_P233411	Insc	-1.0408073	-1.060973	-1.0418668	-0.2644355	0.53869392	0.6102938	-0.1426068	1.93179955	0.46990212
A_52_P164615	Ppp1r2	-1.5493987	-0.7328988	-0.7159647	-0.2467056	1.98541442	0.3442789	0.46086684	0.4646004	-0.0101928
A_52_P138540	Ppp1r2	-1.7460403	-0.7546202	-0.8085163	-0.2841926	1.34733953	0.67327311	0.67565675	0.95840332	-0.0613034
A_51_P473043	Ppp1r2	-1.6706995	-0.8788071	-0.7199837	-0.3515657	1.52325413	0.61525253	0.77072059	0.70825444	0.0035743
A_51_P461864	Znhit1	-1.243957	-0.8552244	-0.9629734	-0.8120081	1.28784229	0.92417465	1.23735522	0.22941546	0.19537522
A_52_P337326	Kcnc3	-0.9966579	-1.0699552	-0.8842937	-0.3396093	1.86320936	0.63009282	0.99972739	0.09372073	-0.2962343
A_52_P24106	Znhit1	-1.1282389	-0.7522294	-0.795797	-1.1715136	1.36631288	1.1602515	0.92477185	0.26421493	0.13222765
A_52_P265965	Trim36	-1.2683538	-1.2005847	-1.2044902	-0.0402445	0.83488017	1.34299243	0.8859869	0.34366143	0.30615226
A_51_P104392	Rpp25	-1.2711344	-1.0777194	-1.0249269	0.07689526	0.71075847	1.04109595	1.08912149	1.03019523	-0.5742857
A_52_P284348	Smim20	-1.4866498	-1.2002759	-1.2371192	0.42315279	0.5774611	0.69520197	1.0649729	0.50361954	0.65963672
A_52_P553171	Suds3	-0.8086636	-1.3498457	-1.2702486	-0.5835495	0.39907986	1.10867098	1.01060987	0.59826039	0.89568642
A_52_P288860	1110051M20Rik	-0.9387802	-0.8887696	-0.7074798	-0.9072823	-0.4451551	1.47035477	1.44600921	0.68867948	0.28242363
A_51_P299805	Slc46a3	-0.9455669	-0.928445	-0.8706375	-0.3531521	-0.3172678	-0.1304443	1.65359371	0.34619345	1.54572647
A_52_P183368	Tbx3	-0.5671667	-0.4902359	-0.4810827	-0.4530243	-0.4689392	-0.3401506	2.55958871	0.32687298	-0.0858622
A_51_P490204	Pop5	-1.4912527	-1.1889324	-1.1233587	0.09038143	0.37843596	0.79202796	1.08901254	0.92612905	0.52755684
A_52_P481279	Drc1	-1.2021309	-1.1217077	-1.0840296	-0.5679531	0.20328319	1.09688415	1.16145834	0.92900423	0.58519141
A_51_P490198	Pop5	-1.2730786	-1.3010746	-1.1808171	0.00808545	0.39827672	0.69140585	1.16579796	1.04917111	0.44223317
A_51_P255657	2210011C24Rik	-1.1276625	-1.0729358	-1.0806962	-0.1971903	0.16009564	0.3212432	1.28745655	1.60401311	0.10567631
A_51_P509486	Sybu	-0.3512677	-0.3524604	-0.3421807	-0.3476457	-0.3175275	-0.3198947	2.66630556	-0.3059502	-0.3293787
A_51_P347529	Nek5	-0.9583545	-0.9701697	-0.9113012	-0.7021605	0.48456328	-0.0153997	1.91390045	0.93104621	0.22787573
A_51_P353776	Gjb3	-1.0592667	-1.1245756	-1.1347298	0.53136191	-0.0448676	0.05066198	0.14120467	0.80365099	1.83656014
A_52_P383862	Cmc1	-1.1525507	-1.1787299	-1.1942754	0.71493605	-0.0777033	-0.0959282	0.58541316	0.96528844	1.43354986
A_51_P260356	Plcb2	-0.2317963	-1.6128186	-1.2971474	0.31835582	-0.1106941	0.07977453	0.25726158	1.07052778	1.52653675
A_52_P161297	Tcea3	-0.8596904	-1.1500295	-1.1444075	0.60482095	-0.4713009	-0.2379265	0.5438556	1.31469905	1.39997915
A_51_P351273	Tcea3	-0.7509008	-1.139955	-1.1484109	0.62210612	-0.5273518	-0.370161	0.64505754	1.20037518	1.46924066
A_52_P295104	Smim5	-1.1254017	-1.1281823	-1.1279327	0.08162326	-0.1614719	-0.0415789	0.96990756	1.21591714	1.31711958
A_52_P143982	Il17rc	-0.5188378	-1.2441007	-1.3271324	-0.4269976	-0.0659118	0.27989697	0.59432702	1.09474156	1.61401463
A_52_P143976	Il17rc	-0.63274	-1.3303893	-1.3625023	-0.2529738	0.08182051	0.31528584	0.58456655	1.08698417	1.50994827
A_52_P214927	Zmynd8	-0.9026379	-1.2187921	-1.1729652	-0.2951861	-0.026329	0.33819562	0.51041901	1.25666641	1.51062929
A_52_P176245	Gata6	-0.7598766	-0.7260034	-0.7380639	-0.300434	-0.2467895	-0.3130618	0.07921998	0.61360672	2.3914025
A_51_P476538	Inafm2	-1.4874125	-1.0544085	-1.2080557	0.19173285	0.46233152	0.17623321	1.01119148	1.16794429	0.74044328

Supplementary Table 1: Genes associated with iISC-high DMRs

Probe_ID	Gene_symbol	MEF_1	MEF_2	MEF_3	ISC_1	ISC_2	ISC_3	iISC_3	iISC_1	iISC_2
A_51_P220062	Mmp15	-1.2541573	-1.1670733	-1.1736832	0.61979786	-0.1197374	0.26025794	0.51322986	1.52365844	0.7977071
A_52_P262209	Atp2a3	-1.1876525	-1.1174535	-1.1383497	0.33411562	-0.0980954	0.04706542	0.53770931	1.62210522	1.00055555
A_52_P623058	R3hdm4	-1.4906249	-0.8822242	-0.8797727	0.76217881	-0.0246079	-0.1866106	0.06912414	1.58033982	1.05219766
A_51_P364168	Lrp5	-2.1651936	-0.1823475	-0.5022416	0.27427209	0.45544565	-0.1396061	-0.0633129	1.05623446	1.26674939
A_51_P233367	Fzd10	-1.9622325	-0.3679301	-0.9382322	0.45009581	0.63982108	0.08567885	-0.1388963	1.16668413	1.06501128
A_51_P287691	Kremen2	-0.8284142	-0.8243501	-0.8303019	-0.5828114	0.17852896	-0.0668051	-0.327472	1.4446006	1.83702528
A_51_P496253	Slc6a4	-1.2677512	-1.2675035	-1.2663072	0.76340771	0.48809433	0.38481822	0.24092564	0.57225135	1.35206461
A_52_P590175	Gata6	-0.9329526	-0.9065489	-0.8884249	0.00505309	-0.027515	0.00366492	-0.0318353	0.47920642	2.29935236
A_51_P195129	Shroom3	-1.3170618	-1.2809403	-1.2799486	1.09298389	0.30437201	0.53983887	0.32853124	0.8027866	0.80943821
A_52_P548470	Shank2	-1.1870897	-1.089533	-1.0428561	0.98222278	0.58891138	0.68238506	-0.6155275	0.22984855	1.45163865
A_51_P117618	Ethe1	-0.4778019	-0.7765484	-0.7619164	-0.3724784	-0.2837466	-0.2166619	-0.3766441	2.2838169	0.98198077
A_52_P627761	Ethe1	-0.4084638	-0.7730795	-0.7286986	-0.3601932	-0.3919663	-0.2134431	-0.4293759	2.25633385	1.04888656
A_52_P1171706	Samd4	-0.6514361	-0.8560347	-0.7376961	-0.2676267	0.36717577	-0.3242894	-0.453182	2.3302894	0.59279987
A_52_P425510	Dnah7a	-0.4283449	-0.5425587	-0.5022032	-0.4401515	-0.1858762	-0.40625	-0.4433315	0.39440113	2.554315
A_52_P507382	Unc93b1	-0.1606074	-0.5602822	-0.6514888	0.68277044	-0.1097835	-0.0697746	-1.780607	1.51402268	1.13575045
A_51_P301975	Unc93b1	-0.0631189	-0.2290648	-0.3987578	0.87483744	-0.0002195	0.03933615	-2.2344871	1.03491105	0.9765634
A_52_P263076	Prr36	-0.590462	-0.8372667	-0.8494508	1.8523989	-0.1779756	-0.141715	-0.8550037	0.1933378	1.40613709
A_52_P239386	Ppp1r14c	-1.1255816	-0.7972862	-0.7242332	0.72368775	-0.512252	-0.3155767	0.43707919	2.1106168	0.20354586
A_52_P29765	Cmc1	-1.5034	-0.8884703	-0.8892922	0.70640343	-0.5733701	0.12959426	1.06175051	1.34798525	0.6087991
A_52_P439420	Wscd1	-1.2603137	-0.5673385	-0.5219004	1.31818595	-0.6052288	-0.3657742	0.30277599	1.86065463	-0.161061
A_51_P290826	Calca	-0.8231687	-0.7128438	-0.719133	-0.3977125	-0.7176408	-0.5857756	1.48422465	1.13033545	1.34171425
A_52_P646762	Smoc2	-0.3554249	-1.4439116	-1.4377752	1.24410786	-0.1969547	0.70737483	1.19544368	0.43019688	-0.1430568
A_52_P519904	Kcnc3	-0.4163876	-0.4371713	-0.4255509	2.65616272	-0.335822	-0.2924426	-0.2425219	-0.1696472	-0.3366193
A_52_P263774	Cbx7	-1.246417	-1.2420134	-1.246393	1.44988323	0.34510522	0.31784645	0.76400973	0.62429931	0.23367945
A_51_P211770	Dpy19l2	-0.4192384	-0.5253388	-0.497145	2.58257061	-0.3883809	-0.4614939	0.28560826	-0.1887358	-0.3878461
A_51_P305628	Atp8a2	-1.1680257	-1.3081579	-1.3107171	1.0566282	0.33731334	0.23226273	1.19900093	0.58629324	0.37540234
A_51_P169156	Rps10	-0.9104173	-1.2963156	-1.232067	1.22205369	-0.3405568	-0.0983512	0.90197828	0.84223081	0.91144522
A_51_P389945	4932438H23Rik	-1.259078	-1.2887617	-1.2858323	1.10431802	0.39562383	0.58852058	0.70395842	0.10807339	0.93317779
A_51_P317433	Mesp2	-1.2260489	-1.2268164	-1.2024707	0.55130541	0.02042546	0.22919129	1.10730853	0.40633467	1.34077072
A_51_P453657	Syt8	-0.3549505	-0.4599134	-0.4622903	-0.1216326	-0.4375806	-0.3955688	-0.1371718	-0.2750486	2.64415653
A_51_P237255	Kcnip2	-1.0401943	-0.9984453	-0.8775955	1.48712288	0.5053553	0.02653205	0.6290067	-0.9269486	1.19516673
A_52_P289559	Klhl23	0.52778854	-0.3138948	-0.2157554	0.28762102	1.08321508	1.72386113	-1.0918463	-1.2784097	-0.7225796
A_52_P517508	Klhl23	1.3280053	0.06365448	0.06873985	-0.3071004	0.60277346	1.49134038	-1.0588802	-1.2163665	-0.9721665
A_51_P459787	Klhl23	-0.0224861	-0.3576141	-0.4429829	-0.2713144	0.8032511	2.350624	-0.6946719	-0.5896687	-0.7751371
A_51_P262201	Atcay	-0.2986588	-0.2130294	0.01073301	-0.5810207	1.15308419	2.13668471	-0.6633056	-0.7714068	-0.7730807
A_51_P209697	Atcay	-0.3452068	0.12946317	0.11013367	-0.5642125	1.63340763	1.55419576	-0.3816846	-0.9609865	-1.17511
A_51_P124741	Ppp1r14c	0.35953116	0.74117416	0.63845669	-0.9890813	0.85761149	1.44205461	-1.0367217	-0.9076602	-1.1053649
A_51_P275679	Rassf5	0.01511514	0.08367966	0.01195463	1.84712195	0.1432302	0.79964547	-0.1573095	-1.6294473	-1.1139903
A_51_P376299	Mchr1	-2.2749136	0.29228889	0.35742383	0.27090661	1.17148592	0.8701625	-0.4991571	-0.3603354	0.17213835
A_51_P164504	Apoc1	-2.0084849	0.33762592	0.12287235	0.18887402	1.51216872	0.93134199	-0.0471556	-0.287305	-0.7499375
A_51_P417600	Npbwr1	-1.7696179	0.01824104	-0.522428	1.30019947	1.59037115	0.20884898	-0.5391967	-0.1464425	-0.1399755
A_52_P82701	Slc9a3	-0.5732439	-0.5376855	-0.5274888	0.37736203	2.06293288	1.13051255	-0.5539437	-0.9816085	-0.3968371
A_52_P222193	Nkx1-2	-1.7386513	-0.3205788	0.01026209	0.52792529	1.99696737	0.24048198	0.18638682	-0.6832827	-0.2195107
A_51_P232913	Cpt1b	-0.9714877	-0.7071912	-0.4706577	0.71629696	1.07590845	1.54327431	0.62203141	-0.543153	-1.2650215
A_52_P625508	Cpt1b	-0.8878445	-0.6555319	-0.6004419	0.57668348	1.39643156	1.60645514	0.24767226	-0.6418414	-1.0415828
A_52_P430211	Arhgap17	-0.6239693	-0.7149341	0.06966216	0.86112808	1.01988848	1.1734775	0.70851778	-0.8441001	-1.6496705
A_51_P132170	Ccdc141	0.09820132	-0.623486	-0.7016766	1.18584812	0.53289755	1.59162896	0.17136004	-0.7308974	-1.523876
A_52_P628127	Fam229a	-0.5884627	-0.5987959	-0.5630831	1.35473752	2.08823442	-0.2579028	-0.3506042	-0.4922769	-0.5918464
A_52_P579876	Suds3	-0.7289295	-1.0230692	-0.91923	0.37525479	0.78929935	1.38008674	1.18180854	0.1634546	-1.2186753
A_51_P417257	Ppp1r2	0.32752567	-0.854208	-1.092129	1.02146465	0.83622627	0.42554377	1.35968044	-0.7388038	-1.2852999
A_52_P389431	Tbx3	-0.52335	-0.3479056	-0.4689867	0.05276327	-0.13122	0.07105147	2.57126478	-0.6165482	-0.607069
A_52_P273812	Ifitm5	-0.3326942	-0.7907289	-0.1167776	1.91852573	0.62765212	-0.4441016	1.01184836	-1.2524988	-0.6212252
A_51_P317376	Synpo	-1.0006349	1.71293113	0.659521	-0.9662328	0.86622361	0.27276502	0.33198307	-0.9253429	-0.9512133
A_52_P409477	Csrnp1	-1.1328321	0.56542243	0.47372454	-0.8005038	1.78668237	0.17279501	0.74199093	-1.1546334	-0.652646
A_52_P638439	Ppp1r2	-0.6535029	0.03871617	0.04346109	-0.8426566	2.29064064	0.26695648	0.3471765	-0.3520672	-1.1387243
A_51_P305777	Syne1	-0.6498627	0.32729598	-0.6336016	-0.4220314	-0.3455765	2.51130904	-0.5859596	0.12419513	-0.3257684
A_51_P423743	Cldn6	-1.0038175	0.07271536	-0.7392315	1.32224455	-0.3778203	1.97222642	-0.6735403	-0.141215	-0.4315617
A_51_P359078	Rangrf	-0.4910045	-0.4169737	-0.4306786	-0.1510794	1.21735624	1.90336813	-0.9082614	0.40865815	-1.1313849
A_51_P488063	Crocc	1.09650344	-0.3119183	-0.1013415	0.50145371	-0.5845771	0.96428152	1.1774707	-1.2667188	-1.4751536
A_52_P126728	Actrt3	0.72913107	-0.3528008	-0.6346884	0.6431284	-1.0092337	1.29129133	1.34685023	-1.0041302	-1.0095479
A_51_P298837	Spock1	-0.3768432	1.91316151	-0.5413582	-0.5137895	-0.5300379	1.59947806	-0.5150139	-0.5204348	-0.515162
A_51_P215353	Tmem132c	-0.0630411	0.80865976	0.73145102	-0.9105975	-0.8797913	-0.9130491	0.20885427	1.88877654	-0.8712626
A_52_P526839	Ccdc158	-0.2940431	0.73844271	-0.1204801	-0.836093	-0.8536981	-1.0382984	2.09667042	0.6034748	-0.2959752
A_52_P254095	Cd200	-0.8548845	0.50121447	0.57076609	-0.6110511	-0.7977413	-1.036634	1.43933816	1.44921152	-0.6602193
A_52_P1044655	Vcan	0.3929156	0.88312287	1.47485699	-0.8177201	-0.8115423	-0.821333	1.28027784	-0.7756494	-0.8049286
A_52_P250400	Pcdh20	0.4791289	0.85310972	0.94702821	-1.3370319	-1.0432701	-1.2506081	1.09683936	-0.357454	0.61225783
A_51_P282630	Pacsin3	0.65789336	0.64803726	0.63192371	-0.9299679	-1.2882304	-1.287195	1.50637481	-0.3538732	0.41503749
A_52_P228059	Nudt4	-1.0323134	-0.1429996	-0.1617876	-1.1290158	-0.5957537	-0.6942446	1.4462209	1.06337637	1.24651753
A_51_P456465	Cldn10	-0.3523875	-0.359886	-0.3514935	-0.4058475	-0.3908868	-0.384941	-0.1329473	2.65736197	-0.2789723
A_52_P87843	Aldh1a3	-0.4817504	0.09510565	0.17275658	-0.6881379	-0.719196	-0.705106	-0.3029771	0.15433847	2.47496668
A_51_P377557	Cpsf4l	-1.6896884	-0.1074584	-0.2131848	0.15003568	-0.9296142	-0.2866476	0.32607184	1.03598736	1.71449853
A_52_P305246	Nudt4	-0.7031662	0.22306131	0.20745487	-0.5658844	-1.1054348	-1.4236543	1.14226811	0.93536342	1.28999202
A_52_P365466	Plcb2	-0.7132029	-0.6857141	-0.6132359	-0.0438693	-0.7465884	-0.7808881	2.05129238	0.98329673	0.54890954
A_52_P191086	Kcnj6	-0.9296321	-0.6980056	-0.3064739	1.10105243	-1.0204924	-1.0761963	1.31450614	1.04245801	0.57278375
A_52_P49014	Shh	-0.8648415	-0.4149051	-0.478433	0.1496255	-0.8594673	-0.8445746	1.92249627	1.2927313	0.09736841
A_51_P214503	Frem2	-0.406935	-0.3145888	-0.3168944	-0.0711964	-0.4109091	-0.4153761	-0.307314	-0.4077911	2.65100502
A_51_P376656	Synpr	-0.587468	1.35744502	1.41159636	0.25900559	-0.7688679	-0.6539016	-0.2180894	0.6879019	-1.4876219
A_51_P331798	Pip5kl1	-0.7587696	0.88824729	0.55409879	0.96707264	-0.8214132	-1.5621104	0.89604971	0.77336961	-0.9365448
A_52_P306162	Syne1	1.89760359	-0.5883323	-0.234254	-0.3455407	-0.6919791	-0.710815	-0.1767804	1.54353339	-0.6934354
A_52_P380114	Cep112	0.31038315	-0.2170734	-0.4400885	-0.3848334	-0.7730425	-0.7183835	2.44358925	0.340084	-0.5606351
A_51_P161737	Gramd1b	-0.5445578	0.01276022	0.0894716	-1.2132577	0.01813657	-0.0515843	0.21701862	-0.8592673	2.33128017
A_52_P5696	Gramd1b	0.34158363	0.09896516	-0.0658057	-1.24524	-0.3330081	-0.0980227	0.67643134	-1.3525552	1.97765156
A_51_P332228	Nol4	-0.0708455	-0.2501511	-0.2081417	-0.49912	-0.1127412	-0.5012184	-0.502052	2.62536194	-0.4810922
A_52_P651226	Cntn4	0.19802544	-0.6611445	2.43378044	-0.5394456	0.05145772	-0.6912671	-0.7808598	0.32570201	-0.3362485
A_51_P470304	Nkx1-2	0.19922156	0.15598765	-0.6824852	1.53526984	0.18901549	-0.2001605	-1.6579511	1.27511766	-0.8140155
A_52_P167535	Efcab12	-0.3867902	1.05200007	0.81370892	-0.9270521	-0.2423016	0.75492279	-1.6214371	1.22962352	-0.6726744
A_52_P83097	Kcnq5	-0.2101118	1.94960535	-0.547174	-0.9849926	-0.296631	0.13959665	-0.7874112	1.33967122	-0.6025526
A_51_P338443	Angptl4	1.85343349	-0.3114727	-0.2217381	-0.8191138	0.48233724	0.81312188	-1.0681698	0.48655607	-1.2149543
A_51_P123676	Synpo	1.14885397	1.37889689	1.4570385	-0.7296755	-0.6223579	-0.6655068	-0.6158066	-0.6950276	-0.6564149
A_52_P125467	Reep2	0.72530613	1.5609267	1.58718801	-0.6993015	-0.5593706	-0.6468089	-0.587643	-0.6955947	-0.684702
A_51_P203138	Foxh1	-0.2938382	1.52823656	1.53275928	-1.120396	-0.1870021	-0.3791429	0.57603958	-1.041305	-0.6153513
A_51_P455632	Stx16	-0.0567061	1.09195728	1.35956279	-1.7854698	0.82670354	0.07646418	-0.2528575	-0.9626283	-0.2970259
A_51_P298873	Syt1	-0.3358077	0.11022836	2.62911454	-0.402981	-0.3992639	-0.3992944	-0.4007388	-0.4009414	-0.4003157
A_51_P388661	Msx2	0.73053464	1.59478183	1.55396588	-0.6493289	-0.6422753	-0.649473	-0.6492332	-0.6484299	-0.6450421
A_52_P949440	Tgfb1i1	0.82562365	1.52183648	1.56541882	-0.6568175	-0.6504614	-0.6506296	-0.6513617	-0.6553512	-0.6482576
A_51_P173459	Nim1k	1.59921491	1.11861397	1.2461684	-0.7171643	-0.6459101	-0.6719871	-0.6614626	-0.7157912	-0.6506819
A_51_P227662	Dnah7a	-0.2019434	-0.1467599	-0.1760271	-0.4545279	2.63751297	-0.4062351	-0.4259588	-0.2457454	-0.5803154
A_51_P196390	Alk	0.12470507	0.62867195	0.90516239	-0.7359576	2.01694331	-0.7349543	-0.7364193	-0.7331473	-0.7350012
A_51_P227502	Mfap2	2.38519122	0.46351608	0.4890859	-0.5563419	-0.5561325	-0.5561619	-0.5564296	-0.5562973	-0.5564301
A_52_P342159	Nfatc4	1.72170701	1.09299998	1.12177756	-0.6576094	-0.6522771	-0.6529874	-0.6574675	-0.6568177	-0.6593253
A_51_P478138	Cacnb1	1.42349431	1.20095387	1.35885482	-0.6295549	-0.5850887	-0.5582048	-0.7988744	-0.6972181	-0.7143621
A_52_P508962	Kcnab2	1.50938407	1.21991049	1.24493985	-0.6479539	-0.5107714	-0.6370577	-0.8204543	-0.6481331	-0.709864
A_52_P77695	Fam189b	1.36434311	1.35590415	1.25602494	-0.628275	-0.5931097	-0.5363532	-0.8833061	-0.5763373	-0.758891
A_52_P222624	Hspb2	1.36881138	1.3524554	1.27749009	-0.6696991	-0.6694827	-0.6527452	-0.6700564	-0.6675654	-0.6692081
A_52_P209484	Tmem88	1.15546936	1.42551388	1.40726125	-0.6646547	-0.643435	-0.6476217	-0.6671594	-0.6840771	-0.6812966
A_51_P354706	Lefty1	0.47622596	1.49363778	1.54589695	-0.4790787	0.08227491	-0.1852542	-0.8986981	-1.0597685	-0.9752361
A_51_P126626	Zfp503	0.55973515	1.5875948	1.53515228	-0.7209506	-0.3904895	-0.1192984	-0.6294529	-1.0934638	-0.7288271
A_51_P240136	Gng8	1.59141066	1.22774598	1.15325548	-0.6615742	-0.6578024	-0.6598966	-0.66257	-0.6652852	-0.6652836
A_52_P223724	Khdrbs2	-0.3322671	2.66666555	-0.3323574	-0.3336845	-0.3326888	-0.3326965	-0.3337608	-0.3349405	-0.3342699
A_52_P328776	Pitpnm2	1.00936665	1.59703304	1.31042336	-0.6602296	-0.501082	-0.3899165	-0.8401839	-0.813131	-0.71228
A_51_P112529	Pitpnm2	0.89224965	1.47863882	1.5450134	-0.5137185	-0.6497503	-0.4981443	-0.7153568	-0.7823178	-0.7566142
A_52_P495565	Efnb3	0.37979361	1.50583547	1.670905	-0.3513363	-0.507441	-0.2842112	-0.2930597	-1.0449276	-1.0755583
A_51_P222359	Emilin1	2.22483891	0.65100986	0.70198471	-0.5940902	-0.5963701	-0.5984683	-0.5966047	-0.5972729	-0.5986273
A_51_P173858	Apba2	1.34128575	1.15888705	1.36392738	-0.443831	-0.5117315	-0.2354151	-0.6309148	-1.0207115	-1.0214963
A_51_P215106	Fstl1	1.50458599	1.18372	1.29845418	-0.6642132	-0.66391	-0.6636201	-0.6649415	-0.6650309	-0.6650445
A_52_P304128	Mmp14	1.94393281	0.93962162	0.94206358	-0.5808176	-0.5947833	-0.6112327	-0.6912669	-0.6855768	-0.6619408
A_51_P216303	Mmp14	1.63403956	1.16367134	1.16012017	-0.5823842	-0.6133519	-0.6203391	-0.7287496	-0.7196004	-0.6934059
A_52_P384250	Shc1	1.4810184	1.26398734	1.24432664	-0.6276795	-0.6156073	-0.6764897	-0.7256531	-0.6855482	-0.6583546
A_52_P424308	Sybu	2.08427365	0.81823327	0.73096118	-0.3860337	-0.4011104	-0.4621001	-0.757649	-0.6875091	-0.9390658
A_51_P235548	Mtmr9	1.40668717	1.29210016	1.28283594	-0.5344759	-0.5541576	-0.6015028	-0.7866905	-0.7384084	-0.766388
A_51_P395405	Klhl5	1.98758327	0.9014809	0.89270297	-0.5721695	-0.5913174	-0.4921235	-0.7830982	-0.7242581	-0.6188004
A_51_P130332	Hspa12b	2.03405444	0.86188185	0.86969353	-0.6231657	-0.6300334	-0.6218152	-0.6313359	-0.6313798	-0.6278999
A_52_P318631	Eef2	2.00087571	0.43316426	0.33868088	0.72944687	-0.4117783	-0.4476416	-1.2999371	-0.9881991	-0.3546117
A_52_P444637	Rcbtb2	1.40949513	1.11308723	1.21587357	0.1802278	-0.7576949	-0.9218322	-0.8185089	-0.9936759	-0.4269719
A_51_P505384	Cntn4	2.13525933	0.29720767	0.49686792	-0.4296433	0.10976484	-0.8714733	-0.7228946	-1.2530546	0.23796596
A_52_P335984	Syne1	2.11545541	0.71872907	0.85174681	-0.6079085	-0.6028808	-0.6470322	-0.6898894	-0.6300011	-0.5082193
A_52_P148049	Mtmr9	1.56567399	1.05706875	1.26258226	-0.6118258	-0.4013918	-0.7061141	-1.0629731	-0.7316138	-0.3714064
A_52_P430229	Rai14	1.00012436	1.14160854	1.52716595	0.09822991	-0.379219	-0.6365947	-0.5107971	-1.2519069	-0.988611
A_52_P86892	Nrn1l	1.0950738	1.15264746	1.42540822	0.20579785	-0.4031553	-0.8366754	-0.8321538	-1.2279054	-0.5790375
A_51_P264934	Matn4	2.56078578	0.15761125	0.16849903	-0.4798279	-0.471973	-0.4844398	-0.483004	-0.4841904	-0.4834609
A_52_P622614	Nfatc4	2.11255679	0.73436813	0.84841816	-0.5970028	-0.5892039	-0.6303535	-0.6102662	-0.6406871	-0.6278295
A_52_P630632	Rcbtb2	1.75018279	1.01253775	1.0492285	-0.3319695	-0.6682816	-0.6874603	-0.2967935	-0.8053735	-1.0220707
A_51_P138185	Rcbtb2	1.72194498	1.01873328	1.013402	-0.2300659	-0.6773545	-0.6880172	-0.20533	-0.8441388	-1.1091738
A_51_P366344	Tgfb1i1	1.09706205	1.41971635	1.46316895	-0.6631085	-0.6633858	-0.6637143	-0.6630986	-0.663465	-0.6634653
A_51_P489452	Cdo1	0.57867472	1.37946802	1.50060239	-0.0698448	-0.7654749	-0.5951148	0.21137699	-1.0403965	-1.1992911
A_51_P468876	Dixdc1	0.99757308	0.99852554	1.05362158	1.02417811	-1.1491102	-1.0166774	-0.7011939	-0.2328837	-0.9740331
A_52_P1042732	Akap5	-0.3418894	1.59293466	1.88109375	-0.2146688	-0.5632059	-0.7072258	-0.4248525	-0.5206975	-0.7014796
A_52_P470211	Rai14	0.6610312	0.9581606	1.96489742	-0.1408214	-0.9535592	-0.8326871	-0.1942415	-0.5669545	-0.8958255
A_51_P462160	Fam19a5	0.96798713	1.4562256	1.52595954	-0.6825927	-0.6831487	-0.683212	-0.5546156	-0.663731	-0.6828722
A_52_P47660	Tenm2	0.4749502	1.85263542	1.3721984	-0.4838253	-0.7336572	-0.7389508	-0.289304	-0.7234847	-0.730562
A_52_P627967	Rprm	1.35622733	1.36352661	1.27905713	-0.6699845	-0.6700074	-0.6700096	-0.6488179	-0.6699978	-0.6699938
A_52_P523503	Tmem154	1.70731812	1.09416732	1.13973368	-0.6558588	-0.6574558	-0.6575385	-0.656739	-0.6567317	-0.6568953
A_52_P529374	Enox1	2.14282671	0.83208192	0.68938777	-0.5795323	-0.6261281	-0.626141	-0.580442	-0.6260202	-0.6260327
A_51_P278653	Rprm	1.50727657	1.24041258	1.24041258	-0.6637905	-0.6666669	-0.6667519	-0.6589958	-0.6656451	-0.6662516
A_51_P490148	Nphp4	1.59697358	1.14625678	1.22727239	-0.6676517	-0.670779	-0.6708684	-0.6200797	-0.6705584	-0.6705657
A_52_P103929	Syt1	1.56321028	1.1864923	1.19616225	-0.5237995	-0.7554952	-0.7586481	-0.3997212	-0.7521338	-0.7560671
A_51_P217498	Slc2a4	0.58103701	1.58343826	1.65015971	-0.6402101	-0.6681074	-0.6167452	-0.5803715	-0.6259063	-0.6832944
A_51_P328652	Acot7	1.14216175	1.32140213	1.47822635	-0.6230465	-0.7996045	-0.7440824	-0.4257804	-0.4608223	-0.8884542
A_51_P219385	Six5	2.23426472	0.56393031	0.70936343	-0.5360513	-0.8125273	-0.6359543	-0.3727714	-0.4440604	-0.7061937
A_51_P142107	Fzd7	2.1369173	0.746925	0.79006316	-0.6048818	-0.6291229	-0.6096243	-0.6031996	-0.5865225	-0.6405543

Supplementary Table 1: Genes associated with iISC-high DMRs

Probe_ID	Gene_symbol	MEF_1	MEF_2	MEF_3	ISC_1	ISC_2	ISC_3	iISC_3	iISC_1	iISC_2
A_52_P653678	Cdh8	1.84806616	1.11134484	0.38469044	0.49848327	-0.7686052	-0.7684962	-0.7682955	-0.7682006	-0.7689872
A_51_P267053	Thbs3	1.71463847	1.00752103	1.21089421	-0.6448488	-0.6670593	-0.6654982	-0.6597698	-0.6447776	-0.6511
A_52_P541175	Marveld1	1.25132734	1.31574003	1.4234647	-0.6151348	-0.7342695	-0.7301188	-0.707822	-0.576109	-0.627078
A_51_P305311	Plau	1.07616626	1.48406671	1.41581998	-0.6591153	-0.6652798	-0.6658615	-0.6621317	-0.6608847	-0.6627799
A_51_P438967	Gpnmb	0.51740845	1.69227228	1.57513046	-0.6309887	-0.630996	-0.6309964	-0.6309915	-0.6298461	-0.6309924
A_52_P297773	Uggt2	1.28170924	1.42955183	1.27567016	-0.5376709	-0.7487215	-0.7555319	-0.700527	-0.6404483	-0.6040317
A_51_P461416	Kcnq5	1.33573412	1.3663618	1.28689129	-0.492958	-0.7110879	-0.7260368	-0.7259191	-0.638085	-0.6949004
A_52_P81562	Eef2	2.09342675	0.76938372	0.69468258	-0.1057319	-0.788901	-0.8354142	-0.8389943	-0.5920486	-0.3964031
A_51_P191199	Gypc	1.0240454	1.48152139	1.46091259	-0.6610789	-0.6610805	-0.6610816	-0.6610837	-0.6610742	-0.6610806
A_52_P552665	Fzd7	0.22407124	1.65988722	1.51938117	-0.4550711	-0.4872203	-1.0959919	-0.9922467	0.11882355	-0.4916331
A_51_P292008	Gpx3	0.11037231	1.68075067	1.75110068	-0.5475223	-0.5877991	-0.6229181	-0.6329212	-0.5664785	-0.5845844
A_51_P224311	Popdc2	0.86197372	1.49560362	1.56069583	-0.5624049	-0.611704	-0.6976879	-0.7153044	-0.5967368	-0.734435
A_51_P302831	Brinp1	1.21184524	1.4369248	1.34441236	-0.6355099	-0.6824913	-0.6699593	-0.6692426	-0.682394	-0.6535853
A_51_P483908	Dctn1	-0.1163561	1.52487173	1.53053423	0.62393682	-1.0921262	-0.8331169	-0.4778363	-0.8459912	-0.3139161
A_51_P349988	Ggta1	0.54178915	1.61789236	1.63842982	-0.630016	-0.6396044	-0.638642	-0.633754	-0.6317644	-0.6243305
A_52_P27003	Uggt2	1.10482979	1.23002112	1.36873972	0.25665581	-1.1195969	-0.8866588	-0.7326313	-0.6597178	-0.5616417
A_52_P64356	Sparcl1	0.46134447	1.62957788	1.66678092	-0.6180153	-0.6279516	-0.6271723	-0.6301973	-0.6277885	-0.6265783
A_51_P470432	Pdlim4	1.04055914	1.40448437	1.49208153	-0.3258148	-0.7402625	-0.7213909	-0.7586318	-0.7155978	-0.6754272
A_52_P380418	Rarg	1.20242631	1.28003601	1.33137021	-0.1575051	-0.5000237	-0.8991838	-1.0016334	-0.9940604	-0.2614262
A_51_P287069	Serpinh1	1.36564173	1.32192903	1.31202222	-0.6665356	-0.666656	-0.6666167	-0.6666311	-0.666671	-0.6664826
A_51_P447835	Pla2g7	0.13792666	1.68446659	1.74014201	-0.5942787	-0.5969797	-0.5920962	-0.5952312	-0.6008106	-0.5831389
A_51_P288876	Tmem45a	2.18968081	0.71577433	0.71381218	-0.6058433	-0.6062954	-0.6063096	-0.605897	-0.6062696	-0.5886525
A_51_P195244	Mfap4	2.42007725	0.40580083	0.44369589	-0.5448827	-0.5448902	-0.5451508	-0.5448857	-0.5449319	-0.5448326
A_51_P479352	Klhdc9	1.90587698	0.96925155	0.93424747	-0.3314591	-0.7008645	-0.7299545	-0.5655991	-0.786217	-0.6952817
A_51_P296036	Nrbp2	1.53432142	1.13850907	1.29049682	-0.6918289	-0.7726533	-0.6879526	-0.5118137	-0.7712308	-0.527848
A_52_P28379	Mfap2	2.23065954	0.62298567	0.71582128	-0.5948644	-0.5973435	-0.5956891	-0.5864491	-0.6020023	-0.593118
A_51_P427934	Megf8	1.82706733	1.04412536	0.98181484	-0.6714287	-0.8763575	-0.7885879	-0.5129665	-0.511018	-0.4926489
A_51_P408506	Icam1	1.83626337	1.05372851	0.98784949	-0.6897412	-0.7168783	-0.6925755	-0.6607196	-0.6337886	-0.4841381
A_51_P107686	Foxc1	1.55834981	1.14329602	1.23920124	-0.7569226	-0.7772809	-0.7737276	-0.6710335	-0.6226339	-0.3392486
A_52_P405177	C1qtnf6	1.82005621	1.06249811	1.00722693	-0.6704317	-0.7206871	-0.7073802	-0.6471529	-0.5455236	-0.5986057
A_51_P366986	Glis2	1.78359304	1.05950191	1.04853169	-0.6252628	-0.7647308	-0.7531317	-0.7210934	-0.5689289	-0.4584791
A_52_P108447	Glis2	2.14141976	0.74241506	0.76866902	-0.5975585	-0.7206359	-0.7040208	-0.6304164	-0.5383721	-0.4615003
A_51_P520310	Alox12	1.85388002	1.61448956	-0.0441096	-0.5669383	-0.576074	-0.5852657	-0.5670382	-0.5652103	-0.5637335
A_51_P486668	Uggt2	1.39156219	1.32656855	1.25865433	-0.5579335	-0.8074941	-0.8203836	-0.6812148	-0.5958543	-0.5139047
A_51_P502132	Mmp23	1.8382167	0.96272843	1.07746182	-0.6650257	-0.6962039	-0.7018925	-0.6802665	-0.6253077	-0.5097105
A_51_P284426	Cstad	1.96002135	0.9594022	0.89877016	-0.6393036	-0.6418878	-0.6408084	-0.6418963	-0.6321164	-0.6221813
A_52_P681456	Rftn2	1.54675948	1.15152443	1.24675582	-0.7385385	-0.7686745	-0.7629406	-0.7351194	-0.3541957	-0.5855711
A_51_P374571	Igfbp6	1.40385475	1.29687554	1.29735854	-0.668059	-0.6700781	-0.6690885	-0.6637703	-0.6629328	-0.6641602
A_51_P245631	Rftn2	1.84150073	1.00749514	1.02114795	-0.7035522	-0.7204257	-0.6918085	-0.6852039	-0.4411997	-0.6279538
A_51_P386182	Magi2	1.49171969	0.93383548	0.95332304	-1.1215407	-1.1287074	-1.1072373	-0.3933313	0.26411001	0.10782845
A_52_P385229	Rai14	1.6537446	1.1447994	1.15753347	-0.6808939	-0.6847002	-0.6709325	-0.6797263	-0.6471863	-0.5926382
A_52_P613241	Icam1	1.76392286	1.0538604	1.10081582	-0.6673174	-0.68622	-0.6620017	-0.6650902	-0.6581813	-0.5797886
A_51_P498152	Sat2	1.93486154	0.81678071	0.86398597	-0.8727804	-0.8502124	-0.7772845	-0.8149665	-0.2995989	-0.0007856
A_51_P419925	Arhgef40	1.26838536	1.32017	1.405955	-0.6984384	-0.7198149	-0.6941961	-0.6713619	-0.6142795	-0.5964196
A_52_P251615	Arhgef40	1.2002125	1.39367878	1.39797918	-0.6957813	-0.7070746	-0.6898269	-0.6524715	-0.6251864	-0.6215297
A_52_P418477	Tpm2	0.88792272	1.47405621	1.5693493	-0.6557018	-0.6558966	-0.6558437	-0.6556873	-0.6544573	-0.6537415
A_52_P591542	Atp8b2	0.80616466	1.4936291	1.60538336	-0.6543923	-0.6579924	-0.6549368	-0.648372	-0.6416468	-0.6478369
A_51_P274789	Rai14	0.7917227	1.64562354	1.43414162	-0.6916039	-0.7753659	-0.7573717	-0.6564713	-0.6248422	-0.3658329
A_51_P370717	Gsc	0.59725535	1.54399282	1.60417295	-0.7372504	-0.8074352	-0.8079536	-0.7252814	-0.5445951	-0.1229055
A_52_P315976	Tpm2	0.98626864	1.46706388	1.50466986	-0.6601044	-0.6601581	-0.660181	-0.6599852	-0.6591244	-0.6584492
A_51_P216965	Fkbp10	1.29047821	1.32759321	1.38089533	-0.6664805	-0.6667929	-0.6669833	-0.6667313	-0.6663548	-0.665624
A_51_P435968	Tnfaip8	1.00337434	1.41695969	1.4290297	-0.5574867	-1.0437295	-0.870706	-0.7413873	-0.4466934	-0.1893608
A_52_P198898	Samd5	1.09399042	1.12560763	1.14624544	-0.7965793	-1.1281409	-1.1638914	-0.4773265	-0.4968492	0.69694374
A_51_P364485	Tnfaip2	0.430003	1.66247956	1.59958304	-0.7630921	-0.7550106	-0.793235	-0.4943499	-0.6352906	-0.2510873
A_51_P435731	Arhgef40	1.68637403	1.03620352	1.13619435	-0.8212467	-0.8078878	-0.8505142	-0.5911814	-0.5952947	-0.1926472
A_51_P172952	A830018L16Rik	1.74826709	1.14272096	0.87637365	-0.7948802	-0.7802315	-0.7956716	-0.6871148	-0.723673	0.01420935
A_52_P453785	Cthrc1	1.83432926	1.01342857	1.04221648	-0.6484463	-0.6484504	-0.6484676	-0.6480325	-0.6483588	-0.6482189
A_51_P115346	P2rx6	1.98463192	0.80688172	1.00007389	-0.6683007	-0.6424785	-0.6645391	-0.6335716	-0.6554338	-0.5272638
A_52_P238479	MARCH11	1.60953423	1.21677224	1.14045085	-0.6760075	-0.6767196	-0.6778698	-0.5923582	-0.6706192	-0.673183
A_52_P284441	Cep112	1.9573883	0.86941395	0.60326149	-0.7576361	-1.0617498	-1.0107807	0.07043409	-0.1158595	-0.5544717
A_51_P174158	Irs3	0.98763522	2.06302939	0.32282257	-0.7992583	-0.7832617	-0.8600927	0.16249155	-0.3916481	-0.7017179
A_52_P154101	Calca	1.50404086	1.45624152	1.00006218	-0.671213	-0.6707987	-0.6736007	-0.6100374	-0.6650054	-0.6696894
A_52_P604629	Csrnp1	0.75675147	1.47647133	1.56228838	-1.1173374	-0.7105635	-0.7032226	-0.2918989	-0.504643	-0.4678458
A_52_P203560	Fzd10	0.25081443	1.81285116	1.52616961	-0.7114163	-0.7109381	-0.7109209	-0.2697132	-0.7106127	-0.476234
A_51_P192130	Stk10	1.06866383	1.46005442	1.40048898	-0.8842614	-0.7659299	-0.6768305	-0.5198721	-0.7438818	-0.3384316
A_52_P643165	Samd4	0.94529927	1.52811021	1.47436437	-0.6606239	-0.6616146	-0.6602436	-0.6555674	-0.6556497	-0.6540746
A_51_P360918	Ehd3	0.48560071	1.65021974	1.63438446	-0.6358774	-0.639679	-0.6374079	-0.6101931	-0.6278081	-0.6192393
A_51_P184853	Tenm2	1.0794049	1.3113737	1.54853559	-0.8147458	-0.7653496	-0.656263	-0.3724685	-0.5841918	-0.7462955
A_51_P252859	Cyr61	0.84921779	1.52915585	1.54217746	-0.6618631	-0.666034	-0.6551827	-0.6438813	-0.6460509	-0.6475392
A_51_P310821	Hoxa5	0.15095179	1.60458871	1.68064071	-0.8456071	-0.8262032	-0.811699	-0.6343957	0.02569875	-0.343975
A_52_P144297	Tspyl3	1.02308966	1.50968698	1.43248044	-0.6840988	-0.6734955	-0.6649152	-0.6618288	-0.6360329	-0.6448859
A_51_P465281	Lgals1	1.24361304	1.37641296	1.37641296	-0.6892876	-0.6838617	-0.6776354	-0.6391773	-0.6384268	-0.6680502
A_51_P312327	Ptges	0.93532498	1.48446964	1.43249468	-0.8728132	-0.854698	-0.8527714	-0.6193517	-0.4896825	-0.1629726
A_51_P430728	Hoxd9	0.3662191	1.17016759	1.10668619	-1.3192905	-1.1451031	-1.3122017	0.3614106	0.57244172	0.19967009
A_51_P106322	Gpsm3	0.1766886	1.64988561	1.75270722	-0.5796183	-0.56888	-0.6704846	-0.5057427	-0.5204748	-0.7340809
A_52_P224829	Gpc6	1.18225595	1.36945313	1.43941177	-0.6681649	-0.6616281	-0.6682935	-0.6566636	-0.6681212	-0.6682495
A_52_P168953	Vcan	1.15667388	1.38841722	1.44304703	-0.6809538	-0.6684866	-0.6761264	-0.6481223	-0.6440243	-0.6704248
A_52_P484956	Nnat	0.29145599	1.6372639	1.73223821	-0.6105558	-0.6103629	-0.6104579	-0.6088594	-0.6103332	-0.6103891
A_52_P380301	Unc5c	2.45798529	0.3433039	0.38508127	-0.5319696	-0.53119	-0.5318218	-0.5305262	-0.5296493	-0.5312135
A_52_P326354	Erf	1.37148135	1.18183695	1.36718932	-0.6758863	-0.5725	-1.0009864	-0.450287	-0.3669613	-0.8538867
A_51_P122290	Zmynd8	1.42331092	0.59755534	0.62499219	-0.9436924	-1.122626	-1.4527377	0.27615066	0.89464234	-0.2975953
A_51_P506015	Zmynd8	1.70275496	0.2766761	0.40462136	-0.5432839	-0.9957978	-1.688206	0.19626591	0.81120507	-0.1642358
A_51_P371876	Zmynd8	2.55951139	-0.0402918	0.11712955	-0.6532809	-0.5048243	-0.5711427	-0.4661723	0.01533781	-0.4562667
A_52_P255304	Amer3	1.71774173	0.03744522	-0.4807739	-0.9173368	-0.8896193	-0.9424238	-0.3156673	1.43978683	0.35084732
A_52_P725041	Dnah7a	2.25673105	0.32742585	0.53331673	-0.8312237	-0.8255712	-0.6593088	-0.8330768	-0.0672794	0.09898629
A_51_P372858	Rarg	1.70073153	1.03554435	1.16600872	-0.7840818	-0.7021058	-0.6083973	-0.8611271	-0.5371612	-0.4094115
A_51_P295621	Adamtsl3	2.54972186	0.22084095	0.15471692	-0.487667	-0.4875846	-0.4875811	-0.4876719	-0.4875246	-0.4872505
A_51_P278163	Plcd3	2.14177214	0.46206466	0.57325566	-0.8231414	-0.7958061	-0.7675668	-0.9385803	0.27246145	-0.1244593
A_51_P372853	Rarg	1.74528349	1.08163285	1.07499962	-0.7878854	-0.6765197	-0.6595292	-0.7842591	-0.552566	-0.4411566
A_51_P320452	Trhde	1.29700535	1.18721822	1.49946003	-0.7014417	-0.6650314	-0.7006955	-0.7022944	-0.5609316	-0.653289
A_52_P297093	Matn3	1.43584565	1.28423364	1.27582586	-0.6709324	-0.6645467	-0.6681256	-0.6708318	-0.6562516	-0.6652172
A_52_P398099	Uggt2	1.69595837	1.14387644	1.10516652	-0.6597704	-0.6552472	-0.65976	-0.6602728	-0.6535888	-0.6563622
A_51_P421724	St6galnac5	1.92744757	1.34711474	0.42834354	-0.6286744	-0.6164625	-0.6261133	-0.6337935	-0.5858334	-0.6120288
A_51_P488196	Bmper	1.81514371	1.04639913	1.03753812	-0.6504993	-0.6504233	-0.6504892	-0.6504955	-0.6467607	-0.650413
A_52_P437421	Bmper	1.84554733	1.06681626	0.97087544	-0.6476049	-0.6471852	-0.6475339	-0.6475927	-0.6461917	-0.6471306
A_52_P421673	Samd4	1.377763	1.32093653	1.29993525	-0.6944317	-0.6672604	-0.6813542	-0.6711171	-0.6393015	-0.64517
A_51_P116946	Atp6v0a4	1.38978407	1.18665051	1.25165228	-0.7924366	-0.810254	-0.8854606	-0.8863741	0.03465889	-0.4882203
A_52_P460704	A830018L16Rik	2.02621576	0.14752442	1.34227152	-0.5961964	-0.6097381	-0.629399	-0.6200793	-0.5117758	-0.5488231
A_51_P381506	Eva1b	2.11080088	0.83471334	0.75327035	-0.6184805	-0.6205689	-0.6209521	-0.6210614	-0.6064761	-0.6112455
A_51_P314186	Syne1	1.73210956	1.13312996	1.06688987	-0.6622328	-0.6563597	-0.663545	-0.6630086	-0.6344564	-0.6525269
A_51_P290886	Cdh8	2.22957285	0.72913491	0.60932363	-0.6066212	-0.6053203	-0.6147718	-0.6136175	-0.5377271	-0.5899735
A_52_P309166	St6galnac5	2.27954477	0.16064007	0.91503141	-0.605449	-0.6035508	-0.6371704	-0.632087	-0.3392394	-0.5377197
A_52_P489240	Rexo2	1.46436652	1.19576955	1.29358838	-0.6995313	-0.578341	-0.7970846	-0.885563	-0.4077786	-0.585426
A_51_P190199	Pnma1	1.83991204	1.25680618	0.70033894	-0.7282194	-0.5683377	-0.7821891	-0.7896227	-0.3529409	-0.5757473
A_51_P183882	Tnfsf12	1.69020157	1.11410675	1.14240462	-0.6634855	-0.6586425	-0.6672852	-0.6679143	-0.6338055	-0.65558
A_51_P118443	Ppp1r18	1.50890902	1.26745709	1.21094146	-0.6671963	-0.6657249	-0.6720708	-0.6765134	-0.6605538	-0.6452485
A_51_P243900	Nell2	1.89532489	1.03973611	0.92092999	-0.639936	-0.6343147	-0.6556864	-0.6538038	-0.6386605	-0.6335895
A_52_P167317	Nol4	1.06841625	1.61996809	1.2213881	-0.7245363	-0.6844419	-0.7995571	-0.8072044	-0.2760341	-0.6179986
A_52_P417819	Gpnmb	0.32555071	1.69827172	1.6583474	-0.6153788	-0.6136114	-0.6158074	-0.6167166	-0.608656	-0.6119995
A_51_P315042	Avpr1a	1.24162331	1.36490677	1.39030423	-0.6666546	-0.6662627	-0.6669383	-0.6671406	-0.6637093	-0.6661287
A_51_P188108	Lama1	0.58796844	1.72733444	1.49868128	-0.6392514	-0.6306974	-0.6400575	-0.6414346	-0.627953	-0.6345903
A_52_P385584	Hoxd9	0.66935713	1.47652523	1.70354848	-0.6439146	-0.6416511	-0.6443576	-0.644248	-0.6345444	-0.6407151
A_51_P202942	Cox4i2	0.2524572	1.18226909	1.89595854	-0.6760008	-0.2091874	-1.2537919	-0.8617023	0.02752704	-0.3575296
A_52_P655972	Rai14	0.94998111	1.61171944	1.37859472	-0.6314175	-0.6774052	-0.7303998	-0.6800214	-0.6248589	-0.5961925
A_51_P451335	Pla2r1	0.18627766	1.74614762	1.65723833	-0.6255029	-0.622093	-0.639912	-0.6346175	-0.6028018	-0.4647364
A_52_P555776	Sntg1	-0.4877924	2.21339062	1.19574117	-0.5044259	-0.4892306	-0.5235825	-0.5362018	-0.4105345	-0.4573641
A_51_P319572	Rusc2	0.82311762	1.54649469	1.53999075	-0.7115844	-0.6495872	-0.6981022	-0.6531725	-0.6121982	-0.5849585
A_51_P308298	Myl9	1.18476034	1.38352594	1.4234046	-0.6755273	-0.6634094	-0.6742459	-0.6717404	-0.6550018	-0.6517661
A_52_P302433	Plau	0.93374578	1.47026858	1.53917503	-0.6716869	-0.6711029	-0.6712494	-0.6715321	-0.6706074	-0.5870108
A_51_P295410	Nap1l5	0.17951862	1.7501161	1.65873844	-0.6141952	-0.6135644	-0.6135935	-0.6140975	-0.6132961	-0.5196265
A_52_P5491	Frem2	0.1684747	2.29974726	0.67091881	-0.6758865	-0.6561534	-0.6715014	-0.6730974	-0.6401266	0.17762461
A_52_P251703	Vcan	0.62401426	1.66651882	1.54371837	-0.6398972	-0.6389971	-0.6397943	-0.6399266	-0.6367288	-0.6389075
A_51_P210510	Sparcl1	0.72406632	1.61797942	1.53417253	-0.6469184	-0.645925	-0.6467994	-0.6469136	-0.6437942	-0.6458677
A_51_P391904	Apbb1ip	1.23554759	1.50985903	1.24194773	-0.6834139	-0.6621707	-0.6646105	-0.6662223	-0.6336509	-0.6772861
A_51_P312885	Gramd1a	0.90289343	1.53266539	1.50083674	-0.6686178	-0.6506605	-0.6468029	-0.6620018	-0.6407535	-0.6675591
A_51_P461364	Gpx7	1.22636928	1.3659162	1.40336198	-0.6659481	-0.6659385	-0.665938	-0.6659456	-0.6659358	-0.6659416
A_51_P291417	Thbd	0.52959667	1.67474613	1.58710598	-0.6320125	-0.6318935	-0.6319865	-0.63201	-0.6316469	-0.6318994
A_52_P330359	Agbl2	1.07121808	1.58329272	1.20153313	-0.7904992	-0.6413479	-0.7883013	-0.7901401	-0.0606716	-0.7850839
A_52_P33221	Apbb1ip	1.09227002	1.45135632	1.43467109	-0.6855284	-0.6533593	-0.6594853	-0.6855125	-0.6097698	-0.6846421
A_52_P156314	Esr2	0.52793561	1.7221124	1.52297684	-0.7126949	-0.6408449	-0.6465323	-0.7113736	-0.4083015	-0.6532777
A_52_P467221	Apbb1ip	-0.4721647	1.90490906	1.61169942	-0.5645077	-0.4797977	-0.4947249	-0.5276449	-0.4694604	-0.5083081
A_51_P380709	Dok5	0.13744492	1.55611502	1.85905829	-0.5928311	-0.5917	-0.5924424	-0.5922385	-0.5916063	-0.5917999
A_51_P211702	Cthrc1	0.9447127	1.31817075	1.67066897	-0.6566957	-0.6548601	-0.656314	-0.6558255	-0.6548309	-0.6550262
A_52_P655068	Fut9	-0.3886892	1.72389473	1.79441801	-0.5941891	-0.4693425	-0.576487	-0.5800784	-0.4284851	-0.4810414
A_51_P405227	Nradd	0.88247285	1.50741933	1.53836045	-0.6845894	-0.6460517	-0.6615537	-0.6818636	-0.5669412	-0.687253
A_52_P467449	Alox12	1.70690791	0.9288524	1.11310715	-0.850569	0.08603661	-0.6748602	-0.8693985	-0.6670233	-0.7730531
A_51_P372112	Gpc6	0.84732362	1.38793583	1.67514796	-0.6598997	-0.6242399	-0.6483086	-0.6600623	-0.658382	-0.6595149
A_52_P543079	Dhtkd1	1.25715684	1.34421358	1.36224824	-0.7358246	-0.32144	-0.7306734	-0.740282	-0.7114114	-0.7239874
A_52_P209383	Tgfb1i1	0.94742937	1.49551832	1.50570774	-0.6581897	-0.6577661	-0.6581906	-0.6581971	-0.6581403	-0.6581716
A_52_P477431	Mnt	1.39889089	1.06594182	0.98387451	-1.4775015	-0.8457106	0.2561626	0.01446511	-0.6876164	-0.7085064
A_52_P219233	Loxl3	1.43289081	1.22676704	1.33280179	-0.6863748	-0.6853497	-0.5796153	-0.6781345	-0.6833023	-0.6796831
A_51_P353056	Fat4	1.65862925	1.5580284	0.61349828	-0.6425662	-0.6414593	-0.6326194	-0.6383056	-0.6420314	-0.6331741
A_52_P623211	Ccdc141	2.08444828	0.63906042	0.90052511	-0.7440406	-0.5449128	-0.482967	-0.2724838	-0.9124646	-0.667165
A_52_P664404	Zfp286	1.44174555	1.24272841	1.31029996	-0.6728576	-0.6728026	-0.6610278	-0.6504763	-0.6647876	-0.672822
A_52_P675530	Fat4	1.99561472	0.84519303	0.95177423	-0.6332286	-0.6330653	-0.6316231	-0.6285652	-0.6330287	-0.6330712
A_51_P297900	Syt1	1.58367772	1.09269946	1.28023939	-0.7171873	-0.6968399	-0.6933812	-0.4571699	-0.6828031	-0.7092351
A_51_P326015	Csrnp1	0.25585834	1.52665788	1.81074004	-0.9370228	-0.5827395	-0.5025185	-0.3964658	-0.5238935	-0.6506161
A_52_P664405	Zfp286	1.32646035	1.28472718	1.38745012	-0.670486	-0.666368	-0.6600422	-0.6613955	-0.6700008	-0.6703451
A_51_P116088	Rexo2	1.41879025	1.26155922	1.30515075	-0.8160581	-0.6265028	-0.5606013	-0.606198	-0.6380851	-0.7380549
A_52_P515282	1110051M20Rik	1.5559384	1.17117308	1.16268717	-0.8527678	-0.7930662	-0.7010112	-0.7006894	-0.1362511	-0.7060129

Supplementary Table 1: Genes associated with iISC-high DMRs

Probe_ID	Gene_symbol	MEF_1	MEF_2	MEF_3	ISC_1	ISC_2	ISC_3	iISC_3	iISC_1	iISC_2
A_52_P678056	Eva1b	1.7918375	1.06602619	1.05147375	-0.6590566	-0.653204	-0.6512001	-0.6520594	-0.641724	-0.6520933
A_52_P26409	Rarg	1.47037268	1.44643572	1.02479473	-0.8992579	-0.6650477	-0.6383837	-0.6162147	-0.444753	-0.6779461
A_51_P372522	Fam189b	1.84061132	0.97164263	1.07081282	-0.6895445	-0.6799186	-0.6303876	-0.6161248	-0.5893806	-0.6777107
A_51_P359983	Arhgap17	1.75467534	0.97205797	0.9910786	-1.3042575	-0.7846183	-0.3653274	-0.4456869	-0.3359248	-0.4819971
A_51_P118046	Cyp2r1	1.56613064	0.62003225	0.38049365	-1.1761121	0.35696992	0.83305697	-0.2311248	-1.1744619	-1.1749846
A_52_P68306	Dlgap2	2.54815541	0.1665393	0.19475998	-0.5151936	-0.517728	-0.3257117	-0.5168453	-0.5169985	-0.5169775
A_51_P382524	Atp8b2	1.63402556	1.23087504	1.09546797	-0.6635571	-0.6685015	-0.6572094	-0.636223	-0.6664906	-0.6683869
A_52_P203691	Arl5c	0.54391884	1.4873959	1.75820453	-0.6432805	-0.652276	-0.6243327	-0.645053	-0.5595174	-0.6650596
A_51_P409010	Comp	2.6649036	-0.2706933	-0.2704699	-0.3525784	-0.3564814	-0.3490326	-0.3544852	-0.3481747	-0.362988
A_52_P119792	Zfp709	1.36533431	1.20924846	1.27771695	-0.8505799	-0.6626924	-0.2343235	-0.8853591	-0.2248327	-0.9945121
A_51_P417507	Gabra2	-0.1146963	1.41193658	1.61602019	-0.6632608	-0.756361	-0.6485096	-0.9245385	0.77837672	-0.6989673
A_52_P385718	Galntl6	0.59172937	0.49893335	1.11739646	-1.2327173	-1.0844112	1.40122843	-0.8758248	0.35735476	-0.7736891
A_52_P30803	Gm973	0.10076677	0.43027101	0.40153341	-0.4679009	-0.6932091	-0.699628	-0.7144114	-0.6944811	2.3370594
A_51_P506961	Cldn9	-0.1953684	1.43389739	1.88858989	-0.4066492	-0.3425222	-0.8060313	-1.046615	-0.556974	0.03167279
A_52_P534894	Tead3	-0.2504396	1.69454841	1.66480836	-0.1920246	-0.3626516	-0.5172527	-0.8098074	-1.0876602	-0.1395206
A_52_P616600	Rusc2	0.46721334	1.44834749	1.04211556	-0.9646659	-1.0523541	-0.1755318	-1.1316943	-0.5852321	0.95180189
A_52_P576208	A930018M24Rik	-0.4141631	0.84353275	0.98774514	-0.7493462	-0.7190619	-0.4634646	-0.7463785	-0.6914752	1.95261178
A_52_P72230	Nol4	-1.066483	1.84838035	1.07389699	0.34967808	-0.4562139	-0.8401735	-0.9384324	0.44795538	-0.418608
A_52_P145970	Dnah10	-0.69875	1.13370349	1.79229477	0.97859589	-0.6372834	-0.6408004	-0.6636689	-0.6194148	-0.6446765
A_51_P322138	Hspb2	-0.5855737	1.52036981	0.81347193	0.67415094	-0.4379136	0.5605369	-1.6214524	0.11603553	-1.0396255
A_52_P62986	Synpo	-2.0289824	1.19185767	0.75989768	0.4983838	0.06998714	0.32650675	-0.0357835	-1.1714564	0.38958914
A_52_P1100423	Vcan	-0.5450752	1.33052204	2.12286685	-0.5623487	-0.4541391	-0.4727321	-0.5279587	-0.3992794	-0.4918556
A_51_P298196	Uggt2	-0.5620009	2.482145	0.64272047	-0.4819955	-0.4077271	-0.4402745	-0.4769979	-0.330911	-0.4249587
A_52_P572702	Gxylt1	-1.2669336	1.62316744	1.29491969	-0.8449228	-0.4508098	-0.938751	0.13397248	0.51286496	-0.0635074
A_52_P446146	Gabra2	-0.6327266	2.0590165	1.37048661	-0.6277559	-0.4878155	-0.6201536	-0.4823297	-0.1088876	-0.4698341
A_52_P2645	Rftn2	-0.2994697	2.66617398	-0.3700325	-0.3348877	-0.32145	-0.3476122	-0.3386607	-0.3288996	-0.3251616
A_52_P59400	A830018L16Rik	0.63887645	2.46733792	-0.5867062	-0.4768597	-0.4600855	-0.5205533	-0.5174265	-0.1423087	-0.4022745
A_52_P419089	Fut9	-0.1907289	2.64264061	-0.4792674	-0.467285	-0.2776381	-0.3852489	-0.4573635	-0.0848854	-0.3002235
A_51_P142371	Kcnj6	-0.2941216	2.66636828	-0.3412322	-0.3432371	-0.3368098	-0.3377051	-0.3385391	-0.3382179	-0.3365054
A_52_P374075	Csmd2	-0.332982	2.66666619	-0.3343174	-0.3337407	-0.3322549	-0.3336367	-0.3336833	-0.3331137	-0.3329375
A_52_P40695	Fut9	-0.3546408	-0.2677642	-0.3926005	-0.3735459	-0.3247996	-0.3724184	-0.3764036	-0.1992705	2.66144356
A_51_P137560	Etv2	-0.5344116	2.26510611	-0.7785633	-0.3898823	-0.1917633	-0.5675633	-0.6753512	0.99744654	-0.1250177
A_52_P625215	Wfikkn2	0.63617217	0.44720749	0.38870749	0.43596529	-1.3221914	-1.5220122	-1.0015008	0.73519377	1.20245828
A_51_P168894	Ano1	-0.290971	-0.2365763	-0.3023901	-0.1501192	-0.7594466	-0.7279375	-0.6621892	0.75767384	2.37195608
A_52_P661587	Ccdc141	-0.4081673	-0.5656685	-0.7766444	0.57180255	-0.5929921	-0.6730171	-0.7691772	1.50063771	1.71322648

Supplementary Table 1: Genes associated with iISC-low DMRs

Probe_ID	Gene_symbol	MEF_3	MEF_2	MEF_1	ISC_3	ISC_2	ISC_1	iISC_3	iISC_1	iISC_2
A_51_P144090	Hoxa10	-0.2185632	-0.2742856	-0.0355682	-1.0555756	-1.0278578	-1.0604506	1.45910967	1.14077421	1.07241718
A_52_P179640	Slc38a3	0.40956702	-0.4423164	0.17337661	-1.2801354	-0.8534045	-0.8281412	0.61609204	2.03607345	0.16888843
A_51_P108757	Anks6	0.86855113	0.4351308	0.33132768	-0.9689896	-1.4994396	-0.2034403	0.3813014	1.62346148	-0.9679031
A_52_P488422	Foxn3	0.18933953	0.27442688	2.25966531	-0.8296201	-1.0345454	-0.55645	0.20233316	0.26993663	-0.775086
A_52_P622694	Foxn3	0.67399145	0.59891051	1.25064598	-1.5583009	-1.1703471	-0.1806029	0.64629404	0.68843671	-0.9490278
A_51_P275751	Foxn3	0.26982022	0.36177116	1.82772993	-1.3389059	-1.1195608	0.13536724	0.38030817	0.48781686	-1.0043468
A_52_P370560	Tex33	1.04935776	0.99210101	-1.1267105	-0.3660504	-0.9749886	-1.299976	1.23743483	0.57596586	-0.0871339
A_52_P46384	Bcl9l	1.76222585	1.67084223	0.05828079	-0.8048783	-0.5401769	-0.5737677	-0.4813424	-0.5879523	-0.5032312
A_52_P163011	Erp29	1.48231822	1.48231822	0.92093276	-0.7126922	-0.7446309	-0.9543772	-0.2928663	-0.5686745	-0.6123282
A_51_P426232	Luzp1	1.29034321	1.30144131	-0.6669622	-0.5508362	-1.0184288	-0.8816856	0.77464808	-0.9749886	0.72646872
A_52_P448205	Ldb3	1.81840144	1.06284761	-0.8762571	-0.8931799	-0.8808941	-0.1598353	0.13204893	-0.8626783	0.6595467
A_52_P287986	Sbno2	0.1520689	0.30348801	0.75557527	0.28655507	0.07809362	-1.5914135	-1.1626997	-0.5612604	1.73959274
A_51_P387205	Syngr1	1.57148333	1.46837686	-0.2694421	-0.0364561	0.02576745	-0.3957003	-1.1348375	-1.3561572	0.12696552
A_52_P229972	Syngr1	1.41342996	1.45260362	0.29928411	-0.1175841	0.1438418	-0.5613676	-1.1994939	-1.4191612	-0.0115527
A_51_P239737	Xpnpep1	1.37425572	1.12552145	0.95909899	-0.1005471	0.25158799	-1.113961	-0.3894421	-1.3997183	-0.7067957
A_51_P468482	Sbno2	1.20134192	1.15809062	1.57402918	-0.4820012	-0.5844007	-0.8430271	-0.6687033	-0.8872701	-0.4680594
A_51_P142861	Luzp1	1.47058775	1.42977167	-0.0649701	0.24727538	-0.4505865	-1.6217301	-0.5530045	-0.7207724	0.26342885
A_51_P159673	Ldb3	1.30482212	1.23578159	0.60565732	0.58853018	-0.5902937	-1.5360332	-0.6089523	-0.9890999	-0.0104121
A_51_P213260	Klhl22	1.72566218	1.57349472	0.26800209	-0.657601	-0.7797391	-0.3535304	-1.003648	-0.4261156	-0.3465248
A_51_P164459	Irgm1	1.44714739	1.49960717	0.99041148	-0.7583599	-0.6980829	-0.5288562	-0.8458899	-0.4947346	-0.6112425
A_51_P143135	Fam110a	1.5326496	1.29666993	1.08483463	-0.8749451	-0.8074527	-0.3828132	-0.8449389	-0.5754096	-0.4285947
A_51_P128929	Irgm1	1.34370094	1.29017678	1.28178012	-0.89108	-0.7305766	-0.3748709	-0.9886511	-0.4007004	-0.5297788
A_52_P129756	Rnase4	0.72377483	0.72574677	0.92263801	-1.5162832	-1.3419822	0.69557709	0.06563206	0.72040337	-0.9955067
A_51_P158061	Rasl11b	1.51158601	1.46596434	0.95980928	-0.7215767	-0.7712413	-0.5165274	-0.7150187	-0.518166	-0.6948296
A_51_P203501	Naif1	1.958008	1.54685639	-0.376311	-0.5342021	-0.5310565	-0.5075165	-0.5203158	-0.513025	-0.5224376
A_52_P78064	Rnase4	0.9019995	0.89776223	0.72454127	-1.5263546	-1.3694652	0.50617429	0.15129466	0.67258499	-0.9585371
A_52_P16419	Lrsam1	1.38026534	1.29058461	1.31374423	-0.6086868	-0.8700238	-0.6159934	-0.6888065	-0.6191261	-0.5819575
A_51_P232824	Agpat1	1.64533096	1.36319861	0.64323651	-0.5096294	-0.6441902	-0.9430415	-1.1758902	-0.2230341	-0.1559807
A_51_P296057	Plaur	1.56304536	1.60862389	0.61788945	-0.6791624	-0.8096534	-0.7256366	-0.8040497	-0.3728354	-0.3982211
A_51_P486810	Plaur	1.47343691	1.43311135	1.02275912	-0.6931625	-0.8081256	-0.7568513	-0.8043342	-0.425662	-0.4411717
A_52_P72434	Il33	1.17469658	1.35551194	1.37048908	-0.7976936	-0.6338789	-0.7936538	-0.9248879	-0.5981434	-0.15244
A_51_P465582	Agpat1	0.39284467	0.56935424	2.1116601	-0.3104623	-0.6019364	-1.3695612	-0.8373194	-0.0909849	0.13640508
A_51_P293853	Capg	1.12450382	1.09717148	1.64492977	-0.6607222	-0.7336218	-0.790553	-0.9465568	-0.1943854	-0.5407659
A_51_P324583	Pfkm	0.67794519	0.64327439	1.42449182	0.06048878	-0.0360838	0.64453016	-1.7827563	-1.1051496	-0.5267407
A_51_P166873	Dhrs9	-0.1140979	-0.1773261	2.53928198	-0.5239554	0.37002633	-0.5228323	-0.5239449	-0.523268	-0.5238838
A_51_P294891	Zfyve27	0.11525366	0.26722952	-0.951549	-1.3951332	-0.0530281	2.23440981	0.08529375	-0.237964	-0.0645125
A_52_P635015	Ptms	0.33221966	0.50006643	-2.5271781	0.11633931	-0.3769635	0.50006643	0.78789121	0.29110632	0.37645223
A_51_P454949	Cep170b	0.21922073	0.18144677	-2.1389702	-0.747146	-0.386197	0.17807806	0.93894071	0.57418725	1.18043971
A_51_P507602	Ubxn10	-0.3651777	-0.5570538	-0.7263555	-0.6827737	-0.1506234	-0.5377414	-0.0935401	2.39202187	0.72124372
A_52_P293222	Slc25a33	0.79057619	0.8395682	-0.3233735	-1.0380856	0.24284457	-2.0734886	0.06650291	0.8067937	0.68866206
A_51_P230405	Slc25a33	0.73747506	0.54359594	-0.8121236	-0.542748	0.95556705	-2.0725713	-0.1879173	0.61156847	0.76715364
A_52_P526724	Cx3cl1	2.29021528	-0.9921338	-1.142477	0.21463619	-0.0266257	0.41849404	-0.1454516	-0.4350695	-0.1815879
A_51_P187750	Ip6k2	-0.1950334	-0.2574776	-1.5954057	1.04931753	0.44154854	1.67376685	-0.4591947	0.31380948	-0.971331
A_52_P662796	Klhl22	1.25911776	1.19718869	-0.7255047	0.52414151	0.4123899	0.52362215	-0.6529206	-1.0581628	-1.4798719
A_51_P499816	Ckb	-0.1130375	-0.1233126	-0.4882043	1.6396685	1.67567017	-0.7163365	-0.0290412	-0.8213484	-1.0240581
A_52_P219415	Ckb	-0.0411825	-0.0002644	-0.4870404	1.7264444	1.59203869	-0.7028322	-0.2589284	-0.8314765	-0.9967588
A_51_P499020	Zfyve27	-0.5563499	-0.4510267	-0.714223	1.13015376	0.67145725	0.46848627	1.55911717	-0.6193612	-1.4882536
A_52_P511798	Trmt61a	-0.1134795	-0.1624386	-0.6232991	0.84769522	2.14055588	-0.0700103	-0.0844744	-0.5265991	-1.4079502
A_51_P410703	Syt8	-0.4622903	-0.4599134	-0.3549505	-0.3955688	-0.4375806	-0.1216326	-0.1371718	-0.2750486	2.64415653
A_51_P454196	Slc9a4	-0.6523378	-0.4563852	0.08091809	-0.4695449	-0.6408772	0.35486514	-0.3928643	-0.3402026	2.51642894
A_51_P279183	Orai2	-0.9202765	-0.8269997	-0.0645944	-0.7129401	-0.8845283	1.05985946	-0.4245692	1.27959545	1.49445332
A_51_P490767	Lbp	0.32116509	0.46338288	1.42943637	-0.8861302	-0.8860128	-0.3881077	-0.8537072	-0.7977556	1.59772905
A_51_P302181	Kiss1r	-0.51384	-0.4721282	0.82735306	-1.350404	-0.6066898	0.33022656	-0.8619885	0.94939453	1.69807631
A_51_P264336	Nsmce1	-0.4186884	-0.319408	1.79669199	-1.1126741	-1.0559556	0.70996319	-0.889928	0.56307794	0.72692102
A_51_P272023	Fam83g	-1.2775975	-1.2751633	-1.3832079	0.90583487	0.71958326	0.42706403	0.63162473	0.37574142	0.87612043
A_52_P184362	Tmem54	-1.293057	-1.292912	-1.2940273	0.88972839	0.78449547	0.09382823	0.66907066	0.52468297	0.91819065
A_51_P500813	Misp	-1.3023213	-1.3027465	-1.3034076	0.82946447	0.30984449	0.37844364	0.76013843	0.62359964	1.00698468
A_51_P151484	Ppp1r14d	-1.3128258	-1.312844	-1.3129238	0.78028738	0.68727897	0.22113053	0.69826837	0.71373835	0.83788996
A_51_P112627	Sept5	-1.1023337	-1.1225499	-1.3522727	0.97773584	0.87253265	-0.3517254	0.24331659	0.68126941	1.15402711
A_51_P460143	Itga3	-1.1999763	-1.2111867	-1.4980761	0.94040573	0.59964883	0.38059491	0.36675507	0.73889382	0.8829407
A_51_P101765	Itga3	-1.216598	-1.2417498	-1.4734627	0.83782816	0.64726381	0.48231405	0.36013656	0.66651483	0.93775308
A_52_P648738	Batf2	-1.0826575	-1.0753464	-1.0811114	1.01877738	0.4767585	-0.379367	-0.3076232	1.36543337	1.06513626
A_52_P338199	Ldb3	-1.1941271	-1.1878033	-1.1962978	0.77450532	0.67988281	-0.428949	0.42739474	1.21209928	0.91329512
A_51_P194883	Slc39a4	-1.1825862	-1.1823834	-1.1827931	0.07418363	0.19551368	0.05215077	0.66902876	1.13425723	1.42262866
A_52_P474145	Cldn4	-0.7996174	-0.7949322	-0.8038055	-0.0513061	-0.2329399	-0.2464218	-0.0517201	0.61862446	2.36211842
A_51_P291492	Ptk6	-1.1043397	-1.0755651	-1.1276331	0.21901257	0.21209809	-0.144056	0.47909589	0.64640401	1.89498335
A_52_P453428	Lgals2	-1.312962	-1.3129961	-1.3129707	0.82250251	0.6403345	0.52877004	0.2858016	0.83901764	0.82250251
A_51_P318618	Prss32	-1.1632357	-1.1602889	-1.1650072	0.43407952	0.54597808	0.44721007	-0.4171899	1.31486486	1.16358916
A_52_P257426	Sept5	-1.1572807	-1.143034	-1.2406606	0.79574425	0.79951774	-0.5489212	0.52576774	0.86925154	1.09961531
A_51_P337935	Frk	-1.2506098	-1.2593765	-1.3021849	0.53968744	0.62056581	0.45079582	0.08132137	0.95672934	1.16307137
A_52_P523445	Sys1	-1.2942334	-1.2918059	-0.1160196	0.0919396	0.4664429	-0.2018666	0.06003042	0.20451928	2.0809933
A_52_P571290	Frk	-1.3142022	-1.2991104	-1.1419291	0.7039326	0.7052461	0.53285623	-0.1631682	0.87705441	1.0993205
A_51_P184353	Sdcbp2	-1.1496574	-1.1477645	-1.1315869	0.3469185	0.37424791	0.23519758	-0.2240831	1.15047545	1.54625248
A_51_P195066	Frk	-1.3164677	-1.3119473	-1.1515928	0.64192042	0.67044597	0.5060098	-0.0838333	0.98436553	1.06109942
A_51_P133992	Tmem150b	-1.2294508	-1.2331054	-1.2218308	0.4280622	0.54739938	0.15076894	0.29072577	0.73731023	1.53012051
A_51_P241863	Oasl1	-1.121568	-1.1275313	-1.1117156	0.54426555	0.75030147	-0.6989246	0.4744657	1.28389456	1.00681227
A_51_P421182	Slc37a1	-1.2588224	-1.2593709	-1.2590164	0.52656044	0.56233611	0.03481526	0.44588215	1.18727726	1.02033851
A_51_P184385	Skp1a	-0.6790295	-0.211128	-0.5311062	-0.0285667	0.514531	-1.9296686	1.51881034	0.60153922	0.74461849
A_51_P281778	Zbtb7b	-0.742374	-0.8225671	-0.5530404	-0.4289912	0.27099622	-0.9965846	0.63565955	0.50142284	2.13547871
A_52_P185119	Rasd1	-0.8946917	-0.877557	-0.7398491	-0.3725546	-0.3211062	-0.3567023	1.02096858	2.05844307	0.48304921
A_51_P304971	Myl7	-0.8223666	-0.8240996	-0.8324921	-0.4143928	0.07150819	-0.5609972	0.72159959	2.16673348	0.49450706
A_51_P196997	Cldn15	-1.2812165	-1.2816491	-1.2835636	0.56337057	0.09389388	0.43472285	1.04080315	0.82864001	0.88499867
A_51_P256902	Slc51b	-0.9996401	-0.9997436	-1.0005645	0.05423823	0.36534414	-0.6190123	1.48884191	0.22246036	1.48807589
A_52_P610923	Mvp	-0.6392097	-0.6141268	-0.5094685	-0.4965521	-0.3277234	-0.228677	-0.6462215	1.46812976	1.9938491
A_51_P219594	Plekha6	-1.3341326	-1.3296543	-1.3200005	0.56883698	0.71055393	0.66977404	0.52053453	0.66912121	0.84496685
A_52_P669128	Elf3	-1.2976409	-1.2976436	-1.2976402	0.19919774	0.88050633	0.58824618	0.46260905	0.98218041	0.78018506
A_51_P199352	Lgals4	-1.3289209	-1.3289926	-1.3288694	0.61226338	0.71439855	0.83965153	0.54120192	0.65455803	0.62470947
A_51_P467960	Sphk2	-0.6705979	-0.7934982	-1.2680949	0.58353023	0.98913983	1.58412153	-0.0413417	-1.0310028	0.64774391
A_51_P460851	Serinc5	-1.0969274	-1.0701657	-1.2148717	-0.0057909	0.15769862	1.60220105	0.46863584	-0.0086571	1.16787729
A_51_P300038	Tnk1	-1.2742308	-1.2747062	-1.274741	0.52555518	0.74821195	0.80432655	0.76747181	-0.0476744	1.02578694
A_52_P161454	Tmc4	-1.2184099	-1.2195632	-1.2231551	0.41074442	0.3726091	1.49309015	0.33162342	0.11988282	0.93317832
A_51_P143031	Syngr1	-1.1390078	-1.142218	-1.1611066	0.7058428	0.69877791	1.27662624	-0.0611716	-0.3277072	1.14996419
A_51_P246754	Tmc4	-1.2064317	-1.2108344	-1.2161882	0.40827743	0.39311899	1.53691062	0.2415317	0.13785341	0.9157622
A_51_P100991	Rab25	-1.2554262	-1.2546114	-1.255933	0.56061318	0.57872857	0.82306302	0.25742344	0.18813701	1.35800538
A_52_P540554	Rab17	-1.2738435	-1.2723796	-1.2775904	0.59989212	0.60859846	0.97371913	0.13061251	0.37527398	1.13571732
A_51_P279232	Slc9a4	-0.5093219	-0.5220205	-0.511962	-0.3497719	-0.4798594	0.47799393	-0.2138289	-0.4213717	2.53014233
A_51_P424221	Ttc22	-1.3186682	-1.3157599	-1.2958259	0.76185366	0.46984838	0.75121959	0.64913955	0.31654438	0.98164839
A_51_P502872	9030617O03Rik	-1.2015538	-1.2047041	-1.1409964	0.79791897	0.27094951	0.91231011	-0.3105584	0.48922825	1.38740584
A_52_P356204	Tm4sf5	-1.2649449	-1.2649987	-1.2645276	0.93630331	0.67018078	0.87988132	-0.0598819	0.39993045	0.96805732
A_51_P310594	Fxyd3	-1.2427187	-1.2417296	-1.2429561	0.20964322	0.11301378	1.3160821	0.96122128	0.46244997	0.66499406
A_51_P497350	Hal	-0.8746307	-0.8620658	-0.8792311	-0.6966157	-0.6495668	1.68276478	1.05967785	0.84773816	0.37192935
A_51_P419226	Ehf	-1.286082	-1.2861347	-1.2858088	0.12720956	0.55308978	1.04216522	0.57169145	0.98277374	0.58109569
A_51_P139780	Plekha6	-1.3319642	-1.3303169	-1.3150525	0.57743348	0.44778431	0.81960789	0.69290941	0.76839052	0.67120807
A_51_P464669	Blnk	-1.2328966	-1.2356182	-1.2948505	0.34365763	0.05939904	1.15207336	0.51945197	1.10114947	0.58763383
A_51_P107652	Reg4	-0.8982777	-0.898223	-0.8982575	-0.3067148	-0.3829968	0.93838199	0.10819393	2.09456162	0.24333222
A_52_P218470	Adck5	-1.3923113	-1.4200924	-1.0849081	0.28617139	0.42589582	0.82791381	0.96578935	0.79094294	0.60059857
A_51_P275435	Cfb	-1.3098858	-1.3101827	-1.308596	0.49045036	0.39696999	0.67742222	1.05718924	0.76923821	0.5373945
A_51_P312348	Tmem91	-1.3022375	-1.4375008	-0.7818041	0.64968878	-0.2715596	0.97228748	0.67370272	1.30114018	0.19628287
A_52_P410685	Ehf	-1.2771941	-1.2772373	-1.2770647	0.34015599	0.54559911	0.62744237	0.6997351	1.30926901	0.30929447
A_51_P287198	Fcgbp	-1.1586766	-1.1586778	-1.1586719	0.10868291	0.0276671	0.76688571	1.28984458	1.30669785	-0.0237518
A_52_P708817	Hyal2	-0.6798647	-0.7021095	-1.1228401	-0.4286908	-0.6565487	0.59000914	0.63837148	2.08218152	0.27949165
A_51_P110672	Tff3	-1.1600219	-1.1600104	-1.1600221	-0.0978011	-0.178953	0.56397088	1.13175214	1.34560011	0.71548522
A_51_P448741	Ehf	-1.2800317	-1.2776374	-1.2823452	0.41704226	0.46781728	0.7673863	0.70805814	1.24333107	0.23637914
A_51_P181297	Fcgbp	-1.1403495	-1.1391647	-1.1438992	0.09672326	-0.0365362	0.90364815	1.23637669	1.31494736	-0.0917458
A_52_P665119	Slc44a3	-1.3128821	-1.3109938	-1.2995475	0.33363366	0.36913418	0.7938629	0.67455406	0.89647741	0.85576124
A_52_P634250	Kcnn4	-1.2642469	-1.258357	-1.1670678	0.0606315	0.02791093	0.54126808	0.88628272	1.16103621	1.01254223
A_51_P441914	Lypla2	-1.3195111	-1.2593651	-1.0468144	-0.043012	-0.0004018	0.54767656	0.94478697	1.14369159	1.03662931
A_51_P234330	Cx3cl1	-1.4502948	-1.4373529	-0.726682	0.29283802	0.01939372	0.57212416	0.480343	0.87184291	1.37778783
A_51_P440238	Mettl7b	-1.0591114	-1.0591076	-1.0586252	-0.2707879	-0.3996505	1.11880719	0.24303797	1.2018306	1.28360682
A_52_P517762	Slc44a4	-1.1908394	-1.1908821	-1.190729	0.14352996	0.04565131	0.36806445	0.55235296	0.87801679	1.58483505
A_52_P685999	Ggt6	-1.2369717	-1.2369717	-1.2318136	0.29160022	0.13907897	0.31677364	0.68944688	0.84414913	1.42470816
A_51_P196925	Rtkn	-1.3656674	-1.426832	-1.1301316	0.35743788	0.51859267	0.55449995	0.75294146	0.73087564	1.00828334
A_51_P520084	Hsd17b2	-0.8986901	-0.8987293	-0.8987044	-0.4557352	-0.2823977	1.04480596	0.30292204	0.06108649	2.02544223
A_51_P389636	Lgals4	-1.2958204	-1.2958696	-1.2957769	0.36071797	0.41726048	0.71097552	0.43003352	0.86087208	1.10760738
A_51_P385351	Cdhr2	-1.3204908	-1.3242098	-1.2591185	0.36007653	0.45048999	0.96207789	0.54444558	0.56540879	1.02132023
A_52_P419059	Serpinb1a	-1.2828741	-1.2856821	-1.1668301	0.15869663	0.15869663	1.09240517	0.40143839	0.75748205	1.16666745
A_52_P272429	Tnfsf10	-1.2262019	-1.2262158	-1.2269083	0.25360616	0.09910691	0.98761678	0.14359301	1.03655438	1.1588488
A_51_P456208	Mst1r	-1.2648129	-1.2674135	-1.2744079	0.40279438	0.21724384	0.73510205	0.39014836	0.76309708	1.29824866
A_51_P284577	Frk	-1.2263254	-1.2229761	-1.3362537	0.4768788	0.49271606	0.49972809	0.09242242	1.02894769	1.19486215
A_51_P380078	Krt23	-1.1664267	-1.166426	-1.1664397	0.22012056	-0.0021188	0.93103164	-0.0662491	1.00963911	1.40686895
A_52_P205877	Krt7	-1.1945244	-1.1942374	-1.2170826	0.16413202	-0.0305824	0.88022938	0.40884996	0.69628058	1.48693482
A_52_P365633	Krt7	-1.2022246	-1.2019721	-1.2288537	0.08434213	-0.0348696	0.87496669	0.61859877	0.68500538	1.40500699
A_51_P413866	2310007B03Rik	-1.2004686	-1.2087933	-1.2097804	0.37478878	0.07166797	0.51441179	0.50843948	0.50271628	1.64701803
A_51_P457430	Cdhr2	-1.3018492	-1.3023116	-1.3025562	0.46340546	0.31061789	0.79418657	0.61310556	0.63231381	1.09308774
A_52_P257758	Slc44a4	-1.2133576	-1.2123736	-1.2131436	0.15115557	0.1259348	0.32064459	0.65093062	0.90838656	1.48182275
A_51_P279841	Nr1h4	-1.22164	-1.2198426	-1.2226532	-0.004592	0.02218982	0.55547787	0.89721265	1.05133955	1.1425079
A_51_P310850	Pglyrp1	-1.2496995	-1.2494486	-1.249714	0.2536253	0.08265946	0.55117502	0.58470074	1.07990302	1.1967985
A_52_P609918	S100a14	-0.7478189	-0.7477033	-0.7482324	-0.2229231	-0.3587804	-0.1323272	0.01717398	0.5210866	2.41952478
A_51_P172155	Spint2	-1.3020042	-1.3012056	-1.3071732	0.36144366	0.37713664	0.69495853	0.65729566	0.73701686	1.08253168
A_52_P377791	Aldh3a1	-0.4661002	-0.4735594	-0.4839816	-0.457533	-0.4358242	-0.260608	-0.1685811	0.13659348	2.609594
A_51_P119387	Nostrin	-1.2212089	-1.2231971	-1.227626	0.30077133	0.24552185	0.50024782	0.41456902	0.61128252	1.5996394
A_51_P399106	2200002D01Rik	-1.1808791	-1.1824074	-1.2224578	-0.0591422	0.12965284	0.70474155	0.36870323	1.02220216	1.41958673
A_51_P472003	Irf2bp2	-1.1869587	-1.2740975	-1.3226134	0.01548073	0.24740128	0.82447904	0.72589686	0.91775812	1.05265356
A_52_P608916	Dennd2d	-1.3112585	-1.3093506	-1.3257607	0.81360833	0.40205869	0.92376276	0.45301299	0.60684284	0.7470841
A_51_P382214	Pbld1	-0.3765671	-0.4128306	-0.4457149	-0.3548303	-0.2991147	2.65591288	-0.3685634	-0.15066	-0.247632
A_52_P149577	Gucy2c	-1.2682402	-1.2677078	-1.2692038	0.53681728	0.52189487	1.30195337	0.10503184	0.73999521	0.59945919
A_51_P416408	Pnpo	-1.2661299	-1.2557834	-1.294015	0.47325177	0.34482074	1.13007505	0.18518836	0.6468013	1.03579115
A_52_P239320	Eps8l3	-1.2564013	-1.2565297	-1.2564932	0.38318504	0.25800754	1.13940737	0.18650145	0.6852922	1.11703068
A_52_P137361	Ldhd	-1.0214499	-1.0145686	-0.8628257	0.17275072	-0.0281453	2.0523539	-0.4834485	0.54907242	0.63626093
A_52_P167411	Ldhd	-0.9080326	-0.9290389	-0.7717839	0.20853683	-0.1511812	2.2029155	-0.5856857	0.37206855	0.5622014

Supplementary Table 1: Genes associated with iISC-low DMRs

Probe_ID	Gene_symbol	MEF_3	MEF_2	MEF_1	ISC_3	ISC_2	ISC_1	iISC_3	iISC_1	iISC_2
A_51_P128336	Arl14	-1.2264851	-1.2265359	-1.226274	0.4745122	0.19156405	1.25230932	-0.0479655	0.80660105	1.00227387
A_52_P8059	Cntd1	-0.9960322	-1.0038537	-0.8856733	0.01517188	-0.2939856	2.01963946	-0.254815	0.75399921	0.64554919
A_51_P497100	Mrln	-0.9291942	-0.916939	-0.8709221	0.05649259	-0.2654721	2.02695091	-0.1399664	1.15028479	-0.1112345
A_51_P480241	Slc5a1	-1.2831042	-1.2830558	-1.2831011	0.42507637	0.90299256	0.0876247	0.8065409	0.60957282	1.01745376
A_52_P430179	1700123K08Rik	-1.0220489	-1.0205492	-1.0194302	-0.077525	0.32100896	0.16836105	0.94588757	-0.2410675	1.94536325
A_52_P46085	Car9	-1.2372388	-1.2372766	-1.234466	0.82622382	1.15912585	-0.248391	0.6966351	0.51946466	0.75592306
A_52_P229943	Abcg5	-1.2685579	-1.2683903	-1.2678366	0.85238854	1.02126242	0.28714417	0.98270405	0.06778745	0.59349814
A_51_P306710	Soat2	-1.1178631	-1.1238402	-1.1017883	1.25050988	1.59759631	0.02336868	0.31306573	-0.0724291	0.23138012
A_51_P246345	Tmem150b	-1.170373	-1.1724743	-1.1580852	1.20410724	1.32498828	0.14675722	0.73304386	-0.2239589	0.31599489
A_52_P496726	Igsf23	-1.1539842	-1.1541833	-1.1539777	0.75277858	1.23403827	0.58093355	-0.13186	-0.2057329	1.23198777
A_51_P473229	Igsf23	-1.1619148	-1.1616944	-1.1605614	0.64934615	1.19761408	0.49998116	-0.0517305	-0.1598801	1.34883977
A_52_P10458	Pla2g12b	-1.2237417	-1.2238226	-1.2224034	0.91400461	1.06676075	0.4316823	0.2415341	-0.1150076	1.13099355
A_51_P215489	Sys1	-1.7108389	-1.5028833	-0.302461	0.65406286	0.93676353	0.20395946	0.36679035	0.27484192	1.07976507
A_51_P437309	Slc5a1	-1.2757185	-1.2757317	-1.2756373	0.53201867	0.9501337	-0.001866	0.90508175	0.52909307	0.91262625
A_52_P252070	Smim24	-1.311004	-1.3113194	-1.3109731	0.67416518	0.87155711	0.48651381	0.76828074	0.28319104	0.84958864
A_51_P353703	Sema4a	-1.3195878	-1.3197832	-1.319136	0.72752649	0.57319063	0.63031459	0.96809295	0.42186624	0.63751607
A_51_P234253	Nr2f6	-1.297988	-1.3910276	-0.5877246	1.18434026	0.43631032	0.54134347	1.44683867	-0.1779421	-0.1541504
A_52_P282713	Ptms	-0.7419197	-0.8277411	-1.592712	0.68729357	0.29301466	-0.553936	1.4611411	1.09296598	0.1818935
A_52_P37697	Cdhr5	-1.272765	-1.2730289	-1.2732829	0.83101005	0.72471339	0.03932757	1.16526481	0.49364615	0.56511486
A_52_P357469	Slc7a15	-1.0330123	-1.0464944	-1.0892507	1.2043794	0.42689397	-0.3103063	1.67652989	0.30562149	-0.1343611
A_51_P464530	Eps8l2	-1.2755204	-1.2662428	-1.3089126	0.81547996	0.75579977	0.48218283	1.10232778	0.09139178	0.60349366
A_52_P344601	Anks4b	-1.2638572	-1.2638568	-1.263914	0.84989373	1.01270722	0.21220702	1.10221873	0.23256167	0.38203962
A_52_P563375	Cdhr5	-1.2707283	-1.2707087	-1.2620441	0.90059713	0.88997243	0.20583688	1.1444493	0.37003066	0.29259476
A_52_P375047	Slc7a15	-1.040379	-1.0192488	-0.9023136	0.71138323	0.81884097	0.4485566	1.81455275	-0.2598182	-0.5715741
A_51_P356467	Slc7a9	-1.2809611	-1.2807914	-1.2812345	0.78065468	0.5314729	0.79344048	1.14982856	0.11918053	0.46840977
A_51_P103222	Kcnq1	-1.2031743	-1.2034862	-1.2038944	0.35255293	0.34684982	1.3112446	1.28031584	0.14579161	0.17380002
A_52_P389874	Dalrd3	-1.0854187	-1.2177947	-1.4743115	0.96948143	0.49319452	1.17326726	0.55934267	0.04293456	0.53930444
A_51_P165182	St6galnac2	-1.2762789	-1.272306	-1.2642734	0.81171258	0.43146716	1.2132755	0.76900723	0.48474065	0.10265519
A_52_P301804	Smarcd1	-0.759806	-0.5329357	-1.7653975	1.03769502	0.66137007	1.16067206	0.75704138	0.18517711	-0.7438164
A_51_P515056	Neu2	-1.2321062	-1.249188	-1.2548789	0.60565428	0.3678188	1.10595769	0.96930874	0.81575	-0.1283165
A_52_P21353	St6galnac2	-1.2472394	-1.2450522	-1.2548382	0.70714077	0.36129961	1.17398183	1.03879041	0.48112828	-0.0152111
A_52_P40345	Atp1b1	-1.296937	-1.3024296	-1.3201845	0.77635021	0.96033598	0.21918755	0.67966634	0.54339514	0.74061587
A_51_P408227	Lgals9	-1.1204492	-1.1167464	-1.3530166	1.23459299	1.27804714	0.20975024	-0.0885431	0.48268475	0.47368029
A_51_P303079	Il22ra1	-0.9455304	-0.7961195	-1.4050517	1.20347586	1.25896108	-0.7036062	0.67333513	0.71847146	-0.0039357
A_51_P476518	Rnf186	-1.1446357	-1.1446338	-1.1446491	0.68462283	1.77757583	-0.0554286	0.21061092	0.58164788	0.23488975
A_51_P173022	Abcc6	-1.1166923	-1.1418104	-1.1510055	1.27089681	1.05152452	0.84725584	0.73536654	-0.3530932	-0.1424423
A_51_P508956	Tnk1	-1.2055783	-1.2075665	-1.2296561	1.02820019	0.99196146	0.87992436	0.4784242	-0.3663751	0.63066585
A_51_P454008	Khk	-1.2036519	-1.2011543	-1.2126493	1.1758182	1.3649686	0.51935345	0.28197784	0.08717315	0.18816425
A_51_P267634	Tkfc	-1.0654084	-1.0647353	-1.0689984	1.14031845	1.71738307	0.28361546	0.42034547	-0.0920504	-0.27047
A_51_P373599	Sh2d4a	-1.2916827	-1.3021742	-1.3161878	0.8047618	0.90363612	0.83799441	0.18460523	0.5264446	0.65260263
A_51_P453657	Prss8	-1.1594722	-1.1595524	-1.1600409	1.01738761	1.23887372	1.10771521	0.0533766	-0.2552346	0.31694686
A_51_P325152	Baiap2l2	-1.2582452	-1.2583946	-1.2616259	0.86107566	0.93155747	1.11731773	0.52799957	0.32381606	0.01649919
A_52_P532074	Fbp2	-1.2775023	-1.2775007	-1.2775241	0.82699688	1.13357166	0.86059455	0.46598296	0.39210566	0.15327546
A_52_P385606	Tmc4	-1.1603015	-1.1659611	-1.1779885	0.93677199	0.62156502	1.54447855	0.05538902	-0.1386957	0.48474218
A_51_P425772	Pbld1	-0.7620936	-0.7608216	-0.7631168	1.12487389	0.62643701	1.97530326	-0.5468425	-0.4487492	-0.4449905
A_52_P598447	Dennd2d	-1.3162175	-1.3052892	-1.3303206	0.90673774	0.53770516	0.85600231	0.41847521	0.53029292	0.702614
A_51_P257058	Neurl3	-1.2742501	-1.2750021	-1.2787195	1.16733613	1.00805142	0.58354867	0.29892104	0.45671736	0.31339712
A_51_P205129	Ppargc1b	-1.2038389	-1.1806142	-1.2047424	1.34678867	1.02463273	0.86001207	-0.0062565	0.04804787	0.3159706
A_51_P156434	Itga3	-1.0753186	-0.868335	-1.2438371	1.63396986	1.14719714	0.34886743	-0.5144003	0.34427524	0.22758129
A_51_P156438	Ppargc1b	-1.2033818	-1.1395372	-1.1796938	1.4904395	1.17645214	0.36659663	0.08756693	0.3302294	0.07132817
A_52_P505277	Clca3b	-1.0281565	-1.0222214	-1.0435078	1.42128067	0.68316492	1.11143031	0.22405591	0.52588457	-0.8719307
A_52_P446625	Gstm3	-0.9950713	-0.9933656	-0.9504408	1.16714549	1.77487692	0.51388329	-0.5836781	0.02055289	0.0460972
A_51_P389779	Gpr39	-1.1243653	-1.092041	-0.8616976	1.49641977	1.03521058	0.89726231	-0.549502	-0.326682	0.5253952
A_51_P353095	Ppargc1b	-1.2127054	-1.207634	-1.1699594	1.41668787	1.03591764	0.67398862	0.02310929	0.03298207	0.40761322
A_51_P149126	Tmem79	-1.2276104	-1.2311516	-1.1982296	1.01669559	0.96460401	0.89338153	0.06626871	-0.1511322	0.86717388
A_51_P117995	Adat3	-1.1243277	-1.2605348	-0.6467271	1.02829129	1.78989345	0.36971918	0.06886711	-0.5004009	0.27521946
A_51_P273921	Gpd1	-1.3006035	-1.3018439	-1.2985638	0.94162975	0.96066088	0.79081537	0.34797847	0.40155295	0.45837375
A_52_P200465	Hdhd3	-1.3083098	-1.1842384	-1.2296933	1.36346581	0.83692346	0.61886581	0.42061787	-0.0215183	0.50388691
A_51_P122321	Khk	-1.2663556	-1.2661522	-1.2656319	1.1089346	1.13788168	0.58401541	0.41664794	0.2582349	0.29242518
A_52_P681310	Gpx2	-1.1373769	-1.1376675	-1.1375589	1.13026353	1.27242516	0.01390807	0.56500493	0.8515745	-0.4205729
A_51_P112405	Upf1	-1.013364	-1.5508795	-0.0745613	1.12836064	1.64773995	0.11798224	0.01777261	0.37462442	-0.6476751
A_51_P322612	Tmco4	-1.1707345	-1.1778454	-0.9215249	1.11145	1.72150104	0.41200589	-0.0949284	0.12326982	-0.0031936
A_52_P112139	Gpd1	-1.2509873	-1.2510733	-1.2506913	1.09657972	1.1913079	0.52865716	0.43581265	0.46151164	0.03888289
A_51_P237383	Tmco4	-1.1445678	-1.1572406	-0.9554896	1.06109046	1.75833836	0.43867969	-0.0663071	0.13558385	-0.0700872
A_52_P102045	Vars	-1.3286492	-1.5905389	-0.2503344	0.77991567	0.05132159	1.522409	-0.2637957	0.7146855	0.3649864
A_51_P293938	Dalrd3	-0.9876806	-1.2810337	-0.6302299	1.30190867	0.12864482	1.75683322	-0.0857333	-0.3845289	0.18181984
A_52_P262511	Alas1	-0.9597197	-1.0299163	-0.7490892	1.23116144	1.01026908	1.47091928	-0.780432	-0.3508936	0.15770111
A_52_P126158	Alas1	-0.8968985	-0.9348153	-0.7320893	1.28273699	1.25782338	1.27450657	-0.8534625	-0.4502137	0.05241247
A_52_P684050	Bricd5	-0.7995319	-1.0568755	-1.448818	1.32834073	0.75714279	0.6346057	0.52562191	0.78014413	-0.7206299
A_51_P262171	Clps	-1.1588273	-1.1598936	-1.1595104	0.9690849	0.61315084	1.57285583	0.16324341	0.3434962	-0.1836
A_51_P150905	Gata5	-1.2743956	-1.2744406	-1.2745008	0.69889585	0.46851998	1.08191178	0.34965637	1.04639163	0.17796139
A_51_P265348	Snx1	-1.1058469	-1.1568489	-1.5029831	0.94208477	0.14334848	1.08016233	0.65744401	0.80933224	0.13330701
A_52_P359621	Casp1	-1.2369092	-1.236515	-1.2456257	0.78910779	0.53698373	1.38425248	-0.0146683	0.65032825	0.37304598
A_52_P616392	Entpd8	-1.2034713	-1.2707496	-1.2708646	0.98242112	0.39794922	1.27152705	0.10813353	0.69819739	0.28685723
A_51_P448427	Pigr	-1.185436	-1.1854377	-1.1854365	0.94732647	0.45213119	1.4779423	-0.2384014	0.55987384	0.35743783
A_51_P270741	Slc22a1	-1.2073333	-1.2063191	-1.2072173	0.94582357	0.79643249	0.97628163	0.28674401	0.96963153	-0.3540436
A_51_P270733	Serinc5	-1.4619645	-1.1617902	-0.9722042	0.38460981	0.80701447	1.32070649	0.33246694	0.93754312	-0.1863819
A_52_P18559	Clstn1	-1.3580185	-1.3868217	-0.8471622	0.73857648	0.54708731	0.96409985	-0.3107447	1.1991844	0.45379915
A_51_P265351	Clstn1	-1.4653376	-1.4132736	-1.0098083	0.76931597	0.48203823	0.70463709	0.45659957	1.06768036	0.40814822
A_51_P143152	Omp	-0.9487888	-1.223478	-0.8976191	1.25745846	-0.0396731	0.97226844	0.00463282	1.40870502	-0.5335056
A_51_P133670	Naif1	-0.5429678	-0.4924595	-0.7011281	0.62795769	1.55346622	-0.1144037	-1.354924	-0.4337712	1.45823046
A_51_P340668	Tmco4	-0.3227195	-0.4126109	0.19578648	1.42819425	1.6787579	0.2774033	-1.1363368	-0.8829456	-0.8255291
A_51_P261560	Vars	-1.0544128	-1.0352865	1.81565319	0.31808506	-0.5274076	1.05653851	-0.9669248	0.27343887	0.12031614
A_51_P367136	D17H6S53E	-0.7456539	-0.694194	2.58513344	-0.1717298	-0.076898	-0.0858393	-0.3488489	-0.2942451	-0.1677244
A_52_P289835	Adal	-1.1300254	-1.0683201	0.86549464	1.00225143	1.38698593	0.03128714	-0.0860124	0.33414727	-1.3358085
A_51_P172344	Adal	-1.385878	-1.4473694	0.822533	0.90041082	0.83088412	0.72845048	0.36583184	0.22909099	-1.0439539
A_51_P298933	Fuca1	-1.6186722	-1.7383894	0.89894992	0.3063852	1.01155145	0.04584404	0.26384879	0.50500453	0.32547774
A_51_P139030	Fuca1	-1.6638171	-1.7214464	0.33842291	0.64997079	1.10323043	0.09252296	0.44963231	0.47853238	0.2729517
A_51_P466285	Slc16a3	-0.897208	-0.881639	-0.7015953	1.26779979	1.54186321	-0.9536365	0.95366911	0.01939068	-0.3486439

Supplementary Table 1: Genes associated with ISC-high DMRs

Probe_ID	Gene_symbol	MEF_1	MEF_2	MEF_3	ISC_1	ISC_2	ISC_3	iISC_1	iISC_2	iISC_3
A_52_P432881	Slc19a3	0.29466142	-0.7967017	-0.355135	0.16118356	1.53938851	0.83708777	-1.2783573	0.88079972	-1.282927
A_52_P283724	Wfikkn2	-0.4216514	-0.618857	-0.5349136	0.22327418	2.42459166	-0.3212601	-0.7969117	0.5154468	-0.4697189
A_52_P334301	Nim1k	1.7810332	-0.9109212	-0.5619781	-0.8471218	0.7018804	0.85314807	-0.8556326	-0.7870413	0.62663345
A_51_P194498	Wfdc1	1.80368853	0.35007056	-0.4228472	-1.560942	0.78585661	0.39910499	-0.8657949	-0.670441	0.18130441
A_52_P242213	Trpm3	0.81672601	-0.9557091	-0.63419	0.61839854	2.13501364	-0.4200946	-0.3859778	-0.7629948	-0.4111718
A_51_P312615	Hist1h1t	-0.4807337	-0.2512189	0.37500865	0.8537885	0.83662138	-0.9626509	1.14944005	-1.9290513	0.40879627
A_51_P345867	Hsf4	0.05713782	-0.6313332	0.11921077	0.75737615	0.70714448	-0.4846646	1.7430094	-0.5570317	-1.7108491
A_51_P437152	Lrp2bp	-0.6066634	-0.5785269	2.33756924	0.9110197	-0.489772	-0.0741175	-0.4317978	-0.5087939	-0.5589175
A_52_P472353	Tenm2	-0.344544	-0.347728	2.66597268	-0.3469158	-0.3037924	-0.3469262	-0.343677	-0.2854197	-0.3469696
A_51_P166266	Ticam2	-0.4263842	-0.4441327	-0.1883424	-0.4417204	0.09190062	-0.3415665	2.62368951	-0.4322095	-0.4412344
A_51_P423743	Cldn6	-1.0038175	0.07271536	-0.7392315	1.32224455	-0.3778203	1.97222642	-0.141215	-0.4315617	-0.6735403
A_52_P570543	Sdk2	-0.5764224	-0.6667118	-0.7137863	0.55833247	1.29502367	1.86301992	-0.2101308	-0.8443707	-0.7049541
A_52_P273812	Ifitm5	-0.3326942	-0.7907289	-0.1167776	1.91852573	0.62765212	-0.4441016	-1.2524988	-0.6212252	1.01184836
A_51_P417257	Ppp1r2	0.32752567	-0.854208	-1.092129	1.02146465	0.83622627	0.42554377	-0.7388038	-1.2852999	1.35968044
A_51_P252596	Sorbs2	-0.8954503	-1.0127959	-1.0070827	1.94498322	0.8539562	-0.1027668	0.14045363	-0.5140977	0.59280028
A_51_P231184	Anpep	-1.0878116	-1.1694949	-1.1752755	1.22132515	0.43586067	0.8066542	-0.4286193	0.16232682	1.23503441
A_52_P424650	Sorbs2	-0.9999264	-1.0267853	-1.0414189	1.74649803	0.14143675	0.43137617	-0.0722019	-0.3840675	1.20508905
A_52_P389431	Tbx3	-0.52335	-0.3479056	-0.4689867	0.05276327	-0.13122	0.07105147	-0.6165482	-0.607069	2.57126478
A_52_P625508	Cpt1b	-0.8878445	-0.6555319	-0.6004419	0.57668348	1.39643156	1.60645514	-0.6418414	-1.0415828	0.24767226
A_51_P232913	Cpt1b	-0.9714877	-0.7071912	-0.4706577	0.71629696	1.07590845	1.54327431	-0.543153	-1.2650215	0.62203141
A_52_P112676	Ccdc28b	0.10838836	-0.8175284	-0.8670871	-0.9854171	-0.1289696	0.10283898	-0.5666359	1.20735859	1.94705221
A_52_P230773	Fgf22	-0.1105659	-0.7732106	-0.9021032	-0.6568665	-0.3339716	-0.7578137	1.63008624	1.65514536	0.24930013
A_51_P130595	Smpd5	-0.2622886	-0.7130725	-0.7725461	-0.6853792	-0.6077804	-0.6342471	1.8675363	1.38491487	0.42286267
A_52_P644320	Sema3e	-0.3309833	-0.3449189	-0.3388217	-0.3441083	-0.3256313	-0.3270797	-0.3217389	-0.3332981	2.66658019
A_51_P193682	1700012B09Rik	1.40840099	-0.7269428	-0.6737166	-0.482698	-0.482698	-0.5721129	-0.1192543	-0.3727572	2.02177877
A_52_P312204	Nxph1	0.07612692	-0.6250114	-0.5917256	-0.5128661	2.52953157	-0.5483371	-0.2035188	-0.4099923	0.28579278
A_52_P387069	Trpm3	-0.0013282	-0.6810926	-0.6333986	-0.5379278	0.66614393	-0.5822061	-0.2243817	-0.4213708	2.41556182
A_51_P226711	Ptf1a	-0.0730884	-1.0683378	-0.9378898	-0.569752	-0.0895786	1.48014003	1.72545491	0.19314277	-0.660091
A_51_P394014	Qprt	0.73830454	-1.3992394	-1.2929475	-0.4334265	1.75196744	0.71133754	0.1655069	-0.0682234	-0.1732796
A_52_P97229	Trpm3	-0.3229563	-0.3398173	-0.3399593	-0.338773	-0.3302894	2.66661701	-0.3267816	-0.3307351	-0.337305
A_52_P432245	Cbln4	-0.4810219	-0.4856051	-0.6477632	-0.4260022	0.22048998	-0.5114733	2.46056247	0.46398847	-0.5931751
A_52_P67775	Cdh8	-0.3433509	-0.3562839	-0.3558065	-0.2083055	-0.348104	-0.3535584	-0.3444457	2.66367609	-0.3538212
A_52_P517247	Iqsec3	-0.2156048	-0.5243183	-0.5029862	2.62344453	-0.3063012	-0.4305564	0.05713434	-0.2634685	-0.4373435
A_52_P133578	Gpr158	2.0553293	-1.1363641	-0.6844002	-0.9639828	0.2990932	-0.3580663	0.8309045	0.27678471	-0.3192983
A_52_P612337	Zfp536	2.27097179	-1.1532619	-0.9805538	0.19382673	-0.2534799	-0.5537187	0.32693822	0.18584278	-0.0365653
A_51_P413075	C130060K24Rik	2.51496659	-0.8086948	-0.6819864	-0.2039252	-0.2177749	-0.4332978	0.31992256	-0.0756698	-0.4135403
A_52_P334542	Nim1k	2.3692583	-0.9645555	-0.7873084	-0.1460462	-0.3403766	-0.5084699	0.5427162	0.174759	-0.339977
A_51_P245564	Phyhipl	-0.1681889	-0.457513	-0.4491497	-0.3246445	-0.270535	-0.3992262	2.65303162	-0.2090587	-0.3747156
A_52_P381468	Nxph1	-0.2933691	-0.363578	-0.352355	-0.3298632	-0.323071	-0.3405152	2.66610231	-0.318682	-0.3446687
A_52_P90786	C130060K24Rik	2.38240716	-0.8412648	-0.7024973	-0.3226088	-0.168143	-0.4550188	0.71544573	-0.0960408	-0.5122794
A_52_P484769	Trpm3	2.42511109	-0.900578	-0.729695	-0.2501449	-0.1279195	-0.4410346	0.52302164	-0.0213886	-0.4773721
A_52_P80555	Nrg3	0.12712915	-0.6881872	-0.6496051	-0.4174739	2.47970701	-0.5260652	0.40577171	-0.1979473	-0.5333292
A_51_P186141	Thsd7b	-0.1084006	-0.9815209	-0.7923227	-0.136374	0.26480393	-0.4703883	2.35422722	0.45280407	-0.5828286
A_51_P193686	1700012B09Rik	-0.9458656	-0.3828236	-0.5588034	-0.9137787	0.16320582	1.76201521	0.55123803	1.24027044	-0.9154583
A_51_P348947	Fam135b	-2.3825105	0.17942349	0.07923371	-0.2099093	0.67421577	0.54475296	1.13172168	0.31468545	-0.3316132
A_51_P177071	Nrg3	-2.3641075	-3.65E-05	-0.2628544	0.0671953	0.88000915	0.55887368	1.05822158	0.28515401	-0.2224552
A_51_P456826	Slc19a3	-1.8221341	0.00194751	0.59654348	-0.6343502	1.29929282	0.22765062	0.66136786	0.7539738	-1.0842918
A_52_P995564	A930018M24Rik	-1.9090548	-0.2822522	0.04478694	-0.1463965	0.31171047	-0.1207233	1.90333175	0.59843595	-0.3998383
A_52_P325330	Khdrbs2	-1.9925385	-0.3959266	-0.3543217	-0.0742733	0.9220745	0.28327334	1.50104496	0.58080836	-0.4701411
A_51_P472249	Slc7a7	-1.2783989	-1.2271288	-1.2304527	0.77628995	1.04664774	1.1870206	0.06466722	0.24166951	0.41968538
A_51_P267080	Tymp	-0.8351537	-0.6979016	-0.7965072	0.45497697	1.3475057	1.88180754	-0.4483422	-0.5045475	-0.4018381
A_52_P428735	Lrp2bp	-0.9216603	-0.8967255	-0.7712465	0.12794542	2.13284384	0.95755394	-0.2807285	-0.4288908	0.08090845
A_51_P514405	Slc2a5	-0.8595477	-0.859372	-0.8590483	-0.0551744	2.00082297	1.21777116	-0.3984798	-0.33754	0.15056812
A_51_P210227	Dlc1	-1.0511238	-1.1561234	-1.0275702	0.47453101	1.54038549	1.06015136	-0.3571807	0.76814267	-0.2512124
A_51_P281543	Gpr158	-1.681488	-1.1136201	-1.0486448	0.5421129	1.11434767	0.79710995	0.42251143	0.57055809	0.39711286
A_51_P205334	Tnfrsf8	-1.2896309	-1.1446994	-1.0636223	0.80350196	1.00959343	1.17014519	-0.0510027	0.87381887	-0.3081041
A_51_P267622	Gjb2	-0.9555668	-1.3369983	-1.3258224	0.92412196	0.89813066	1.03021373	0.27016186	0.80629981	-0.3105405
A_52_P214102	Mthfr	-0.856677	-0.9067768	-0.9410462	0.66506739	0.26206492	2.16232188	-0.0899869	0.25255595	-0.5475233
A_52_P382886	Gjb2	0.0546626	-1.6921574	-1.7048455	0.51147561	0.82722801	0.93896603	0.2500743	0.45841422	0.35618212
A_51_P122170	Rdh7	-1.003232	-1.0031303	-1.0032493	0.83647107	1.26396999	1.56319494	-0.3006712	0.03192731	-0.3852806
A_52_P561224	4932438H23Rik	-1.2450413	-1.336791	-1.3493913	0.41384525	0.75551868	0.87212646	0.66530013	0.88340064	0.34103247
A_51_P290914	3110070M22Rik	-0.7315381	-1.4003602	-1.3938974	-0.3668165	1.20014708	0.98165172	0.54888844	0.73267357	0.42925143
A_51_P227345	Dpep1	-1.1664753	-1.1883741	-1.1884371	-0.4857985	1.00455899	0.95639626	0.3103191	0.9614877	0.79632299
A_51_P291637	Hspb9	-0.9963057	-1.2397133	-1.4187488	0.20103416	1.27048511	1.04511639	0.04345762	0.78162098	0.31305349
A_51_P246317	Mt2	-0.8900606	-1.33887	-1.3810917	-0.3688185	0.92575764	1.01996823	0.74982284	0.43097867	0.85231353
A_51_P212741	Scn2b	-1.0195327	-1.0887784	-1.098543	0.31691412	0.6567893	0.51131874	-0.475075	0.34474005	1.85216692
A_52_P342443	Fblim1	-1.8271402	-0.7318714	-0.5525672	1.521953	0.22439496	1.10752411	0.45227825	-0.0536723	-0.1408993
A_52_P570861	Klhl5	0.04987968	-0.3224969	-1.6687487	1.51430367	0.61700061	0.6984044	0.28889833	0.1764941	-1.3537352
A_52_P299535	Pkdcc	-1.1599533	-1.2062798	-1.2140572	1.61332725	0.52622288	0.27516017	0.84617873	0.19834637	0.12105491
A_52_P331727	Thsd7b	-0.689478	-0.9012251	-0.8889987	1.60311054	1.25038797	0.51809802	0.60451624	-0.6155916	-0.8808194
A_51_P112223	Gsta4	-1.1413161	-1.1496109	-1.1473056	1.2644693	0.2692033	0.42011904	0.85754756	1.1133666	-0.4864732
A_51_P202340	Pou5f1	-1.1344205	-1.4348795	-1.141305	1.33773668	0.31312211	0.51517663	0.98474954	0.46992459	0.08989545
A_52_P45841	Gorasp1	-1.1796271	-1.2259193	-1.3410486	1.46245836	0.48369172	0.55861471	0.28015478	0.68270542	0.27897008
A_52_P488908	Hes7	-1.1073655	-1.1201673	-1.3087517	1.33263188	1.27559377	0.27066653	0.33720939	0.44719732	-0.1270144
A_51_P389945	4932438H23Rik	-1.259078	-1.2887617	-1.2858323	1.10431802	0.39562383	0.58852058	0.10807339	0.93317779	0.70395842
A_52_P548470	Shank2	-1.1870897	-1.089533	-1.0428561	0.98222278	0.58891138	0.68238506	0.22984855	1.45163865	-0.6155275
A_52_P11402	Lrrc24	-0.8906837	-1.3248301	-0.8893165	1.66605516	-0.0876214	-0.4096142	1.19331002	0.30195431	0.44074651
A_52_P403157	Sorbs2	-1.3070185	-0.9464228	-0.9648358	1.45113854	-0.2961606	-0.2682166	1.19924225	0.320759	0.81151449
A_52_P183368	Tbx3	-0.5671667	-0.4902359	-0.4810827	-0.4530243	-0.4689392	-0.3401506	0.32687298	-0.0858622	2.55958871
A_51_P476538	Inafm2	-1.4874125	-1.0544085	-1.2080557	0.19173285	0.46233152	0.17623321	1.16794429	0.74044328	1.01119148
A_52_P262209	Atp2a3	-1.1876525	-1.1174535	-1.1383497	0.33411562	-0.0980954	0.04706542	1.62210522	1.00055555	0.53770931
A_52_P29765	Cmc1	-1.5034	-0.8884703	-0.8892922	0.70640343	-0.5733701	0.12959426	1.34798525	0.6087991	1.06175051
A_51_P185906	Abi3	-1.1919648	-1.1206898	-1.1107885	0.62192299	-0.1299781	-0.0020855	0.36885349	1.64344896	0.92128119
A_51_P324834	Evpl	-1.0276518	-1.0262882	-1.0273379	-0.0733142	-0.2065528	-0.004405	0.62748834	1.93689986	0.80116165
A_51_P167803	Pde1b	-1.2377269	-0.9001267	-0.7223078	0.81463283	-0.5780036	-0.4098901	1.31451194	1.47795449	0.24095584
A_52_P623058	R3hdm4	-1.4906249	-0.8822242	-0.8797727	0.76217881	-0.0246079	-0.1866106	1.58033982	1.05219766	0.06912414
A_52_P503663	Ffar4	-0.9094995	-0.9134046	-0.9133346	-0.1271599	-0.4617597	-0.4793593	1.6061325	1.25577326	0.94261197
A_52_P383862	Cmc1	-1.1525507	-1.1787299	-1.1942754	0.71493605	-0.0777033	-0.0959282	0.96528844	1.43354986	0.58541316
A_51_P324838	Evpl	-1.0129658	-1.0141371	-1.0147242	-0.0121388	-0.2346517	-0.1157731	0.60762225	1.93082538	0.86594306
A_51_P351273	Tcea3	-0.7509008	-1.139955	-1.1484109	0.62210612	-0.5273518	-0.370161	1.20037518	1.46924066	0.64505754
A_52_P295104	Smim5	-1.1254017	-1.1281823	-1.1279327	0.08162326	-0.1614719	-0.0415789	1.21591714	1.31711958	0.96990756
A_52_P161297	Tcea3	-0.8596904	-1.1500295	-1.1444075	0.60482095	-0.4713009	-0.2379265	1.31469905	1.39997915	0.5438556
A_51_P233367	Fzd10	-1.9622325	-0.3679301	-0.9382322	0.45009581	0.63982108	0.08567885	1.16668413	1.06501128	-0.1388963
A_51_P364168	Lrp5	-2.1651936	-0.1823475	-0.5022416	0.27427209	0.45544565	-0.1396061	1.05623446	1.26674939	-0.0633129
A_52_P627761	Ethe1	-0.4084638	-0.7730795	-0.7286986	-0.3601932	-0.3919663	-0.2134431	2.25633385	1.04888656	-0.4293759
A_51_P117618	Ethe1	-0.4778019	-0.7765484	-0.7619164	-0.3724784	-0.2837466	-0.2166619	2.2838169	0.98198077	-0.3766441
A_51_P479444	Ihh	-1.0506082	-1.051395	-1.0516834	0.49159633	-0.0889158	-0.0994651	1.39916476	1.5666287	-0.1153223
A_51_P171086	Gucy1a2	-0.4059539	-0.916626	-0.7720564	-0.2882437	0.30650609	-0.3725168	2.36448517	0.53655875	-0.4521532
A_51_P393968	Vsx2	-0.5563938	-0.9056728	-0.7105061	-0.0510974	0.28943021	-0.4171299	2.35935238	0.51485844	-0.5228412
A_51_P380451	Pla2g10	-0.55898	-0.5590813	-0.5590613	-0.4350935	-0.3596999	-0.3999007	2.52729127	0.43423493	-0.0897094
A_51_P109050	Paqr4	-1.1096857	-1.1672295	-1.1640833	0.17112016	0.20359112	0.28710553	0.74767846	1.81431162	0.21719163
A_52_P214927	Zmynd8	-0.9026379	-1.2187921	-1.1729652	-0.2951861	-0.026329	0.33819562	1.25666641	1.51062929	0.51041901
A_51_P287691	Kremen2	-0.8284142	-0.8243501	-0.8303019	-0.5828114	0.17852896	-0.0668051	1.4446006	1.83702528	-0.327472
A_51_P381297	Pax8	-1.0461521	-1.1036765	-1.1009591	-0.1332057	0.33326883	0.22935764	1.22308176	1.67113731	-0.0728522
A_51_P495700	Ccdc28b	-0.7978107	-1.306922	-1.1503761	-0.267435	0.73902807	0.45626162	-0.250411	1.53515027	1.04251484
A_51_P107140	Krt35	-0.8295354	-0.8763583	-0.8427723	-0.5432389	-0.1913654	-0.3232108	1.29076705	0.43326653	1.88244741
A_51_P221512	Ppp1r2	-1.222413	-0.5197266	-0.9675598	1.25187214	1.71082229	-0.6483441	0.41682662	-0.335544	0.31406648
A_52_P247994	Thsd7b	-0.8690027	-0.8551101	-0.8561377	1.23316684	1.79636152	0.31119081	0.43909909	-0.7091112	-0.4904565
A_51_P476169	Sfrp5	-1.7197025	-0.9892208	-1.1720891	0.34327595	0.57952077	0.81817429	0.8727561	0.59848913	0.66879617
A_52_P233411	Insc	-1.0408073	-1.060973	-1.0418668	-0.2644355	0.53869392	0.6102938	1.93179955	0.46990212	-0.1426068
A_51_P225781	Insc	-1.0378771	-1.0260903	-1.0355035	-0.2902081	0.54173641	0.65206496	1.92189037	0.50003452	-0.2260472
A_51_P104392	Rpp25	-1.2711344	-1.0777194	-1.0249269	0.07689526	0.71075847	1.04109595	1.03019523	-0.5742857	1.08912149
A_52_P143037	Gabra5	-0.5399148	-0.3274965	-0.3415938	-0.3455075	-0.2728192	-0.2851755	-0.2357178	-0.308584	2.65680899
A_51_P151316	Gabra5	-1.6847602	-0.7038269	-1.0992898	0.0617553	1.42332702	0.47388293	0.78132234	-0.0222008	0.76979005
A_52_P138540	Ppp1r2	-1.7460403	-0.7546202	-0.8085163	-0.2841926	1.34733953	0.67327311	0.95840332	-0.0613034	0.67565675
A_51_P473043	Ppp1r2	-1.6706995	-0.8788071	-0.7199837	-0.3515657	1.52325413	0.61525253	0.70825444	0.0035743	0.77072059
A_52_P164615	Ppp1r2	-1.5493987	-0.7328988	-0.7159647	-0.2467056	1.98541442	0.3442789	0.4646004	-0.0101928	0.46086684
A_52_P661327	Phyhipl	-0.7623303	0.07813561	0.2359098	-0.3577476	-0.8264894	-0.8357535	1.96477654	1.21622844	-0.7127296
A_51_P190454	Zfp37	0.66827313	0.24420695	0.19750657	-0.9292931	-1.4024188	-1.1320283	0.40191135	1.77483791	0.17700432
A_51_P168894	Ano1	-0.290971	-0.2365763	-0.3023901	-0.1501192	-0.7594466	-0.7279375	0.75767384	2.37195608	-0.6621892
A_52_P625215	Wfikkn2	0.63617217	0.44720749	0.38870749	0.43596529	-1.3221914	-1.5220122	0.73519377	1.20245828	-1.0015008
A_52_P37104	Mthfr	-0.9706075	0.66529161	-0.3930771	-1.6619579	0.15899021	0.2314491	-0.4446589	0.70377194	1.71079851
A_52_P228059	Nudt4	-1.0323134	-0.1429996	-0.1617876	-1.1290158	-0.5957537	-0.6942446	1.06337637	1.24651753	1.4462209
A_51_P342031	Mkrn3	-0.3695582	0.91398361	0.49473929	-0.9848316	-1.2355353	-1.1757132	0.20219592	0.55665804	1.59806139
A_52_P71353	Psd3	-0.5209575	0.13234224	0.51609624	-0.3125206	-1.2506625	-0.9601312	0.36276141	-0.1373204	2.17039237
A_52_P682330	Psd3	-1.1573277	0.51547383	0.65475143	-0.2713648	-0.764461	-1.3433213	0.22003236	0.31413216	1.83208494
A_52_P505277	Ubxn10	-0.7263555	-0.5570538	-0.3651777	-0.5377414	-0.1506234	-0.6827737	2.39202187	0.72124372	-0.0935401
A_52_P305246	Nudt4	-0.7031662	0.22306131	0.20745487	-0.5658844	-1.1054348	-1.4236543	0.93536342	1.28999202	1.14226811
A_52_P496608	Psd3	-0.9206148	-0.2230914	-0.5110033	0.00911135	-0.6837905	-1.0770051	0.884605	0.46977268	2.05201598
A_51_P275123	Psd3	-1.1964873	-0.0281964	-0.1653541	-0.01209	-0.6350916	-0.9927839	0.46312274	0.37727998	2.18960063
A_52_P180092	Egfem1	-0.3742171	0.22849538	-0.4462253	2.60207479	-0.4428919	-0.44396	-0.4388462	-0.4365003	-0.2479294
A_52_P226559	Kcnk9	-1.5684298	1.62950897	1.51829547	-0.1050935	-0.3479657	-0.4623103	-0.4615704	-0.0936512	-0.1087835
A_51_P309370	Fblim1	-1.9904639	-0.147421	0.03784088	1.57463645	-0.3147811	0.61767836	-0.3779682	0.905073	-0.3045945
A_51_P214503	Frem2	-0.406935	-0.3145888	-0.3168944	-0.0711964	-0.4109091	-0.4153761	-0.4077911	2.65100502	-0.307314
A_51_P130254	Psd3	-0.0728283	-0.1913581	0.63901183	1.49300891	-1.0055626	-0.9546015	-1.0148599	1.49044355	-0.3832539
A_51_P164504	Apoc1	-2.0084849	0.33762592	0.12287235	0.18887402	1.51216872	0.93134199	-0.287305	-0.7499375	-0.0471556
A_51_P421551	Cdcp2	-0.7681992	0.66617687	0.31698663	-0.7589775	2.01133878	0.7638857	-0.7190361	-0.7461847	-0.7659905
A_51_P151968	Eif2b5	0.45085175	0.62002661	0.84128206	-1.4022679	0.90941645	1.17187234	-1.0328595	-1.1375487	-0.4207731
A_52_P638439	Ppp1r2	-0.6535029	0.03871617	0.04346109	-0.8426566	2.29064064	0.26695648	-0.3520672	-1.1387243	0.3471765
A_51_P322138	Hspb2	-0.5855737	1.52036981	0.81347193	0.67415094	-0.4379136	0.5605369	0.11603553	-1.0396255	-1.6214524
A_52_P291960	Mthfr	-1.1962695	1.4892043	1.69968943	-0.9508239	1.94E-05	0.07621452	-0.4839105	-0.0750234	-0.5591003
A_51_P298196	Uggt2	-0.5620009	2.482145	0.64272047	-0.4819955	-0.4077271	-0.4402745	-0.330911	-0.4249587	-0.4769979
A_52_P374075	Csmd2	-0.332982	2.66666619	-0.3343174	-0.3337407	-0.3322549	-0.3336367	-0.3331137	-0.3329375	-0.3336833
A_52_P59400	A830018L16Rik	0.63887645	2.46733792	-0.5867062	-0.4768597	-0.4600855	-0.5205533	-0.1423087	-0.4022745	-0.5174265
A_51_P305217	Hoxd1	-0.1516305	2.1985324	-0.9431583	-0.472735	-0.2243614	-0.6639436	1.02269174	-0.0661025	-0.6992927
A_51_P472574	Fgf22	-0.5246018	0.41490494	-0.4895231	-0.5187662	-0.1812294	-0.5562396	2.51860761	-0.0685371	-0.5946153
A_52_P208250	Uggt2	-0.5287321	2.62303374	-0.4787128	-0.4715654	-0.1826305	-0.3276107	0.03906194	-0.2363513	-0.4364929
A_52_P83097	Kcnq5	-0.2101118	1.94960535	-0.547174	-0.9849926	-0.296631	0.13959665	1.33967122	-0.6025526	-0.7874112
A_51_P220665	Unc5d	1.00545734	0.38506398	0.2792117	-0.7424037	-0.7334617	2.0126333	-0.7270346	-0.7374583	-0.742008
A_52_P65480	Pcdhga4	0.92870011	-0.2729072	0.43942156	-1.0236463	-0.9898784	-1.0190208	1.3279628	-0.6583014	1.26766958

Supplementary Table 1: Genes associated with ISC-high DMRs

Probe_ID	Gene_symbol	MEF_1	MEF_2	MEF_3	ISC_1	ISC_2	ISC_3	iISC_1	iISC_2	iISC_3
A_51_P302942	Rasl10a	1.63059209	0.12620632	0.40420384	-1.0419328	-1.0781709	-1.0881386	-0.491605	0.37952454	1.15932056
A_51_P130332	Hspa12b	2.03405444	0.86188185	0.86969353	-0.6231657	-0.6300334	-0.6218152	-0.6313798	-0.6278999	-0.6313359
A_51_P395405	Klhl5	1.98758327	0.9014809	0.89270297	-0.5721695	-0.5913174	-0.4921235	-0.7242581	-0.6188004	-0.7830982
A_52_P622614	Nfatc4	2.11255679	0.73436813	0.84841816	-0.5970028	-0.5892039	-0.6303535	-0.6406871	-0.6278295	-0.6102662
A_52_P384250	Shc1	1.4810184	1.26398734	1.24432664	-0.6276795	-0.6156073	-0.6764897	-0.6855482	-0.6583546	-0.7256531
A_51_P218975	Kcns3	2.09293946	0.72886986	0.84249415	-0.7835063	-0.5905148	-0.3824311	-0.8023776	-0.4665174	-0.6389563
A_52_P219233	Loxl3	1.43289081	1.22676704	1.33280179	-0.6863748	-0.6853497	-0.5796153	-0.6833023	-0.6796831	-0.6781345
A_51_P355802	Rassf2	2.33815973	0.59568945	0.29834275	-1.0191565	-0.6655939	-0.3683005	-0.4345038	-0.3185676	-0.4260696
A_52_P502577	S1pr3	1.15700985	1.41589045	1.41589045	-0.6662312	-0.6640064	-0.6564191	-0.6671	-0.6665659	-0.6684681
A_51_P322658	Plcxd1	1.14190092	1.16919742	1.04989254	-1.1284482	0.0536907	0.17228308	-0.0625015	-1.0456009	-1.3504141
A_52_P357829	Gli2	1.00609758	1.4030244	1.55095617	-0.663087	-0.6562361	-0.6516099	-0.6630183	-0.6630007	-0.663126
A_51_P372112	Gpc6	0.84732362	1.38793583	1.67514796	-0.6598997	-0.6242399	-0.6483086	-0.658382	-0.6595149	-0.6600623
A_51_P405227	Nradd	0.88247285	1.50741933	1.53836045	-0.6845894	-0.6460517	-0.6615537	-0.5669412	-0.687253	-0.6818636
A_51_P312885	Gramd1a	0.90289343	1.53266539	1.50083674	-0.6686178	-0.6506605	-0.6468029	-0.6407535	-0.6675591	-0.6620018
A_52_P91152	Sema3e	1.13454111	1.54288777	1.30143619	-0.6655069	-0.6622557	-0.6631494	-0.6602218	-0.6633789	-0.6643524
A_52_P723443	Dzip1	0.80441669	1.53000082	1.5714055	-0.6573868	-0.6485025	-0.650192	-0.6433413	-0.651712	-0.6546884
A_51_P461364	Gpx7	1.22636928	1.3659162	1.40336198	-0.6659481	-0.6659385	-0.665938	-0.6659358	-0.6659416	-0.6659456
A_51_P441687	Lrrc10	1.20170189	1.29693792	1.48562195	-0.7776029	-0.6455566	-0.6269363	-0.5899541	-0.6516765	-0.6925353
A_51_P134972	Shkbp1	0.56776223	0.64852659	0.78699766	-2.3116947	0.66120409	0.51859753	0.08745064	-0.7246657	-0.2341783
A_52_P121192	C1qtnf5	2.33108336	0.53197882	0.56707505	-0.5874273	-0.5559253	-0.570677	-0.5709882	-0.5715549	-0.5735645
A_52_P342159	Nfatc4	1.72170701	1.09299998	1.12177756	-0.6576094	-0.6522771	-0.6529874	-0.6568177	-0.6593253	-0.6574675
A_51_P373911	C1qtnf5	2.32801775	0.53844951	0.56876087	-0.5781371	-0.5653346	-0.568028	-0.5755919	-0.5744375	-0.573699
A_51_P173459	Nim1k	1.59921491	1.11861397	1.2461684	-0.7171643	-0.5469101	-0.6719871	-0.7157912	-0.6506819	-0.6614626
A_51_P320125	Wasf3	0.76148051	1.6440453	1.47482044	-0.742806	-0.5759903	-0.5699892	-0.7454715	-0.6518279	-0.5942614
A_52_P125467	Reep2	0.72530613	1.5609267	1.58718801	-0.6993015	-0.5593706	-0.6468089	-0.6955947	-0.684702	-0.587643
A_51_P125725	Sh3gl3	0.23391003	1.72820623	1.66348264	-0.6158306	-0.5865025	-0.6027203	-0.6152028	-0.6049714	-0.6003713
A_51_P223724	Khdrbs2	-0.3322671	2.66666555	-0.3323574	-0.3336845	-0.3326888	-0.3326965	-0.3349405	-0.3342699	-0.3337608
A_51_P354706	Lefty1	0.47622596	1.49363778	1.54589695	-0.4790787	0.08227491	-0.1852542	-1.0597685	-0.9752361	-0.8986981
A_51_P135222	Dlc1	0.55184846	1.29579222	1.45539808	-0.6629127	0.22247862	0.19562786	-1.1845969	-1.2627449	-0.6108907
A_52_P495565	Efnb3	0.37979361	1.50583547	1.670905	-0.3513363	-0.507441	-0.2842112	-1.0449276	-1.0755583	-0.2930597
A_51_P218953	Zfp536	0.23011261	1.70861694	1.6823836	-0.6334784	-0.626543	-0.5790701	-0.633264	-0.6345587	-0.5141991
A_51_P408946	Ccne1	0.94685567	1.39607174	1.49235526	-0.8328643	-0.3361988	-0.2380259	-0.8071914	-1.032529	-0.5884732
A_52_P796682	Ccne1	0.74037993	1.48730626	1.47894467	-0.8589749	-0.1697054	-0.120673	-0.8183799	-1.0811708	-0.6577268
A_51_P436342	Hoxb5	-0.1901304	0.42232444	0.55041557	-0.6166029	-0.6142856	-0.6315304	2.34949178	-0.6345915	-0.6350911
A_52_P140686	Unc5d	0.3727916	0.64234057	0.39136988	-0.704033	-0.6387794	-0.6704308	2.17186406	-0.7707883	-0.7943347
A_51_P144957	Tram1l1	1.57314273	1.17204114	1.23130266	-0.6579363	-0.6637016	-0.663715	-0.6636514	-0.6637161	-0.6637661
A_51_P345366	Psmb8	2.36820887	0.23859234	0.21383961	-0.2159545	-0.496376	-0.3143653	-0.0310084	-0.4678576	-1.2950791
A_51_P345367	Psmb8	2.3186231	0.05979598	-0.0436177	-0.1550954	-0.4203044	-0.1616896	0.14804812	-0.222535	-1.5232252
A_51_P386780	Mthfr	0.87713205	1.48915798	1.39846437	-0.7940939	-0.4030472	-0.4611647	-0.8225729	-0.1171304	-1.1667453
A_52_P949440	Tgfb1i1	0.82562365	1.52183648	1.56541882	-0.6568175	-0.6504614	-0.6506296	-0.6553512	-0.6482576	-0.6513617
A_52_P380418	Rarg	1.20242631	1.28003601	1.33137021	-0.1575051	-0.5000237	-0.8991838	-0.9940604	-0.2614262	-1.0016334
A_51_P506961	Cldn9	-0.1953684	1.43389739	1.88858989	-0.4066492	-0.3425222	-0.8060313	-0.556974	0.03167279	-1.046615
A_52_P30803	Gm973	0.10076677	0.43027101	0.40153341	-0.4679009	-0.6932091	-0.699628	-0.6944811	2.3370594	-0.7144114
A_52_P576208	A930018M24Rik	-0.4141631	0.84353275	0.98774514	-0.7493462	-0.7190619	-0.4634646	-0.6914752	1.95261178	-0.7463785
A_52_P616600	Rusc2	0.46721334	1.44834749	1.04211556	-0.9646659	-1.0523541	-0.1755318	-0.5852321	0.95180189	-1.1316943
A_51_P178692	Enc1	0.0722768	1.65995466	0.97976634	-0.3889521	-1.0518034	-0.7218652	0.48120929	0.41693652	-1.4475229
A_52_P311068	Plxnb2	0.09353271	1.61728721	0.96216999	-0.6948764	-1.2185831	-0.6980489	0.43853693	0.66581512	-1.1658336
A_52_P552665	Fzd7	0.22407124	1.65988722	1.51938117	-0.4550711	-0.4872203	-1.0959919	0.11882355	-0.4916331	-0.9922467
A_51_P438967	Gpnmb	0.51740845	1.69227228	1.57513046	-0.6309887	-0.630996	-0.6309964	-0.6298461	-0.6309924	-0.6309915
A_52_P706060	Mex3a	0.96068255	1.46219916	1.43769235	-0.5183194	-0.8195121	-0.9503902	-0.1696322	-0.7908579	-0.6118621
A_52_P541175	Marveld1	1.25132734	1.31574003	1.4234647	-0.6151348	-0.7342695	-0.7301188	-0.576109	-0.627078	-0.707822
A_52_P134317	Purg	1.07263239	1.43598634	1.46522143	-0.6581911	-0.7248588	-0.6667299	-0.6076269	-0.6294453	-0.6869882
A_51_P126437	Enc1	0.25919752	1.70865719	1.59286203	-0.3270149	-0.8480545	-0.7636551	-0.2895562	-0.4479775	-0.8844585
A_51_P292008	Gpx3	0.11037231	1.68075067	1.75110068	-0.5475223	-0.5877991	-0.6229181	-0.5664785	-0.5845844	-0.6329212
A_51_P195244	Mfap4	2.42007725	0.40580083	0.44369589	-0.5448827	-0.5448902	-0.5451508	-0.5449319	-0.5448326	-0.5448857
A_52_P439407	Sema3e	0.89541722	1.70918641	1.31166636	-0.655515	-0.6556126	-0.6556273	-0.6555868	-0.638392	-0.6555363
A_52_P678942	Plxnb2	1.85005365	0.99712359	0.96931248	-0.5149796	-0.8665161	-0.7917975	-0.6312767	-0.2938505	-0.7180693
A_51_P486668	Uggt2	1.39156219	1.32656855	1.25865433	-0.5579335	-0.8074941	-0.8203836	-0.5958543	-0.5139047	-0.6812148
A_51_P461416	Kcnq5	1.33573412	1.3663618	1.28689129	-0.492958	-0.7110879	-0.7260368	-0.638085	-0.6949004	-0.7259191
A_52_P297773	Uggt2	1.28170924	1.42955183	1.27567016	-0.5376709	-0.7487215	-0.7555319	-0.6404483	-0.6040317	-0.700527
A_51_P340336	Pcbp3	0.99436041	1.44319882	1.5075368	-0.7030796	-0.7251571	-0.7225137	-0.7082534	-0.648252	-0.4378403
A_51_P490148	Nphp4	1.59697358	1.14625678	1.22727239	-0.6676517	-0.670779	-0.6708684	-0.6705584	-0.6705657	-0.6200797
A_52_P47660	Tenm2	0.4749502	1.85263542	1.3721984	-0.4838253	-0.7336572	-0.7389508	-0.7234847	-0.730562	-0.289304
A_51_P483908	Dctn1	-0.1163561	1.52487173	1.53053423	0.62393682	-1.0921262	-0.8331169	-0.8459912	-0.3139161	-0.4778363
A_52_P27003	Uggt2	1.10482979	1.23002112	1.36873972	0.25665581	-1.1195969	-0.8866588	-0.6597178	-0.5616417	-0.7326313
A_52_P1042732	Akap5	-0.3418984	1.59293466	1.88109375	-0.2146688	-0.5632059	-0.7072258	-0.5206975	-0.7014796	-0.4248525
A_51_P468876	Dixdc1	0.99757308	0.99852554	1.05362158	1.02417811	-1.1491102	-1.0166774	-0.2328837	-0.9740331	-0.7011939
A_52_P421220	Dzip1	1.32721582	1.34887157	1.32371639	-0.6532387	-0.6697587	-0.6654254	-0.6761443	-0.675696	-0.6595406
A_51_P479352	Klhdc9	1.90587698	0.96925155	0.93424747	-0.3314591	-0.7008645	-0.7299545	-0.786217	-0.6952817	-0.5655991
A_51_P478598	Nfix	2.218634	0.64243459	0.71270463	-0.5114764	-0.6626973	-0.6180103	-0.6364081	-0.6548869	-0.4902943
A_52_P148428	Nfix	2.40438076	0.37408835	0.49070245	-0.4582857	-0.4937758	-0.5686425	-0.6136184	-0.7098302	-0.4250189
A_51_P366344	Tgfb1i1	1.09706205	1.41971635	1.46316895	-0.6631085	-0.6633858	-0.6634241	-0.663465	-0.6634653	-0.6630986
A_52_P326354	Erf	1.37148135	1.18183695	1.36718932	-0.6758863	-0.5725	-1.0009864	-0.3669613	-0.8538867	-0.450287
A_52_P224829	Gpc6	1.18225595	1.36945313	1.43941177	-0.6681649	-0.6616281	-0.6682935	-0.6681212	-0.6682495	-0.6566636
A_52_P653678	Cdh8	1.84806616	1.11134484	0.38469044	0.49848327	-0.7686052	-0.7684962	-0.7682006	-0.7689872	-0.7682955
A_51_P503883	Plekhg4	0.58582437	1.47374324	1.66939509	-0.5960585	-0.7073998	-0.4274392	-0.2738825	-1.0836671	-0.6405157
A_51_P142107	Fzd7	2.1369173	0.746925	0.79006316	-0.6048818	-0.6291229	-0.6096243	-0.5865225	-0.6405543	-0.6031996
A_51_P317321	Iffo1	1.36873323	1.28439339	1.34584761	-0.6702996	-0.6705699	-0.6701976	-0.6537476	-0.6737903	-0.6603692
A_51_P328652	Acot7	1.14216175	1.32140213	1.47822635	-0.6230465	-0.7996045	-0.7440824	-0.4608223	-0.8884542	-0.4257804
A_52_P196458	Dzip1	1.19649	1.35021171	1.44484012	-0.6319644	-0.6881919	-0.6693193	-0.6533074	-0.6887995	-0.6599593
A_51_P156394	Fam135b	2.49581526	-0.5757423	0.03379765	-0.5025984	-0.456669	-0.6191847	0.42878157	-0.1941087	-0.6100913
A_51_P506015	Zmynd8	1.70275496	0.2766761	0.40462136	-0.5432839	-0.9957978	-1.688206	0.81120507	-0.1642358	0.19626591
A_51_P122290	Zmynd8	1.42331092	0.59755534	0.62499219	-0.9436924	-1.122626	-1.4527377	0.89464234	-0.2975953	0.27615066
A_51_P413614	Abca4	2.65216134	-0.3766752	-0.3144581	-0.3842093	-0.3807512	-0.3819696	-0.0619494	-0.3696851	-0.3824635
A_51_P371876	Zmynd8	2.55951139	-0.0402918	0.11712955	-0.6532809	-0.5048243	-0.5711427	0.01533781	-0.4562667	-0.4661723
A_52_P5491	Frem2	0.1684747	2.29974726	0.67091881	-0.6758865	-0.6561534	-0.6715014	-0.6401266	0.17762461	-0.6730974
A_51_P308298	Myl9	1.18476034	1.38352594	1.4234046	-0.6755273	-0.6634094	-0.6742459	-0.6550018	-0.6517661	-0.6717404
A_51_P319572	Rusc2	0.82311762	1.54649469	1.53999075	-0.7115844	-0.6495872	-0.6981022	-0.6121982	-0.5849585	-0.6531725
A_51_P380709	Dok5	0.13744492	1.55611502	1.85905829	-0.5928311	-0.5917	-0.5924424	-0.5916063	-0.5917999	-0.5922385
A_51_P187954	Tmem132d	-0.4344976	0.78087809	2.431901	-0.4767372	-0.4489678	-0.4736524	-0.4495027	-0.4564347	-0.4729867
A_51_P137523	S1pr3	1.17078103	1.43421905	1.38513905	-0.6673114	-0.6669171	-0.6672562	-0.6541397	-0.6671523	-0.6673625
A_52_P417819	Gpnmb	0.32555071	1.69827172	1.6583474	-0.6153788	-0.6136114	-0.6158074	-0.608656	-0.6119995	-0.6167166
A_51_P188108	Lama1	0.58796844	1.72733444	1.49868128	-0.6392514	-0.6306974	-0.6400575	-0.627953	-0.6345903	-0.6414346
A_51_P202942	Cox4i2	0.2524572	1.18226909	1.89595854	-0.6760008	-0.2091874	-1.2537919	0.02752704	-0.3575296	-0.8617023
A_52_P209383	Tgfb1i1	0.94742937	1.49551832	1.50570774	-0.6581897	-0.6577661	-0.6581906	-0.6581403	-0.6581716	-0.6581971
A_51_P381506	Eva1b	2.11080088	0.83471334	0.75327035	-0.6184805	-0.6205689	-0.6209521	-0.6064761	-0.6112455	-0.6210614
A_52_P460704	A830018L16Rik	2.02621576	0.14752442	1.34227152	-0.5961964	-0.6097381	-0.629399	-0.5117758	-0.5488231	-0.6200793
A_51_P290886	Cdh8	2.22957285	0.72913491	0.60932363	-0.6066212	-0.6053203	-0.6147718	-0.5377271	-0.5899735	-0.6136175
A_52_P549166	Arsj	2.16241223	0.74945305	0.73700193	-0.6106723	-0.6052458	-0.6108048	-0.6102655	-0.6010991	-0.6107796
A_51_P344461	Prrt2	2.25202063	0.55962314	0.71773845	-0.5748154	-0.4952174	-0.6737916	-0.5380275	-0.5864228	-0.6611074
A_52_P556933	Col16a1	1.90146095	1.05331917	0.89606037	-0.6479531	-0.6235546	-0.6497675	-0.6361042	-0.642853	-0.650608
A_51_P354345	Zpbp	1.69916831	1.16254685	1.07791601	-0.6803945	-0.5551668	-0.6828524	-0.663499	-0.6741655	-0.6835529
A_52_P222624	Hspb2	1.36881138	1.3524554	1.27749009	-0.6696991	-0.6694827	-0.6527452	-0.6675654	-0.6692081	-0.6700564
A_52_P649623	Sema3d	2.31312064	0.58519264	0.55995863	-0.5806786	-0.5804718	-0.5665786	-0.5706995	-0.5781812	-0.5816622
A_51_P372853	Rarg	1.74528349	1.08163285	1.07499962	-0.7878854	-0.6765197	-0.6595292	-0.552566	-0.4411566	-0.7842591
A_51_P229911	Adcy4	1.81676419	0.997277	1.08038598	-0.6957109	-0.6455284	-0.6307556	-0.6196402	-0.594775	-0.7080171
A_51_P372858	Rarg	1.70073153	1.03554435	1.16600872	-0.7840818	-0.7021058	-0.6083973	-0.5371612	-0.4094115	-0.8611271
A_52_P648524	Dio3	2.03027766	0.84279489	0.89377775	-0.6408557	-0.6389682	-0.6404997	-0.6352635	-0.57045	-0.6408131
A_52_P655008	Iffo1	1.76032932	0.98934904	1.16802516	-0.6548965	-0.654241	-0.6545785	-0.653944	-0.6448591	-0.6551845
A_51_P320452	Trhde	1.29700535	1.18721822	1.49946003	-0.7014417	-0.6650314	-0.7006955	-0.5609316	-0.653289	-0.7022944
A_52_P398099	Uggt2	1.69595837	1.14387644	1.10516652	-0.6597704	-0.6552472	-0.65976	-0.6535888	-0.6563622	-0.6602728
A_52_P522372	Aard	2.39086688	0.4493123	0.4852134	-0.5714219	-0.5538241	-0.570841	-0.504756	-0.5510071	-0.5735425
A_52_P174915	Gja1	1.6577686	1.18583091	1.11235426	-0.660458	-0.6599363	-0.6603759	-0.6565975	-0.6578087	-0.6607773
A_51_P291062	Col16a1	1.74097647	1.09326195	1.09326195	-0.6736305	-0.6732056	-0.6725036	-0.6634265	-0.6697819	-0.5749523
A_51_P307650	Nmu	2.47538568	0.17500201	0.38143698	-0.5855465	-0.5775741	-0.5826037	-0.5727753	-0.577993	-0.1353321
A_51_P196774	Prr16	1.97648542	0.82874947	0.90827652	-0.7650878	-0.7581211	-0.7645589	-0.1773439	-0.7389919	-0.5094078
A_51_P339023	Rassf2	2.10923638	0.66329252	0.70510423	-1.0267651	-0.7904092	-0.7778757	-0.440736	-0.0829988	-0.3588484
A_51_P194377	Pcbp4	1.45303805	1.2578724	1.2821897	-0.7047237	-0.6874623	-0.6884511	-0.6380432	-0.6322041	-0.6422159
A_51_P131879	Pcdhga4	1.55963638	0.87161218	1.08986157	-1.0862366	-0.8706708	-1.0248152	-0.456438	0.4167984	-0.4997479
A_51_P172952	A830018L16Rik	1.74826709	1.14272096	0.87637365	-0.7948802	-0.7802315	-0.7956716	-0.723673	0.01420935	-0.6871148
A_51_P435731	Arhgef40	1.68637403	1.03620352	1.13619435	-0.8212467	-0.8078878	-0.8505142	-0.5952947	-0.1926472	-0.5911814
A_52_P659762	Eid2	1.3686448	1.2832659	1.32381896	-0.8102659	-0.7722172	-0.7747319	-0.5608251	-0.5462417	-0.5114479
A_51_P115346	P2rx6	1.98463192	0.80688172	1.00007389	-0.6683007	-0.6424785	-0.6645391	-0.6554338	-0.5272638	-0.6335716
A_51_P374571	Igfbp6	1.40385475	1.29687554	1.29735854	-0.668059	-0.6700781	-0.6690885	-0.6629328	-0.6641602	-0.6637703
A_51_P408506	Icam1	1.83626337	1.05372851	0.98784949	-0.6897412	-0.7168783	-0.6925755	-0.6337886	-0.4841381	-0.6607196
A_51_P427934	Megf8	1.82706733	1.04412536	0.98181484	-0.6714287	-0.8763575	-0.7885879	-0.511018	-0.4926489	-0.5129665
A_51_P395473	Tns2	1.67598351	0.68394901	0.77513244	-1.053621	-1.0772368	-1.0936569	-0.0056009	0.61817068	-0.52312
A_51_P172054	Gas6	1.43534032	1.13370812	1.18398898	-0.8064676	-1.0496887	-0.9667505	-0.1561568	-0.0391877	-0.7347861
A_52_P418477	Tpm2	0.88792272	1.47405621	1.5693493	-0.6557018	-0.6558966	-0.6558437	-0.6544573	-0.6537415	-0.6556873
A_52_P251615	Arhgef40	1.2002125	1.39367878	1.39797918	-0.6957813	-0.7070746	-0.6898269	-0.6251864	-0.6215297	-0.6524715
A_51_P419925	Arhgef40	1.26838536	1.32017	1.405955	-0.6984384	-0.7198149	-0.6941961	-0.6142795	-0.5964196	-0.6713619
A_52_P481988	Ddr2	1.00525783	1.43285071	1.52037054	-0.6913265	-0.6931636	-0.6979902	-0.5628912	-0.668766	-0.6443416
A_52_P532227	S1pr1	1.27342796	1.41239099	1.31151621	-0.6695697	-0.6696214	-0.6697035	-0.6493059	-0.6695448	-0.6695898
A_52_P315976	Tpm2	0.98626864	1.46706388	1.50466986	-0.6601044	-0.6601581	-0.660181	-0.6591244	-0.6584492	-0.6599852
A_51_P410430	Cfap45	1.07624421	1.57861983	1.31156367	-0.6879146	-0.6879453	-0.6880133	-0.6387803	-0.5931694	-0.6706048
A_51_P483438	Sema3e	0.45003686	1.7897137	1.50266861	-0.6384541	-0.6369953	-0.6393143	-0.6073025	-0.5898997	-0.6304532
A_52_P223269	Zfp37	0.98899547	1.19267968	0.87126996	-1.1016109	-1.1633177	-1.0800786	-0.2429396	0.94008325	-0.4050815
A_51_P427476	Mxra8	2.10289668	0.80481866	0.79951153	-0.6220261	-0.621931	-0.6209385	-0.6026156	-0.6189899	-0.6207259
A_52_P613241	Icam1	1.76392286	1.0538604	1.10081582	-0.6673174	-0.68622	-0.6620017	-0.6581813	-0.5797886	-0.6650902
A_52_P590936	Mthfr	1.4864992	1.14910295	1.3203613	-0.7416423	-0.7367362	-0.7288091	-0.6682845	-0.3502228	-0.7302686
A_51_P498152	Sat2	1.93486154	0.81678071	0.86398597	-0.8727804	-0.8502124	-0.7772845	-0.2995989	-0.0007856	-0.8149665
A_51_P125825	Dzip1	1.78604186	1.10115011	1.02351707	-0.6601213	-0.659648	-0.64661	-0.6594109	-0.659847	-0.6250718
A_52_P678056	Eva1b	1.7918375	1.06602619	1.05147375	-0.6590566	-0.653204	-0.6512001	-0.641724	-0.6520933	-0.6520594
A_52_P26409	Rarg	1.47037268	1.44643572	1.02479473	-0.8992579	-0.6650477	-0.6383837	-0.444753	-0.6779461	-0.6162147
A_51_P501840	Dnajb3	1.24269981	1.3518609	1.40154773	-0.6677336	-0.7075267	-0.6539768	-0.6453747	-0.6610663	-0.6604303
A_52_P564620	Gli2	0.89530987	1.57696354	1.34502908	-0.9948116	-0.9281088	-0.3155064	-0.242349	-0.6726123	-0.6639144
A_51_P321531	Purg	0.76604844	1.5359529	1.58309529	-0.7330418	-0.7103277	-0.6926673	-0.5200293	-0.629273	-0.5997576
A_51_P267796	Ddr2	1.03479423	1.4758346	1.45414465	-0.7024738	-0.7019798	-0.7018431	-0.5631792	-0.6766222	-0.6186755
A_51_P465281	Lgals1	1.24361304	1.37641296	1.37641296	-0.6892876	-0.6838617	-0.6776354	-0.6384268	-0.6680502	-0.6391773
A_51_P208435	Ston1	-0.1091736	1.88475303	1.51744081	-0.9388343	-0.5634116	-0.5609744	-0.1328775	-0.5352348	-0.5616876

Supplementary Table 1: Genes associated with ISC-high DMRs

Probe_ID	Gene_symbol	MEF_1	MEF_2	MEF_3	ISC_1	ISC_2	ISC_3	iISC_1	iISC_2	iISC_3
A_52_P144297	Tspyl3	1.02308966	1.50968698	1.43248044	-0.6840988	-0.6734955	-0.6649152	-0.6360329	-0.6448859	-0.6618288
A_52_P161455	Luzp2	-0.4739318	2.26033921	1.10637643	-0.5409002	-0.51558	-0.5303085	-0.5058757	-0.5152939	-0.2848255
A_52_P413947	Mthfr	1.05792694	1.4105937	1.4939829	-0.763797	-0.722941	-0.7339983	-0.6020351	-0.6038299	-0.5359022
A_52_P484956	Nnat	0.29145599	1.6372639	1.73223821	-0.6105558	-0.6103629	-0.6104579	-0.6103332	-0.6103891	-0.6088594
A_52_P203560	Fzd10	0.25081443	1.81285116	1.52616961	-0.7114163	-0.7109381	-0.7109209	-0.7106127	-0.476234	-0.2697132
A_51_P252859	Cyr61	0.84921779	1.52915585	1.54217746	-0.6618631	-0.666034	-0.6551827	-0.6460509	-0.6475392	-0.6438813
A_51_P184853	Tenm2	1.0794049	1.3113737	1.54853559	-0.8147458	-0.7653496	-0.656263	-0.5841918	-0.7462955	-0.3724685

Supplementary Table 1: Genes associated with ISC-low DMRs

Probe_ID	Gene_symbol	MEF_3	MEF_2	MEF_1	ISC_3	ISC_2	ISC_1	iISC_2	iISC_1	iISC_3
A_52_P438342	Rabl6	0.13862301	0.45345557	0.78191843	-1.7242351	0.45345557	0.30124433	0.87519604	-1.7144634	0.43480551
A_51_P454008	Lbp	0.32116509	0.46338288	1.42943637	-0.8861302	-0.8860128	-0.3881077	1.59772905	-0.7977565	-0.8537072
A_51_P373599	Slc9a4	-0.6523378	-0.4563852	0.08091809	-0.4695449	-0.6408772	0.35486514	2.51642894	-0.3402026	-0.3928643
A_51_P354838	Krt75	-0.6292501	-1.1047926	1.27897558	-0.7981495	-0.2634749	-0.0599841	1.98340864	-0.3058693	-0.1008637
A_52_P42373	Wdr72	-0.777112	2.27784514	1.00969263	-0.582231	-0.4717234	-0.2711313	-0.4850047	-0.159712	-0.5406235
A_51_P312336	Slc14a1	0.04900723	0.01563658	2.60611869	-0.391075	-0.3537661	-0.4845984	-0.4845802	-0.4845802	-0.4721626
A_51_P325776	Csad	0.31756282	0.23321135	2.39290863	-0.5298051	-0.472067	-0.7283654	-0.724574	-0.71358	0.22470863
A_51_P343689	Slc14a1	0.01279418	-0.0033673	2.61391219	-0.3653734	-0.3462951	-0.4783103	-0.4842289	-0.4842203	-0.4649111
A_52_P646783	Fbxl22	1.07136727	1.56510535	1.07267252	0.07822717	-0.4295407	-0.8345887	-1.2423364	-0.6173492	-0.6635574
A_52_P530958	Cd2bp2	1.05167631	1.24711187	1.37770034	0.19003691	-0.2105499	-0.920699	-1.0228273	-0.8498236	-0.8626256
A_51_P263568	Prss27	1.34341047	1.45964674	0.04143153	0.34525266	0.30611716	-0.6903313	-1.0420489	-1.4870097	-0.2764686
A_51_P126626	Zfp503	1.53515228	1.5875948	0.55973515	-0.1192984	-0.3904895	-0.7209506	-0.7288271	-1.0934638	-0.6294529
A_51_P448427	Xpnpep1	1.37425572	1.12552145	0.95909899	-0.1005471	0.25158799	-1.113961	-0.7067957	-1.3997183	-0.3894421
A_51_P189905	Dad1	1.45063303	1.29124188	0.87650041	-0.488348	-0.121361	-1.5432821	-0.4735674	-0.262395	-0.7294217
A_51_P448671	Tbxas1	1.21805384	1.28777587	-0.2191695	0.86956958	-0.3952644	0.47148772	-0.8076645	-1.0085132	-1.4162755
A_51_P448664	Tbxas1	1.21270889	1.45302453	-0.1142434	0.88341705	-0.4870266	0.16094073	-0.7549288	-1.074086	-1.2798065
A_52_P598447	Klhl22	1.25911776	1.19718869	-0.7255047	0.52414151	0.4123899	0.52362215	-1.4798719	-1.0581628	-0.6529206
A_52_P86750	Rabl6	1.86976813	0.89699605	0.54016187	-0.2838497	-0.1559446	-0.0356129	-1.5058671	-0.9413226	-0.3843292
A_52_P134556	Cd2bp2	1.22810183	1.18616935	1.32630581	-0.2630186	-0.6340373	-0.1475236	-1.369012	-0.8610992	-0.4658863
A_51_P215038	Tmem59l	1.25690056	1.42907494	1.29086471	-0.5211748	-0.5782863	-0.5579719	-0.7648202	-0.7987749	-0.7558122
A_51_P463159	Casp14	0.75046441	1.55666899	1.55211432	-0.5212122	-0.6611306	-0.3895199	-0.7622397	-0.7619267	-0.7632187
A_51_P117995	Pfkm	0.67794519	0.64327439	1.42449182	0.06048878	-0.0360838	0.64453016	-0.5267407	-1.1051496	-1.7827563
A_51_P466685	Fbxl22	0.61744793	0.59925656	1.04965291	1.39765059	-1.0855605	-0.3564791	-1.6190582	-0.520353	-0.0825572
A_52_P268529	Csad	0.20210781	0.11974602	1.55200422	-0.4347872	-0.4894952	-0.8141592	-1.005266	-0.8011408	1.67099046
A_51_P270741	Syngr1	1.41342996	1.45260362	0.29928411	-0.1175841	0.1438418	-0.5613676	-0.0115527	-1.4191612	-1.1994939
A_52_P616392	Sbno2	1.20134192	1.15809062	1.57402918	-0.4820012	-0.5844007	-0.8430271	-0.4680594	-0.8872701	-0.6687033
A_51_P270733	Syngr1	1.57148333	1.46837686	-0.2694421	-0.0364561	0.02576745	-0.3957003	0.12696552	-1.3561572	-1.1348375
A_52_P359621	Luzp1	1.47058775	1.42977167	-0.0649701	0.24727538	-0.4505865	-1.6217301	0.26342885	-0.7207724	-0.5530045
A_52_P18559	Sbno2	0.1520689	0.30348801	0.75557527	0.28655507	0.07809362	-1.5914135	1.73959274	-0.5612604	-1.1626997
A_52_P405994	Orai1	0.66535393	0.28816744	0.38028699	0.00379119	-0.1926692	-2.2233982	1.50115968	-0.2670028	-0.155689
A_52_P537827	Wdr72	1.31154753	1.60938563	1.03802504	-0.6598488	-0.6598467	-0.6597753	-0.6598332	-0.6597698	-0.6598843
A_51_P262171	Irgm1	1.44714739	1.49960717	0.99041148	-0.7583599	-0.6980829	-0.5288562	-0.6112425	-0.4947346	-0.8458899
A_52_P126158	Irgm1	1.34370094	1.29017678	1.28178012	-0.89108	-0.7305766	-0.3748709	-0.5297788	-0.4007004	-0.9886511
A_51_P150905	Klhl22	1.72566218	1.57349472	0.26800209	-0.657601	-0.7797391	-0.3535304	-0.3465248	-0.4261156	-1.003648
A_51_P293938	Rasl11b	1.51158601	1.46596434	0.95980928	-0.7215767	-0.7712413	-0.5165274	-0.6948296	-0.518166	-0.7150187
A_52_P102045	Naif1	1.958008	1.54685639	-0.376311	-0.5342021	-0.5310565	-0.5075165	-0.5224376	-0.513025	-0.5203158
A_51_P246854	Acta1	1.60152571	1.57783523	0.3902251	-0.8594965	-0.836725	0.04559023	-0.3545693	-0.7435663	-0.8208192
A_52_P684050	Fam110a	1.5326496	1.29666993	1.08483463	-0.8749451	-0.8074527	-0.3828132	-0.4285947	-0.5754096	-0.8449389
A_52_P681310	Plaur	1.47343691	1.43311135	1.02275912	-0.6931625	-0.8081256	-0.7568513	-0.4411717	-0.425662	-0.8043342
A_51_P112405	Plaur	1.56304536	1.60862389	0.61788945	-0.6791624	-0.8096534	-0.7256366	-0.3982211	-0.3728354	-0.8040497
A_52_P275152	Cdc16	0.73159431	0.76690153	2.11971918	-0.5700448	-0.8479789	-0.2712386	-0.5214406	-0.6626021	-0.74491
A_51_P508349	Cdc16	1.39083361	1.33692342	1.24829849	-0.7561689	-0.8873	-0.6004843	-0.5898818	-0.5713123	-0.5709084
A_51_P165210	Arhgef7	1.52381501	1.31093247	1.11655311	-0.7944837	-0.8731876	-0.5516788	-0.5345424	-0.6765306	-0.5208774
A_52_P19532	Fgd3	1.69753443	1.45760728	0.70312718	-0.6585176	-0.657248	-0.6475543	-0.6106044	-0.7338644	-0.5504802
A_52_P507802	Lpar1	0.27527539	0.26416203	2.5084571	-0.5872031	-0.5122926	-0.5384158	-0.4749089	-0.5008675	-0.4342066
A_51_P298933	Anks6	0.86855113	0.4351308	0.33132768	-0.9689896	-1.4994396	-0.2034403	-0.9679031	1.62346148	0.3813014
A_51_P261560	Tex33	1.04935776	0.99210101	-1.1267105	-0.3660504	-0.9749886	-1.299976	-0.0871339	0.57596586	1.23743483
A_51_P143152	Luzp1	1.29034321	1.30144131	-0.6669622	-0.5508362	-1.0184288	-0.8816856	0.72646872	-0.9749886	0.77464808
A_52_P446625	Cep170b	0.21922073	0.18144677	-2.1389702	-0.747146	-0.386197	0.17807806	1.18043971	0.57418725	0.93894071
A_51_P185882	Ccdc68	-0.2540515	-0.1977274	-1.4821723	-0.6255638	-0.3048215	-0.6157638	1.36903373	1.66115742	0.44990913
A_52_P127682	Dagla	0.34008694	0.21975521	-1.8499326	-0.1837241	-0.9723976	0.07788314	0.63377885	1.73974652	-0.0051963
A_52_P370560	Vars	-1.0544128	-1.0352865	1.81565319	0.31808506	-0.5274076	1.05653851	0.12031614	0.27343887	-0.9669248
A_52_P81231	Acpp	-0.5078064	-0.5102459	2.62471968	-0.2354628	-0.0661726	-0.0556448	-0.416862	-0.4847735	-0.3477516
A_51_P488422	Adal	-1.385878	-1.4473694	0.822533	0.90041082	0.83088412	0.72845048	-1.0439539	0.22909099	0.36583184
A_52_P622694	Adal	-1.1300254	-1.0683201	0.86549464	1.00225143	1.38698593	0.03128714	-1.3358085	0.33414727	-0.0860124
A_51_P108757	Fuca1	-1.6186722	-1.7383894	0.89894992	0.3063852	1.01155145	0.04584404	0.32547774	0.50500453	0.26384879
A_52_P52263	D17Wsu92e	-0.6371716	-0.8810031	1.25178589	1.52260307	1.15096077	-0.7454578	-0.260091	-0.6675379	-0.7340883
A_52_P517905	Numb	-1.0375305	-1.0295073	-1.3847082	1.08878904	1.13296747	1.19417698	-0.0828111	-0.077069	0.19569257
A_52_P511798	Baiap2l2	-1.2582452	-1.2583946	-1.2616259	0.86107566	0.93155747	1.11731773	0.01649919	0.32381606	0.52799957
A_51_P499020	Fbp2	-1.2775023	-1.2775007	-1.2775241	0.82699688	1.13357166	0.86059455	0.15327546	0.39210566	0.46598296
A_52_P522427	Hsh2d	-0.9926348	-0.9822689	-0.9933624	0.93241977	0.84320611	1.73501746	-0.4714401	0.31637976	-0.3873169
A_51_P101765	Smarcd1	-0.759806	-0.5329357	-1.7653975	1.03769502	0.66137007	1.16067206	-0.7438164	0.18517711	0.75704138
A_52_P356068	Proz	-0.6184023	-0.7147359	-0.7242431	1.73253004	1.41699682	0.70343777	-0.6438359	-0.5707556	-0.5809918
A_51_P159673	Snx1	-1.1058469	-1.1568489	-1.5029831	0.94208477	0.14334848	1.08016233	0.13330701	0.80933224	0.65744401
A_51_P213260	Gata5	-1.2743956	-1.2744406	-1.2745008	0.69889585	0.46851998	1.08191178	0.17796139	1.04639163	0.34965637
A_51_P239737	Pigr	-1.185436	-1.1854377	-1.1854365	0.94732647	0.45213119	1.4779423	0.35743783	0.55987384	-0.2384014
A_52_P468482	Entpd8	-1.2034713	-1.2707496	-1.2708646	0.98242112	0.39794922	1.27152705	0.28685723	0.69819739	0.10813353
A_51_P469401	Tnfrsf18	-0.9101509	-0.9256716	-1.0533538	0.5078409	0.49226933	2.13700132	-0.0226438	0.12432074	-0.3496122
A_52_P229972	Slc22a1	-1.2073333	-1.2063191	-1.2072173	0.94582357	0.79643249	0.97628163	-0.3540436	0.96963153	0.28674401
A_51_P194883	Kcnq1	-1.2031743	-1.2034862	-1.2038944	0.35255293	0.34684982	1.3112446	0.17380002	0.14579161	1.28031584
A_52_P338199	Dalrd3	-1.0854187	-1.2177947	-1.4743115	0.96948143	0.49319452	1.17326726	0.53930444	0.04293456	0.55934267
A_52_P474145	Slc7a9	-1.2809611	-1.2807914	-1.2812345	0.78065468	0.5314729	0.79344048	0.46840977	0.11918053	1.14982856
A_51_P112627	St6galnac2	-1.2472394	-1.2450522	-1.2548382	0.70714077	0.36129961	1.17398183	-0.0152111	0.48112828	1.03879041
A_51_P460143	Neu2	-1.2321062	-1.249188	-1.2548789	0.60565428	0.3678188	1.10595769	-0.1283165	0.81575	0.96930874
A_51_P416408	Tmc4	-1.2064317	-1.2108344	-1.2161882	0.40827743	0.39311899	1.53691062	0.9157622	0.13785341	0.2415317
A_52_P239320	Syngr1	-1.1390078	-1.142218	-1.1611066	0.7058428	0.69877791	1.27662624	1.14996419	-0.3277072	-0.0611716
A_52_P137361	Tmc4	-1.2184099	-1.2195632	-1.2231551	0.41074442	0.3726091	1.49309015	0.93317832	0.11988282	0.33162342
A_51_P439645	1700001C19Rik	-1.1548436	-1.1647999	-1.2233212	0.65178609	0.65534237	1.49411524	0.81988743	0.12078086	-0.1989472
A_51_P512205	Unc13d	-0.9629019	-0.9432704	-1.0946902	0.03011575	0.25968465	1.11818228	1.8938255	-0.0197433	-0.2812025
A_52_P149577	Rab25	-1.2554262	-1.2546114	-1.255933	0.56061318	0.57872857	0.82306302	1.35800538	0.18813701	0.25742344
A_51_P129199	Parp10	-1.3483411	-1.3412001	-0.6659595	0.51837151	0.43259818	1.53030117	1.02722417	-0.2702641	0.11726985
A_51_P158061	Dalrd3	-0.9876806	-1.2810337	-0.6302299	1.30190867	0.12864482	1.75683322	0.18181984	-0.3845289	-0.0857333
A_52_P26216	Numb	-1.074517	-1.0626197	-1.3009757	1.16486734	0.9069565	1.31359067	0.29447915	-0.0837346	-0.1580466
A_51_P499816	Pbld1	-0.7620936	-0.7608216	-0.7631168	1.12487389	0.62643701	1.97530326	-0.4449905	-0.4487492	-0.5468425
A_52_P271200	1700001C19Rik	-1.0728404	-1.0907229	-1.1093114	0.95658327	0.63994707	1.68889321	0.35341497	-0.0098428	-0.3561211
A_51_P325071	Numb	-1.0822318	-1.1446806	-1.316818	1.33815705	0.80100228	1.09428292	0.28266358	0.20677168	-0.1791471
A_52_P662796	Dennd2d	-1.3162175	-1.3052892	-1.3303206	0.90673774	0.53770516	0.85600231	0.702614	0.53029292	0.41847521
A_52_P219415	Tmc4	-1.1603015	-1.1659611	-1.1779885	0.93677199	0.62156502	1.54447855	0.48474218	-0.1386957	0.05538902
A_51_P309158	Snx20	-0.783308	-0.7853049	-0.8234955	0.84991872	1.30827655	1.67353254	-0.1555954	-0.706629	-0.577395
A_52_P799815	Tmem171	-1.0219557	-1.0120681	-1.0438788	0.84822226	1.13960672	1.29942067	0.13798114	0.55283642	-0.9001646
A_52_P119060	Acpp	-1.0898583	-1.0870691	-1.1172502	0.64313581	0.7062482	1.85015134	0.20095234	-0.0339378	-0.0723722
A_51_P454196	Sh2d4a	-1.2916827	-1.3021742	-1.3161878	0.8047618	0.90363612	0.83799441	0.65260263	0.5264446	0.18460523
A_52_P526724	Ppargc1b	-1.2038389	-1.1806142	-1.2047424	1.34678867	1.02463273	0.86001207	0.3159706	0.04804787	-0.0062565
A_52_P12739	Dagla	-0.6987995	-0.2246655	-1.3816562	1.08887127	0.26974041	1.91467877	0.01263608	-0.7612222	-0.2195833
A_51_P279232	Dennd2d	-1.3112585	-1.3093506	-1.3257607	0.81360833	0.40205869	0.92376276	0.7470841	0.60684284	0.45301299
A_51_P128929	Alas1	-0.8968985	-0.9348153	-0.7320893	1.28273699	0.25782338	1.27450657	0.05241247	-0.4502137	-0.8534625
A_51_P269320	Adsl	-1.08366	-1.1517675	-0.7593233	1.72299456	0.69279173	1.04753044	0.16039536	-0.0378697	-0.5910915
A_52_P129756	Alas1	-0.9597197	-1.0299163	-0.7490892	1.23116144	1.01026908	1.47091928	0.15770111	-0.3508936	-0.780432
A_51_P203501	Vars	-1.3286492	-1.5905389	-0.2503344	0.77991567	0.05132159	1.522409	0.3649864	0.7146855	-0.2637957
A_52_P267700	Gkn3	-0.6827171	-0.6820146	-0.6708849	0.77633342	-0.1077695	2.37860881	-0.3310353	-0.288104	-0.392417
A_51_P376050	Epsti1	-1.1879478	-1.1856153	-1.1849991	0.77247893	0.87760425	1.2843259	0.24771943	0.74957751	-0.3731439
A_51_P295967	Proc	-1.1410908	-1.1348884	-1.0673465	1.33306243	0.76861326	1.28646765	-0.1180542	0.37667129	-0.3034346
A_51_P460459	Dfna5	-1.4757663	-1.4063042	-0.5789377	1.19893731	0.94322895	0.94843849	-0.1137801	0.51815656	-0.033973
A_52_P293222	Ppargc1b	-1.2033818	-1.1395372	-1.1796938	1.4904395	1.17645214	0.36659663	0.07132817	0.3302294	0.08756693
A_52_P203398	Proz	-0.6888959	-0.6892547	-0.6865124	1.79553354	1.22064641	0.85559094	-0.6884667	-0.4300313	-0.6886099
A_51_P497100	Lgals4	-1.3289209	-1.3289926	-1.3288694	0.61226338	0.71439855	0.83965153	0.62470947	0.65455803	0.54120192
A_52_P582374	Epsti1	-1.159323	-1.1514623	-1.1513425	0.60531295	0.79543249	1.33979609	0.48252618	0.80834737	-0.5692873
A_51_P500676	Dmbt1	-1.1259796	-1.1258503	-1.1258401	0.92173291	0.20802507	0.50649306	0.70936163	1.51901141	-0.4869541
A_51_P412391	Chst4	-1.1116195	-1.1004485	-1.0528044	1.422153	0.50464699	0.64811978	0.15870487	1.15482907	-0.6235813
A_51_P460851	Arl14	-1.2264851	-1.2265359	-1.226274	0.4745122	0.19156405	1.25230932	1.00227387	0.80660105	-0.0479655
A_52_P399106	9030617O03Rik	-1.2015538	-1.2047041	-1.1409964	0.79791897	0.27094951	0.91231011	1.38740584	0.48922825	-0.3105584
A_51_P119387	Tm4sf5	-1.2649449	-1.2649987	-1.2645276	0.93630331	0.67018078	0.87988132	0.96805732	0.39993045	-0.0598819
A_51_P424641	Sirt4	-0.8002073	-1.4099818	0.3464622	0.47644206	-0.3492972	1.66822892	0.95253629	0.19875745	-1.0829406
A_51_P234407	Tjp3	-1.3194757	-1.3194008	-1.3195278	0.86964914	0.7555531	0.52610028	0.46128033	0.50755815	0.8382633
A_51_P337935	Slc7a15	-1.0330123	-1.0464944	-1.0892507	1.2043794	0.42689397	-0.3103063	-0.1343611	0.30562149	1.67652989
A_52_P259714	Kmt2b	-0.6180544	-0.6460739	-1.2665574	1.06567757	0.98851601	-0.5570924	0.66223081	-0.9688238	1.34017753
A_52_P257426	Eps8l2	-1.2755204	-1.2662428	-1.3089126	0.81547996	0.75579977	0.48218283	0.60349366	0.09139178	1.10232778
A_51_P195066	Sema4a	-1.3195878	-1.3197832	-1.319136	0.72752649	0.57319063	0.63031459	0.63751607	0.42186624	0.96809295
A_52_P203368	Klb	-0.5942281	-0.8124644	-0.7682362	0.02977958	2.34401413	-0.7669621	0.32277106	-0.1763471	0.42167316
A_51_P447216	Rasa2	-1.5653578	-1.6091036	0.36899281	1.02768043	0.56923635	-0.5502594	0.4210787	0.8549724	0.48276013
A_52_P179640	Fuca1	-1.6638171	-1.7214464	0.33842291	0.64997079	1.10323043	0.09252296	0.2729517	0.47853238	0.44963231
A_51_P206165	9130230L23Rik	-1.1996017	-1.1998356	-1.1986504	1.35371241	1.01735577	0.2265443	-0.0747482	0.83933612	0.23588729
A_51_P486810	Gpx2	-1.1373769	-1.1376675	-1.1375589	1.13026353	1.27242516	0.01390807	-0.4205729	0.8515745	0.56500493
A_51_P520412	Rabl6	-1.4356994	-1.4820098	-0.5384501	1.32144553	0.98707324	-0.2251197	0.35792989	0.36917447	0.64565583
A_51_P439626	Map2k2	-0.8471739	-0.9086716	-0.6540953	1.39043537	1.89281714	-0.5738522	-0.3829544	-0.0872063	0.17070125
A_51_P438820	Pycard	-1.2236781	-1.2229835	-1.1969561	1.2332272	1.31886706	0.19674178	0.23374868	0.29786931	0.36316364
A_51_P438821	Pycard	-1.2461119	-1.244542	-1.2240842	1.21450331	1.21450331	0.23827418	0.27473055	0.28864741	0.48407936
A_52_P184362	Il22ra1	-0.9455304	-0.7961195	-1.4050517	1.20347586	1.25896108	-0.7036062	-0.0039357	0.71847146	0.67333513
A_52_P371949	Eif6	-1.0969396	-1.2113453	-1.227009	0.6113587	1.74916573	0.19001786	0.32023317	0.45681117	0.20770725
A_51_P144090	Slc16a3	-0.897208	-0.881639	-0.7015953	1.26779979	1.54186321	-0.9536365	-0.3486439	0.01939068	0.95366911
A_51_P279183	Tkfc	-1.0654084	-1.0647353	-1.0689984	1.14031845	1.71738307	0.28361546	-0.27047	-0.0920504	0.42034547
A_51_P117881	Leap2	-0.7264482	-0.7222299	-0.7263336	1.02065356	2.22222986	-0.1534329	-0.4920321	-0.4212108	-0.0011959
A_51_P490767	Khk	-1.2036519	-1.2011543	-1.2126493	1.1758182	1.3649686	0.51935345	0.18816425	0.08717315	0.28197784
A_51_P185693	Slc2a2	-0.9223028	-0.9223068	-0.9223172	1.14454278	1.86029451	0.58568139	-0.3612883	-0.451323	-0.0109806
A_52_P595140	Hsd17b13	-1.1657231	-1.1657327	-1.1657556	0.88650884	1.56944192	0.06852422	0.1186897	0.04581174	0.80823488
A_52_P395164	Pex16	-1.3569919	-1.3548023	-1.0465982	1.01619338	1.24894109	0.46128051	0.38569406	0.14309666	0.50318668
A_51_P397920	D17Wsu92e	-1.3463076	-1.2933031	-1.0881789	0.97673521	1.26568334	0.606537	0.13829809	0.13203135	0.60850461
A_52_P550932	H1f0	-1.336122	-1.27816	-1.1875192	0.98763087	1.12925378	0.61560714	0.09201723	0.32241556	0.65487658
A_51_P515018	Hsd17b13	-0.8388799	-0.8388914	-0.8384719	0.74765659	2.20491839	-0.0406476	-0.341461	-0.4449232	0.39070008
A_52_P241732	Pex16	-1.3698311	-1.3690839	-1.065681	0.95843068	1.19784687	0.38005504	0.53033225	0.19274968	0.54518151
A_51_P256902	Abcg5	-1.2685579	-1.2683903	-1.2678366	0.85238854	1.02126242	0.28714417	0.59349814	0.06778745	0.98270405
A_51_P170959	Proz	-0.6816335	-0.679723	-0.6760789	1.68547091	1.6124973	0.44838556	-0.5813932	-0.5808769	-0.5466482
A_51_P511112	H1f0	-1.3208122	-1.3149129	-1.1700721	0.89186053	1.1915734	0.67526621	0.09031435	0.37771779	0.57906494
A_52_P72434	Khk	-1.2663556	-1.2661522	-1.2656319	1.1089346	1.13788168	0.58401541	0.29242518	0.2582349	0.41664794
A_52_P409988	Slc2a2	-0.9046261	-0.9040049	-0.9045948	1.01469724	1.85677822	0.82442703	-0.3714505	-0.4851195	-0.1261067
A_51_P291492	Slc7a15	-1.040379	-1.0192488	-0.9023136	0.71138323	0.81884097	0.4485566	-0.5715741	-0.2598182	1.81455275
A_51_P314153	Nr2c2ap	-1.475509	-1.448304	-0.92321	0.7302069	1.01249191	0.4100699	0.36028164	0.41506553	0.91890714
A_52_P16419	Gpd1	-1.2509873	-1.2510733	-1.2506913	1.09657972	1.1913079	0.52865716	0.03888289	0.46151164	0.43581265
A_52_P96847	Smlr1	-0.9248352	-0.9248374	-0.9248201	1.41070823	1.62470707	0.67328766	-0.5113322	-0.2186108	-0.2042673
A_51_P296057	Upf1	-1.013364	-1.5508795	-0.0745613	1.12836064	1.64773995	0.11798224	-0.6476751	0.37462442	0.01777261

Supplementary Table 1: Genes associated with ISC-low DMRs

Probe_ID	Gene_symbol	MEF_3	MEF_2	MEF_1	ISC_3	ISC_2	ISC_1	iISC_2	iISC_1	iISC_3
A_51_P224013	Tk1	-1.4404081	-1.4482473	-0.6529114	0.83153378	1.01623842	1.19103481	-0.1108347	0.13617338	0.47742098
A_51_P311342	Dfna5	-1.0647961	-1.0827721	-0.7857199	0.82760493	1.78596608	1.00693873	-0.2682954	-0.3281975	-0.0907287
A_51_P184385	Pla2g12b	-1.2237417	-1.2238226	-1.2224034	0.91400461	1.06676075	0.4316823	1.13099355	-0.1150076	0.2415341
A_51_P281778	Igsf23	-1.1619148	-1.1616944	-1.1605614	0.64934615	1.19761408	0.49998116	1.34883977	-0.1598801	-0.0517305
A_52_P185119	Igsf23	-1.1539842	-1.1541833	-1.1539777	0.75277858	1.23403827	0.58093355	1.23198777	-0.2057329	-0.13186
A_52_P442234	H1f0	-1.3222158	-1.3296654	-1.2573697	0.74636814	1.05202326	0.59772077	0.77547844	0.43297985	0.30468037
A_52_P371946	Eif6	-1.2778046	-1.3147212	-1.0713037	0.7646106	0.85509689	0.55794136	1.20352506	0.57462541	-0.2919698
A_52_P85864	Eif6	-1.3225115	-1.3681948	-1.079354	0.58557132	0.85051188	0.62958471	1.17341572	0.60716615	-0.0761894
A_51_P241863	Slc5a1	-1.2757185	-1.2757317	-1.2756373	0.53201867	0.9501337	-0.001866	0.91262625	0.52909307	0.90508175
A_52_P168567	Cebpa	-1.3638335	-1.3652905	-1.2401926	0.77231798	0.79480842	0.46581886	0.80972496	0.52897705	0.59766929
A_52_P610923	Car9	-1.2372388	-1.2372766	-1.234466	0.82622382	1.15912585	-0.248391	0.75592306	0.51946466	0.6966351
A_51_P189361	Osgin1	-1.1747862	-1.1434118	-1.0421386	1.25132794	1.36547384	0.03481669	0.85812147	0.07916244	-0.2285658
A_52_P576854	Osgin1	-1.174256	-1.1344219	-1.0152085	1.25043017	1.41832059	-0.0513145	0.7720917	0.21960687	-0.2852485
A_52_P615348	Igsf5	-1.1278147	-1.1270709	-1.1273857	1.3635941	0.93588773	0.46183033	0.73540471	0.53049133	-0.6449369
A_51_P118225	Igsf5	-1.1268963	-1.1274299	-1.1283549	1.37437598	0.85871565	0.3464689	0.77220254	0.66457552	-0.6336575
A_51_P214087	Cebpa	-1.3496339	-1.3147442	-1.2245023	1.18222319	0.73885783	0.54865538	0.62411802	0.38357576	0.41145019
A_51_P465582	Hdhd3	-1.3083098	-1.1842384	-1.2296933	1.36346581	0.83692346	0.61886581	0.50388691	-0.0215183	0.42061787
A_51_P294891	Ppargc1b	-1.2127054	-1.207634	-1.1699594	1.41668787	1.03591764	0.67398862	0.40761322	0.03298207	0.02310929
A_52_P635015	Gpr39	-1.1243653	-1.092041	-0.8616976	1.49641977	1.03521058	0.89726231	0.5253952	-0.326682	-0.549502
A_51_P324583	Adat3	-1.1243277	-1.2605348	-0.6467271	1.02829129	1.78989345	0.36971918	0.27521946	-0.5004009	0.06886711
A_52_P593037	Acsl5	-1.1764972	-1.188631	-1.1257851	1.33130181	1.36501985	0.36225624	0.28889066	-0.1219257	0.26537046
A_51_P293853	Gpd1	-1.3006035	-1.3018439	-1.2985638	0.94162975	0.96066088	0.79081537	0.45837375	0.40155295	0.34797847
A_51_P166873	Tmem79	-1.2276104	-1.2311516	-1.1982296	1.01669559	0.96460401	0.89338153	0.86717388	-0.1511322	0.06626871
A_52_P163011	Naif1	-0.5429678	-0.4924595	-0.7011281	0.62795769	1.55346622	-0.1144037	1.45823046	-0.4337712	-1.354924
A_52_P322301	Esrp2	-1.2144132	-1.2077456	-1.2350994	0.63020319	0.36431013	0.23795056	1.61263221	0.51607187	0.29609022
A_51_P356467	Cldn4	-0.7996174	-0.7949322	-0.8038055	-0.0513061	-0.2329399	-0.2464218	2.36211842	0.61862446	-0.0517201
A_51_P165182	Batf2	-1.0826575	-1.0753464	-1.0811114	1.01877738	0.4767585	-0.379367	1.06513626	1.36543337	-0.3076232
A_52_P263371	Tm9sf2	-0.9989215	-0.7334414	-1.6172706	-0.0877009	0.79956266	-0.1979254	1.48547312	0.82395072	0.5262734
A_51_P186856	Krt5	-0.4852103	-0.4529905	-0.4888429	-0.2599695	-0.2463404	-0.3043642	2.6476357	-0.1524864	-0.2574314
A_51_P464530	Sept5	-1.1572807	-1.143034	-1.2406606	0.79574425	0.79951774	-0.5489212	1.09961531	0.86925154	0.52576774
A_51_P103222	Slc39a4	-1.1825862	-1.1823834	-1.1827931	0.07418363	0.19551368	0.05215077	1.42262866	1.13425723	0.66902876
A_52_P21353	Sept5	-1.1023337	-1.1225499	-1.3522727	0.97773584	0.87253265	-0.3517254	1.15402711	0.68126941	0.24331659
A_52_P78123	Dcaf12	-1.0628515	-1.1093449	-1.4287501	0.3693712	1.1257875	-0.0998688	1.27367358	0.71900015	0.21298286
A_52_P154918	Tmem72	-0.3654904	-0.6285915	-1.6439017	0.30420959	0.70226817	-0.4751327	0.43640038	1.92418026	-0.253942
A_51_P215489	Slc37a1	-1.2588224	-1.2593709	-1.2590164	0.52656044	0.56233611	0.03481526	1.02033851	1.18727726	0.44588215
A_51_P437309	Oasl1	-1.121568	-1.1275313	-1.1117156	0.54426555	0.75030147	-0.6989246	1.00681227	1.28389456	0.4744657
A_52_P563375	Lgals2	-1.312962	-1.3129961	-1.3129707	0.82250251	0.6403345	0.52877004	0.82250251	0.83901764	0.2858016
A_51_P234253	Sdcbp2	-1.1496574	-1.1477645	-1.1315869	0.3469185	0.37424791	0.23519758	1.54625248	1.15047545	-0.2240831
A_51_P178894	Usp44	-0.5132706	-0.5481603	-0.4931914	-0.3979479	2.55355985	-0.3962925	-0.1460351	0.37914836	-0.4378104
A_51_P350005	Il17c	-0.3538401	-0.3678865	-0.3331854	-0.3312217	-0.311321	-0.3195251	-0.3192184	2.66624928	-0.330051
A_52_P404273	Slc24a1	-0.6977217	-1.0869697	-0.4786047	-0.3410986	0.33441152	-0.0336526	0.53137861	2.30396076	-0.5317037
A_52_P40345	Ppp1r14d	-1.3128258	-1.312844	-1.3129238	0.78028738	0.68727897	0.22113053	0.83788996	0.71373835	0.69826837
A_51_P408227	Misp	-1.3023213	-1.3027465	-1.3034076	0.82946447	0.30984449	0.37844364	1.00698468	0.62359964	0.76013843
A_51_P263033	Muc13	-1.3063086	-1.3062985	-1.3062902	0.66287604	0.41380849	0.5419755	1.143243	0.58327767	0.57371667
A_51_P240760	B3gnt3	-1.30729	-1.309759	-1.3208361	0.8721312	0.58281273	0.64270756	0.84206924	0.7339365	0.26422785
A_51_P302377	Fgfbp1	-1.2781617	-1.2778448	-1.278325	0.7881608	0.63382599	0.04938215	1.14297753	0.50194288	0.71804222
A_51_P476518	Fam83g	-1.2775975	-1.2751633	-1.3832079	0.90583487	0.71958326	0.42706403	0.87612043	0.37574142	0.63162473
A_51_P120220	Sipa1l3	-1.3133408	-1.3281415	-1.1457343	0.4550546	0.42673615	0.25604288	0.73919356	0.52328819	1.38690121
A_51_P183414	Guca2b	-1.2729487	-1.2729783	-1.2729769	0.34980841	0.48828489	0.10144945	0.99688012	0.91009376	0.97238728
A_51_P498631	Dfna5	-0.7531225	-0.772392	-0.9357958	-0.4906385	-0.7418553	0.18099193	0.81443247	0.70392125	1.99445844
A_51_P306710	Cldn15	-1.2812165	-1.2816491	-1.2835636	0.56337057	0.09389388	0.43472285	0.88499867	0.82864001	1.04080315
A_51_P143901	Tm9sf2	-1.0745243	-0.9359238	-1.6654056	0.10460425	0.74168111	0.12311258	0.88323003	0.61369715	1.20952856
A_52_P669128	Slc5a1	-1.2831042	-1.2830558	-1.2831011	0.42507637	0.90299256	0.0876247	1.01745376	0.60957282	0.8065409
A_51_P473229	Zbtb7b	-0.742374	-0.8225671	-0.5530404	-0.4289912	0.27099622	-0.9965846	2.13547871	0.50142284	0.63565955
A_52_P10458	Skp1a	-0.6790295	-0.211128	-0.5311062	-0.0285667	0.514531	-1.9296686	0.74461849	0.60153922	1.51881034
A_51_P453657	Syt8	-0.4622903	-0.4599134	-0.3549505	-0.3955688	-0.4375806	-0.1216326	2.64415653	-0.2750486	-0.1371718
A_52_P376135	Mknk2	-1.290132	-1.2153496	-0.9379819	0.12264432	-0.3134389	0.69606885	1.45232059	0.45333189	1.03253681
A_52_P608916	Slc9a4	-0.5093219	-0.5220205	-0.511962	-0.3497719	-0.4798594	0.47799393	2.53014233	-0.4213717	-0.2138289
A_52_P600193	Myo7b	-1.3011494	-1.3011563	-1.3009715	0.44162653	0.42831084	1.14706145	0.64521022	0.47767027	0.76339791
A_51_P424298	Myo7b	-1.2987241	-1.2965894	-1.2970927	0.42031912	0.36696402	1.08417517	0.59673391	0.49684958	0.92736441
A_51_P196596	Trim2	-1.2700896	-1.2730783	-1.2816229	0.14776632	0.33567793	1.129659	0.58683723	0.65670863	0.96814175
A_52_P204331	D630039A03Rik	-1.2818814	-1.2821053	-1.2823966	0.16257398	0.31582124	1.03634983	0.72864319	0.63942755	0.96356748
A_52_P419247	Trim2	-1.2811083	-1.2762971	-1.3268049	0.25720174	0.33859666	0.98875432	0.69307964	0.66173567	0.94484228
A_51_P413866	Cfb	-1.3098858	-1.3101827	-1.308596	0.49045036	0.39696999	0.67742222	0.5373945	0.76923821	1.05718924
A_51_P457430	Adck5	-1.3923113	-1.4200924	-1.0849081	0.28617139	0.42589582	0.82791381	0.60059857	0.79094294	0.96578935
A_51_P129285	Slc17a2	-0.9264114	-1.6279069	-0.7323509	-0.1190991	0.23631706	1.47055155	-0.0934887	1.12827467	0.66411373
A_52_P609918	Ehf	-1.286082	-1.2861347	-1.2858088	0.12720956	0.55308978	1.04216522	0.58109569	0.98277374	0.57169145
A_51_P107658	Mcf2l	-1.0261629	-1.0996622	-1.0555457	-0.2256973	-0.332194	1.28531509	0.57378893	1.53736375	0.34279431
A_51_P464394	Klb	-0.9244304	-0.9916514	-0.9377105	0.01212039	-0.1729554	0.76496696	0.11692441	2.15714001	-0.024404
A_51_P279841	Blnk	-1.2328966	-1.2356182	-1.2948505	0.34365763	0.05939904	1.15207336	0.58763383	1.10114947	0.51945197
A_51_P467960	Cntd1	-0.9960322	-1.0038537	-0.8856733	0.01517188	-0.2939856	2.01963946	0.64554919	0.75399921	-0.254815
A_51_P246754	Pnpo	-1.2661299	-1.2557834	-1.294015	0.47325177	0.34482074	1.13007505	1.03579115	0.6468013	0.18518836
A_51_P100991	Gucy2c	-1.2682402	-1.2677078	-1.2692038	0.53681728	0.52189487	1.30195337	0.59945919	0.73999521	0.10503184
A_52_P540554	Pbld1	-0.3765671	-0.4128306	-0.4457149	-0.3548303	-0.2991147	2.65591288	-0.247632	-0.15066	-0.3685634
A_51_P317882	Sectm1b	-1.1307574	-1.1307401	-1.1215159	0.43962697	-0.1250833	1.0660835	1.07924977	1.2517247	-0.3285883
A_51_P317887	Sectm1b	-1.1484701	-1.1466062	-1.1466242	0.55144711	-0.0977127	1.16309279	0.83960088	1.26556138	-0.280289
A_51_P107652	Slc44a4	-1.2133576	-1.2123736	-1.2131436	0.15115557	0.1259348	0.32064459	1.48182275	0.90838656	0.65093062
A_52_P254908	Mcf2l	-1.0039197	-0.9827343	-0.9955929	-0.3478773	-0.4994217	0.62022009	0.69705288	1.82112297	0.69114989
A_51_P520084	Lypla2	-1.3195111	-1.2593651	-1.0468144	-0.043012	-0.0040818	0.54767656	1.03662931	1.14369159	0.94478697
A_51_P389636	Kcnn4	-1.2642469	-1.258357	-1.1670678	0.0606315	0.02791093	0.54126808	1.01254223	1.16103621	0.88628272
A_51_P456208	Tff3	-1.1600219	-1.1600104	-1.1600221	-0.0978011	-0.178953	0.56397088	0.71548522	1.34560011	1.13175214
A_52_P496726	Rasd1	-0.8946917	-0.877557	-0.7398491	-0.3725546	-0.3211062	-0.3567023	0.48304921	2.05844307	1.02096858
A_52_P685999	Mettl7b	-1.0591114	-1.0591076	-1.0586252	-0.2707879	-0.3996505	1.11880719	1.28360682	1.2018306	0.24303797
A_51_P196925	Cx3cl1	-1.4502948	-1.4373529	-0.726682	0.29283802	0.01939372	0.57212416	1.37778783	0.87184291	0.480343
A_52_P264571	Mcf2l	-0.9422048	-0.9452369	-0.9401364	-0.5853407	-0.6012407	0.76456596	0.8631656	1.66948682	0.71694111
A_52_P517762	Slc44a4	-1.1908394	-1.1908821	-1.190729	0.14352996	0.04565131	0.36806445	1.58483505	0.87801679	0.55235296
A_52_P634250	Lgals4	-1.2958204	-1.2958696	-1.2957769	0.36071797	0.41726048	0.71097552	1.10760738	0.86087208	0.43003352
A_52_P665119	Cdhr2	-1.3204908	-1.3242098	-1.2591185	0.36007653	0.45048999	0.96207789	1.02132023	0.56540879	0.54444558
A_51_P230589	Tnfrsf14	-1.2108314	-1.2649699	-1.2173062	0.0078211	0.30189904	0.47203402	1.41036404	0.85602309	0.64496624
A_51_P234330	Rtkn	-1.3656674	-1.426832	-1.1301316	0.35743788	0.51859267	0.55449995	1.00828334	0.73087564	0.75294146
A_52_P356204	Nostrin	-1.2212089	-1.2231971	-1.227626	0.30077133	0.24552185	0.50024782	1.5996394	0.61128252	0.41456902
A_51_P502872	2200002D01Rik	-1.1808791	-1.1824074	-1.2224578	-0.0591422	0.12965284	0.70474155	1.41958673	1.02220216	0.36870323
A_52_P1068320	Tnfrsf11a	-1.0432137	-1.020063	-1.2893225	-0.117318	-0.0202416	0.40243348	1.56195676	1.22602811	0.29974046
A_51_P139780	Pglyrp1	-1.2496995	-1.2494486	-1.249714	0.2536253	0.08265946	0.55117502	1.1967985	1.07990302	0.58470074
A_51_P464669	Nr1h4	-1.22164	-1.2198426	-1.2226532	-0.004592	0.02218982	0.55547787	1.1425079	1.05133955	0.89721265
A_52_P218470	Cdhr2	-1.3018492	-1.3023116	-1.3025562	0.46340546	0.31061789	0.79418657	1.09308774	0.63231381	0.61310556
A_51_P275435	2310007B03Rik	-1.2004686	-1.2087933	-1.2097804	0.37478878	0.07166797	0.51441179	1.64701803	0.50271628	0.50843948
A_51_P312348	Krt7	-1.2022246	-1.2019721	-1.2288537	0.08434213	-0.0348696	0.87496669	1.40500699	0.68500538	0.61859877
A_52_P410685	Krt7	-1.1945244	-1.1942374	-1.2170826	0.16413202	-0.0305824	0.88022938	1.48693482	0.69628058	0.40884996
A_51_P448741	Tnfsf10	-1.2262019	-1.2262158	-1.2269083	0.25360616	0.09910691	0.98761678	1.1588488	1.03655438	0.14359301
A_51_P434758	Fam83e	-1.3076971	-1.3079616	-1.3078429	0.5943109	0.35538193	0.6209086	0.99670217	0.89097275	0.46522532
A_51_P122906	Tnfrsf11a	-1.0855855	-1.1250606	-1.2607736	0.14740725	0.13168129	0.33104365	1.38493507	1.37444693	0.10190539
A_51_P230583	Tnfrsf14	-1.058149	-1.0320816	-1.2390812	0.18918181	-0.1945234	0.52427266	1.71812388	0.98365165	0.10860512
A_52_P435561	Prr15l	-1.271696	-1.2714314	-1.2719145	0.27933021	0.34803112	0.78250536	1.21059515	0.87833067	0.31624948
A_52_P26357	Arhgef7	-1.0375732	-0.9818895	-1.1459752	-0.0102668	0.21270716	0.1367729	2.0289358	0.67030966	0.12697911
A_51_P491916	Rassf6	-1.1720111	-1.1859273	-1.4173512	0.25037867	0.41262355	0.58283274	1.34337421	0.84837632	0.33770399
A_51_P507832	Pik3cg	-0.5276389	-0.5525154	-0.8499113	-0.5408556	-0.2393554	0.96011947	0.03870627	2.26219252	-0.5507418
A_51_P179023	Dcaf12	-0.0487654	0.24907591	-1.8662648	-1.409548	0.35348049	0.60241823	1.06350573	0.9083391	0.14775874
A_52_P46085	Mvp	-0.6392097	-0.6141268	-0.5094685	-0.4965521	-0.3277234	-0.228677	1.9938491	1.46812976	-0.6462215
A_52_P265820	Gm9887	-1.0027376	-0.7550897	-0.383967	-0.8803293	-0.1918028	0.79792422	1.09114512	1.83721666	-0.5123596
A_52_P419059	Fcgbp	-1.1403495	-1.1391647	-1.1438992	0.09672326	-0.0365362	0.90364815	-0.0917458	1.31494736	1.23637669
A_51_P482121	Edn2	-0.8012184	-0.8016208	-0.8095638	0.08272993	-0.2985426	0.19262599	-0.6234358	0.87396254	2.18506296
A_52_P272429	Ehf	-1.2800317	-1.2776374	-1.2823452	0.41704226	0.46781728	0.7673863	0.23637914	1.24333107	0.70805814
A_51_P380078	Fcgbp	-1.1586766	-1.1586778	-1.1586719	0.10868291	0.0276671	0.76688571	-0.0237518	1.30669785	1.28984458
A_52_P205877	Ehf	-1.2771941	-1.2772373	-1.2770647	0.34015599	0.54559911	0.62744237	0.30929447	1.30926901	0.6997351
A_52_P365633	Tmem91	-1.3022375	-1.4375008	-0.7818041	0.64968878	-0.2715596	0.97228748	0.19628287	1.30114018	0.67370272
A_52_P648738	St6galnac2	-1.2762789	-1.272306	-1.2642734	0.81171258	0.43146716	1.2132755	0.10265519	0.48474065	0.76900723
A_51_P416771	Ranbp3l	-0.5060652	-0.5127275	-0.4969263	0.90676735	-0.5030116	2.3656543	-0.5033095	-0.4927666	-0.257615
A_52_P633665	Dfna5	-0.6134975	2.43125704	-0.8539527	0.39702478	-0.4694969	-0.4044715	-0.2087557	0.28489833	-0.5630058
A_51_P205129	Cx3cl1	2.29021528	-0.9921338	-1.142477	0.21463619	-0.0266257	0.41849404	-0.1815879	-0.4350695	-0.1454516
A_51_P275764	Acpp	0.60187879	-1.2555141	-1.4294827	0.73121511	1.06582459	1.34359707	-0.0325016	-0.4238847	-0.6011325
A_51_P257058	Ip6k2	-0.1950334	-0.2574776	-1.5954057	1.04931753	0.44154854	1.67376685	-0.971331	0.31380948	-0.4591947
A_52_P1178881	Cdc16	-0.6097267	0.5229501	-1.0603619	1.60969399	0.79265615	0.90392352	-0.8390399	-0.1039095	-1.2161857
A_51_P148756	Usp44	0.68815526	-0.8615747	0.52353933	-0.5477697	1.36792348	1.40040487	-1.1658792	-0.5766362	-0.8281631
A_52_P532074	Zfyve27	-0.5563499	-0.4510267	-0.714223	1.13015376	0.67145725	0.46848627	-1.4882536	-0.6193612	1.55911717
A_52_P274504	Dcaf12	0.66227867	0.06352233	-1.2205255	0.85045329	0.49468425	0.9289651	-0.9262532	-1.6543356	0.80121069
A_51_P353095	Zfyve27	0.11525366	0.26722952	-0.951549	-1.3951332	-0.0530281	2.23440981	-0.0645125	-0.237964	0.08529375
A_51_P123091	Kmt2b	0.73656745	0.35836775	-1.8998959	-0.990565	0.36365346	1.16816182	-0.3648686	-0.3605019	0.9890809

Supplementary Table 2

Supplementary Table 2: Basic statistics of methylome data

Summary of mapping	ISC_1	ISC_2	MEF_1	MEF_2	MEF_3	iISC_1	iISC_2	iISC_3
Total PE1 reads	129,557,468	148,261,267	156,323,837	120,753,163	127,641,049	126,698,391	135,634,589	143,946,918
Uniquely mapped PE1	86.30%	86.40%	87.90%	87.90%	87.80%	87.00%	86.50%	86.40%
Unmapped PE1	9.70%	9.50%	8.50%	8.40%	8.60%	9.00%	9.50%	9.60%
Total PE2 reads	129,557,466	148,261,267	156,323,837	120,753,163	127,641,049	126,698,388	135,634,592	143,946,918
Uniquely mapped PE2	83.40%	82.60%	84.20%	84.00%	84.00%	84.10%	84.20%	84.10%
Unmapped PE2	12.70%	13.20%	12.20%	12.30%	12.40%	12.00%	11.80%	12.00%
Mean depth	ISC_1	ISC_2	MEF_1	MEF_2	MEF_3	iISC_1	iISC_2	iISC_3
Top strand	4.6	5.2	5.8	4.5	4.7	4.6	4.9	5.3
Bottom strand	4.6	5.2	5.8	4.5	4.7	4.6	4.9	5.2
Both strand	9.3	10.4	11.6	9	9.4	9.2	9.9	10.5
All N	4.6	5.2	5.8	4.5	4.7	4.6	4.9	5.3
All C	4.9	5.5	6.1	4.8	5	4.9	5.3	5.6
All CpG	6.3	7.2	7.4	6	6.1	6.5	6.9	7.3
All CHG	5.3	6	6.6	5.2	5.4	5.4	5.8	6.1
All CHH	4.9	5.4	6.1	4.7	4.9	4.8	5.2	5.5
Median depth	ISC_1	ISC_2	MEF_1	MEF_2	MEF_3	iISC_1	iISC_2	iISC_3
All N	5	5	6	5	5	5	5	5
All C	5	6	6	5	5	5	5	6
All CpG	6	6	7	5	6	6	6	6
All CHG	6	6	7	5	6	5	6	6
All CHH	5	6	6	5	5	5	5	6
Methylation level	ISC_1	ISC_2	MEF_1	MEF_2	MEF_3	iISC_1	iISC_2	iISC_3
all (all C)	3.7	3.8	3.8	3.9	3.9	3.7	3.6	3.6
all (all CpG)	64.3	66.3	70.1	69.6	70	63.8	61.4	61.9
all (all CHG)	1	1	1	1	1	0.9	0.9	0.9
all (all CHH)	0.9	0.9	0.9	0.9	1	0.9	0.9	0.9
LAMBDA (all C)	0.9	0.8	0.8	0.8	0.8	0.8	0.8	0.9
LAMBDA (all CpG)	0.8	0.8	0.8	0.8	0.8	0.8	0.8	0.8
LAMBDA (all CHG)	0.9	0.9	0.9	0.9	0.9	0.9	0.9	0.9
LAMBDA (all CHH)	0.9	0.9	0.8	0.8	0.9	0.9	0.9	0.9
Coverage vs read depth	ISC_1	ISC_2	MEF_1	MEF_2	MEF_3	iISC_1	iISC_2	iISC_3
1 (all N)	82.60%	83.50%	84.60%	81.70%	82.40%	81.00%	81.80%	82.60%
3 (all N)	63.80%	67.00%	72.10%	62.60%	64.90%	61.40%	63.80%	66.20%
5 (all N)	43.00%	47.70%	54.90%	41.40%	44.30%	41.40%	45.00%	48.10%
10 (all N)	9.50%	13.20%	17.30%	9.00%	10.00%	10.30%	12.50%	14.50%
